# LSD1-mediated demethylation of the DNA damage response factor ATM promotes senescence and organ aging

**DOI:** 10.1172/JCI199854

**Published:** 2026-09-15

**Authors:** Yingying Zhang, Chen Yu, Xiaoqin Zhang, Linda Xiaoyan Li, Alice Shasha Cheng, Xiaogang Li

**Affiliations:** 1 Department of Internal Medicine, Mayo Clinic, Rochester, Minnesota, USA.; 2 Department of Nephrology, Shanghai Tongji Hospital, Tongji University School of Medicine, Shanghai, China.; 3 Department of Biochemistry and Molecular Biology, Mayo Clinic, Rochester, Minnesota, USA.

**Keywords:** Aging, Cell biology, Cell stress, Cellular senescence, DNA repair

## Abstract

Aging occurs heterogeneously across organs, leading to progressive tissue dysfunction. Cellular senescence is a stress response triggered by age-associated insults, yet the mechanisms regulating senescence and organ aging remain incompletely understood. Here, we defined a role for lysine-specific demethylase 1 (LSD1) in DNA damage–mediated senescence and organ aging. LSD1 was upregulated in aged organs and senescent cells. In response to natural aging or ionizing radiation–induced DNA damage, LSD1 interacted with and demethylated ATM at lysine 3,016, as confirmed using a newly generated ATM-K3016me antibody. This modification sustained ATM phosphorylation, amplified DNA damage signaling, and delayed checkpoint recovery, promoting senescence and organ aging. Inhibition of LSD1 accelerated ATM dephosphorylation via WIP1, enhanced DNA repair, reduced senescence and DNA damage, and prevented irradiation-induced hair graying. Elimination of senescent cells with senolytics reduced LSD1 protein in aged organs, indicating a feedback loop between LSD1 and senescence. Mechanistically, LSD1 underwent autophagosome-lysosome degradation through interaction with LC3 and Beclin1, and autophagy impairment during DNA damage contributed to LSD1 accumulation in senescent cells. This study revealed LSD1 as a key regulator of DNA damage–induced senescence and organ aging and suggested that targeting LSD1 may attenuate senescence, delay organ aging, and prevent hair graying.

## Abstract 2

This study demonstrates LSD1 as a key regulator of DNA damage-induced senescence and organ aging and highlights it as a promising therapeutic target.

## Graphical abstract

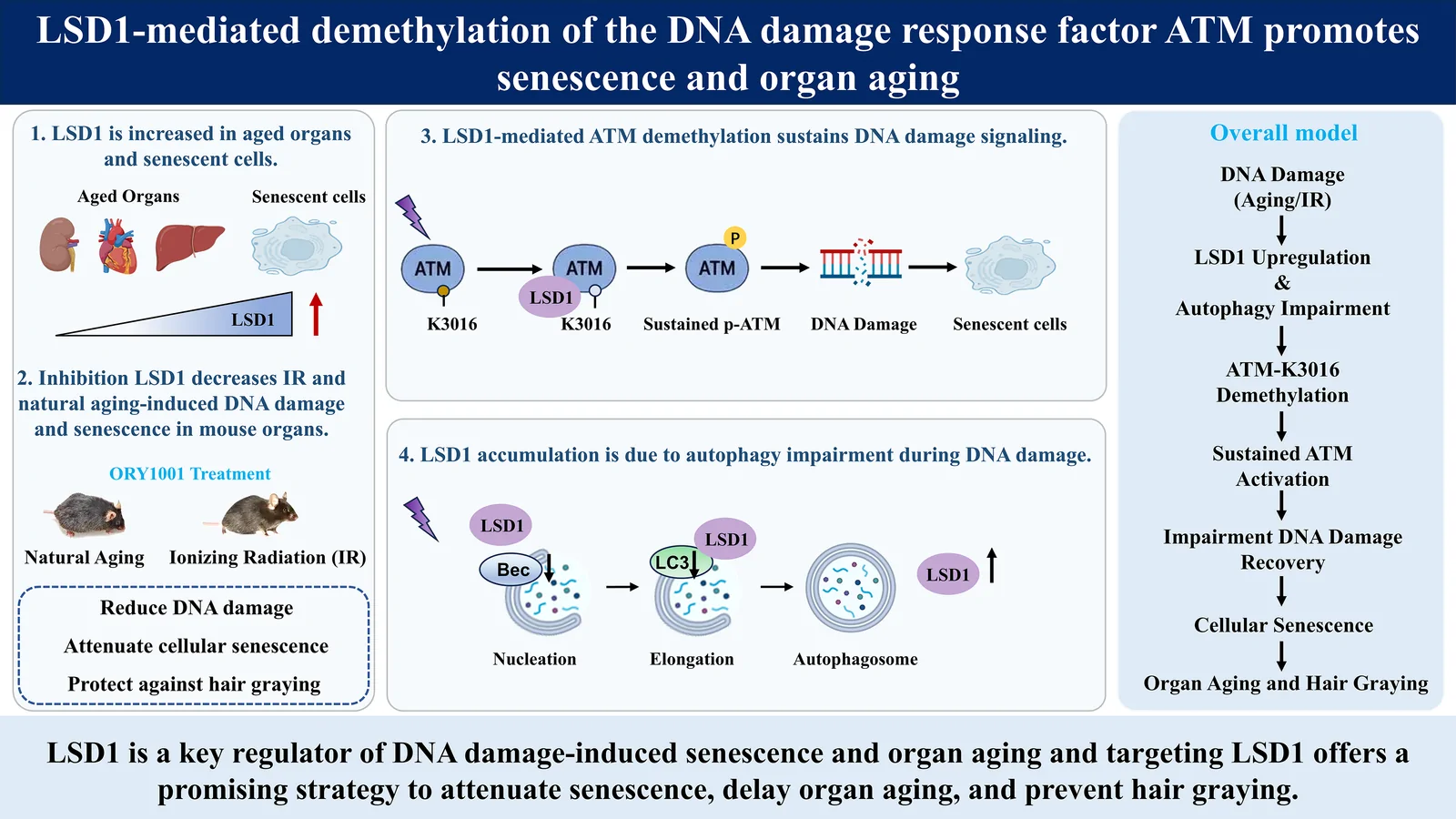

## Introduction

Aging is characterized by a gradual decline of physiological function, such as graying hair, vision and hearing changes, and unnoticeable decline of organ functions (1). Aging process starts much earlier than most people think. The decline starts slowly by the mid-30s and picks up the pace after 65 in women and after 70 for men. Aging is associated with increased risk of diseases, such as cancer, Alzheimer’s disease, diabetes, cardiovascular disease, and many more (2). Currently, aging is assigned to the damage concept, including the accumulation of DNA damage, which may cause biological systems to fail, or to the programmed aging concept, including the internal processes, such as epigenetic maintenance and changes, which may inherently cause aging (3). Although our understanding of the biological mechanisms underlying aging is limited, studies suggest that targeting the aging process itself could potentially ameliorate various age-related pathologies (4).

Cellular senescence is characterized by a state of stable cell cycle arrest (5), which occurs in response to endogenous and exogenous stresses, including telomere dysfunction, oncogene activation, and persistent DNA damage, among others. (6) Acute senescence has beneficial effect, such as tumor suppression, tissue repair and embryogenesis (7), whereas chronic senescence is associated with aging and age-related diseases (8). Eliminating senescent cells has been demonstrated to expand lifespan and delay age-related diseases, supporting a crucial link between cellular senescence and aging (9). A key and fundamental question is how senescence is initiated or caused. This question can be answered with different theories.

For example, DNA damage has been widely recognized as a key cause of cellular senescence (10), which is primarily in the form of DNA double-strand breaks (DSBs), which activate the DNA damage response (DDR) pathway (11). Among the numerous DNA damage–responding factors, ATM (ataxia-telangiectasia, mutated) has been demonstrated to be a crucial protein kinase involved in a wide range of cellular responses, including DNA repair, cell cycle control, and the response to external triggers like radiation (12). ATM-deficient cells derived from patients with ataxia-telangiectasia (AT) are hypersensitive to radiation and show several abnormalities, particularly in the form of defects in response to DNA damage and the G_1_/S, intra-S, and G_2_/M checkpoints (13). In response to DSBs, ATM is rapidly activated and localized to DNA damage sites (14), This activation leads to increased kinase activity and the phosphorylation of numerous downstream targets, including Chk2, H2AX, and p53 (15). These phosphorylation events are crucial for mediating ATM’s effects on DNA repair, cell-cycle arrest, apoptosis, and other processes (16). During DNA damage response and repair, ATM stays at the damage sites for a varying amount of time, from minutes to hours, depending on the nature of the DNA damage lesions (17). It is well known that persistent ATM activation impairs DNA repair and compromises cell survival (18). Conversely, inhibition of ATM activation can enable senescent cells to reenter the cell cycle, suggesting that persistent ATM activation contributes to the stability of the senescent state (19). Thus, DNA damage response must be tightly monitored and regulated to ensure faithful DNA repair and prevent unnecessary cellular alterations (20). Upon DNA damage, ATM is activated by the phosphorylation at S1981 to ensure a transition from an inactive dimer to an active monomer (21). Emerging evidence also shows that this process can be attenuated by other modifications, such as TIP60-mediated acetylation of ATM at lysine 3,016, which is essential for ATM phosphorylation and activation (17). However, whether demethylation of ATM affects its activation remains unknown.

Lysine-specific demethylase 1 (LSD1), the first identified histone/lysine demethylase, has been reported to be upregulated in various diseases (22). LSD1 can demethylate both histone and nonhistone proteins to regulate multiple pathways in cellular processes (23). It has been reported that inhibition of LSD1 induces senescence in Glioblastoma cells through a HIF-1α–dependent pathway (24) and in trophoblast stem cells via induction of Sirt4 (25). In addition, downregulation of LSD1 is involved in cellular senescence through the p53-SUV39h1 axis in CK2-deficient HCT116 and MCF-7 cells (26). All these studies are conducted in different cell types in vitro. However, the role and mechanism of LSD1 in the regulation of organ senescence and aging remain elusive.

In this study, we identify the role of LSD1 in organ senescence and aging and define a mechanistic crosstalk between LSD1 and ATM activation in these processes, as well as the regulation of LSD1 by autophagy. This study supports that targeting LSD1 may represent a novel strategy to mitigate organ senescence and aging and to delay hair graying in aging populations.

## Results

### The expression of LSD1 is increased in aged organs and senescent cells.

**Figure 1:**
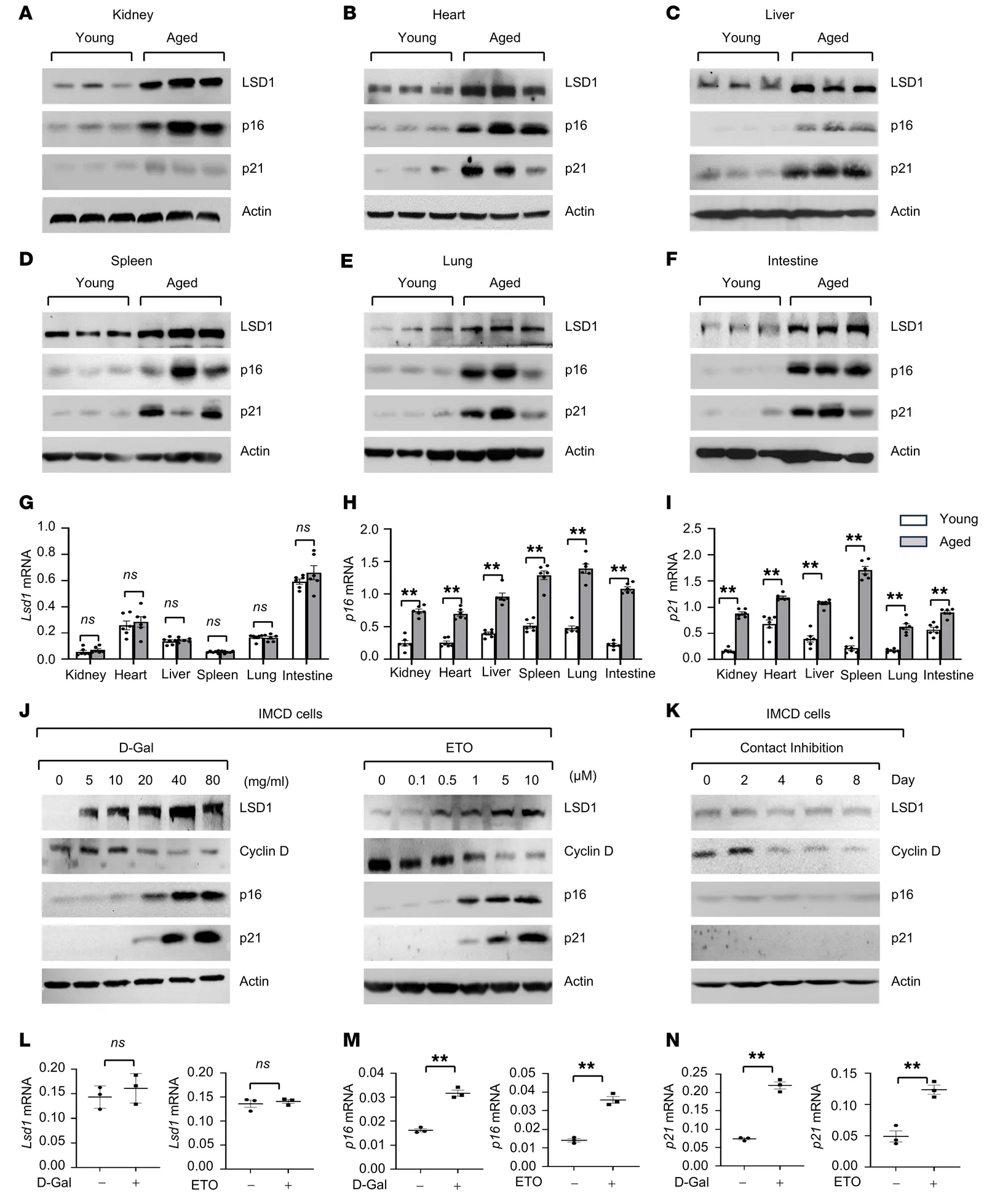
The expression of LSD1 is increased in aged organs and senescent cells. (**A**–**F**) Western blot analysis of the expression of LSD1, p16, and p21 in aged organs, including kidneys (**A**), hearts (**B**), livers (**C**), spleens (**D**), lungs (**E**), and intestines (**F**), collected from 20–22-months-old mice (Aged) compared with expression in young organs collected from 2-month-old mice (Young). *n* = 6. (**G**–**I**) qRT-PCR analysis of *Lsd1* (**G**), *p16* (**H**), and *p21* (**I**) mRNA in mouse organs, including kidney, heart, liver, spleen, lung, and intestine, collected from young and aged mice (*n* = 6, per group; ns, not significant; **P < 0.01; unpaired, 2-tailed Student’s *t* test). (**J**) Western blot analysis of the expression of LSD1, Cyclin D, p16, and p21 from whole-cell lysates of mouse IMCD cells treated with D-galactose (D-Gal) and etoposide (ETO) for 48 hours with different doses. Represent data from 3 independent experiments are shown. (**K**) Western blot analysis of the expression of LSD1, Cyclin D, p16, and p21 in mouse IMCD cells cultured after 100% confluency for indicated days. Represent data from 3 independent experiments are shown. (**L**) qRT-PCR analysis of *Lsd1* (**L**) mRNA in mouse IMCD cells treated with D-Gal (left panel) and ETO (right panel). All experiments were independently repeated at least 3 times; ns, not significant; unpaired, 2-tailed Student’s *t*-test (**M** and **N**) qRT-PCR analysis of *p16* (**M**) and *p21* (**N**) mRNAs in mouse IMCD cells treated with D-Gal or ETO. All experiments were independently repeated at least 3 times; **P < 0.01; unpaired, 2-tailed Student’s *t*-test.

To understand the roles of LSD1 in organ senescence and aging, first, we found that LSD1 protein was increased in aged organs, including kidneys, hearts, livers, spleens, lungs and intestines, collected from 20–22-month-old mice compared with young organs collected from 2-month-old mice (Figure 1, A–F), accompanied by an increase of the expression of p16^ink4a^ (p16) and p21^cip1^(p21), 2 markers of senescence. However, we found that, in contrast with the upregulation of LSD1 protein as well as *Cdkn2A* (*p16*) and *Cdkn1A* (*p21*) mRNA, the expression of *Lsd1* mRNA was not increased in aged organs compared with young organs (Figure 1, G–I).

Cellular senescence plays a crucial role in the progression of organ aging (8). We found that induction of senescence by the oxidative stress injury agent D-Galactose (D-gal) and the DNA damaging agent etoposide (ETO) also increased the expression of LSD1 protein in mouse inner medullary collecting duct (IMCD) cells (Figure 1J) and mouse embryonic fibroblasts (3T3 cells) (Supplemental Figure 1A; supplemental material available online with this article; https://doi.org/10.1172/JCI199854DS1). Treatment with ETO and D-Gal did not affect the expression of *LSD1* mRNA in senescent cells compared with vehicle-treated controls (Figure 1L). The induction of senescence was indicated by the upregulation of p16 and p21 protein and mRNA levels in mouse IMCD cells (Figure 1, J, M, and N). In addition, LSD1 protein was not changed in quiescent cells induced by contact inhibition (Figure 1K). We further found that LSD1 protein was increased in naturally aged primary renal tubular epithelial cells isolated from 20–22-month-old mouse kidneys compared with primary kidney tubular epithelial cells isolated from 2-month-old mouse kidneys (Supplemental Figure 1B). These results support that the stability of LSD1 protein, rather than the regulation of *Lsd1* mRNA, is associated with senescence and aging.

### Targeting LSD1 decreases cellular senescence, and elimination of senescent cells leads to a decline of LSD1 protein in aged organs.

**Figure 2:**
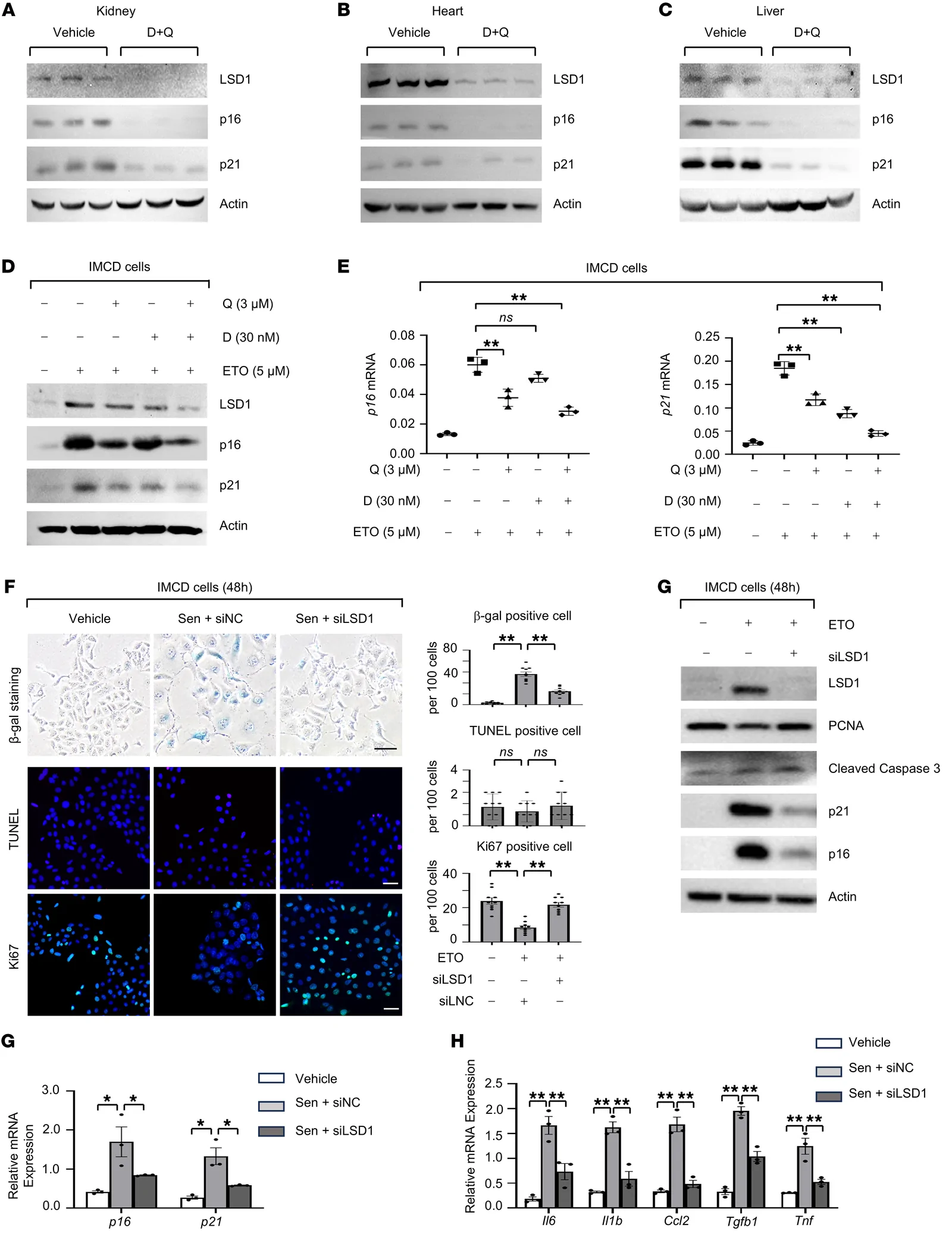
Targeting LSD1 decreases cellular senescence and elimination of senescent cells leads to a decline of LSD1 protein in aged organs. (**A**–**C**) Western blot analysis of the expression LSD1, p16, and p21 in organs, including kidneys (**A**), heart (**B**), and liver (**C**), collected from aged 20–22-month-old mice treated with senolytic drugs (Dasatinib 5 mg/Kg and Quercetin 50 mg/Kg, D + Q). (**D**) Western blot analysis of the expression of LSD1, p16 and p21 in mouse IMCD cells treated with Dasatinib (D) or Quercetin (Q), and cotreated with ETO (5 μM). Represent data from 3 independent experiments are shown. (**E**) qRT-PCR analysis of *p16* and *p21* mRNAs in mouse IMCD cells treated with Dasatinib (D) or Quercetin (Q) and cotreated with ETO (5 μM). All experiments were independently repeated at least 3 times; ns, no significant, **P < 0.01; 1-way ANOVA. (**F**) Representative images and quantification of and SA-β-gal staining (top), TUNEL assay (middle), and Ki67 staining (bottom) in mouse IMCD cells treated with LSD1 siRNA (siLSD1) and control siRNA (siNC) for 48 hours after ETO (5 μM) treatment. Scale bar: 50 μm. The percentage of positive cells was quantified using the same software in 10 randomly selected fields; ns, not significant, **P < 0.01; 1-way ANOVA. (**G**) Western blot analysis of the expression of LSD1, PCNA, cleaved caspase-3, p21, and p16 in mouse IMCD cells treated with ETO (5 μM) and cotreat with or without siLSD1 for 48 hours. *n* = 3. (**H** and **I**) qRT-PCR analysis of *p16* and *p21* (**H**), and SASP factors *Il6*, *Il1b*, *Ccl2*, *Tgfb1*, and *Tnfa* (**I**) mRNAs in mouse IMCD cells with or without LSD1 siRNA, 48 hours after ETO (5 μM) treatment. All experiments were repeated at least 3 times; ns, not significant, *P < 0.05, **P < 0.01; 1-way ANOVA.

To understand the role of LSD1 in organ senescence and aging, we focused on 3 key parenchymal organs: kidney, heart, and liver. We found that treatment with senolytic drugs Dasatinib plus Quercetin (D+Q), which could eliminate senescent cells (27), decreased LSD1 protein in kidneys, hearts, and livers in naturally aged 20–22-month-old mice, accompanied by a reduction of p16 and p21 proteins in those organs (Figure 2, A–C). Administration of Dasatinib (D), Quercetin (Q), or D+Q also decreased the expression of LSD1 protein as well as p16 and p21 in mouse IMCD cells (Figure 2D). In addition, D, Q, or D+Q also decreased the expression of *p16* and *p21* mRNA levels in mouse IMCD cells (Figure 2E). These results suggest that there is a positive feedback loop between the upregulation of LSD1 and cellular senescence in aged organs.

To define the functional role of LSD1 in other cellular process, including proliferation and apoptosis, we knocked down *Lsd1* with siRNA in etoposide-treated (ETO-treated) IMCD cells. ETO treatment not only induced senescence but also reduced proliferation with no effect on apoptosis at 48 hours. Knockdown of *Lsd1* partially restored proliferation and reduced cell senescence, without affecting apoptosis (Figure 2, F and G). Consistent with this, qPCR analysis demonstrated that knockdown or inhibition of LSD1 with ORY-1001 markedly reduced the expression of senescence markers (p16 and p21) and senescence-associated secretory phenotype (SASP) factors, including IL-1β, TNF-α, IL-6, MCP-1, and TGF-β, in senescent IMCD cells (Figure 2, H and I, and Supplemental Figure 2, A and B). LSD1 overexpression further enhanced senescence and suppressed cell proliferation in ETO-treated IMCD cells (Supplemental Figure 3, A–C). Importantly, neither inhibition nor overexpression of LSD1 altered apoptosis in senescent IMCD cells, as assessed by TUNEL assay and the levels of cleaved caspase-3 (Figure 2, F and G, and Supplemental Figure 3, A and B). These results support a model in which upregulation of LSD1 is associated with, and may contribute to, the maintenance of senescence in renal epithelial cells.

### Treatment with ORY-1001 decreases ionizing radiation and natural aging-induced DNA damage and senescence in mouse organs and has a protective effect against IR-induced hair graying.

**Figure 3:**
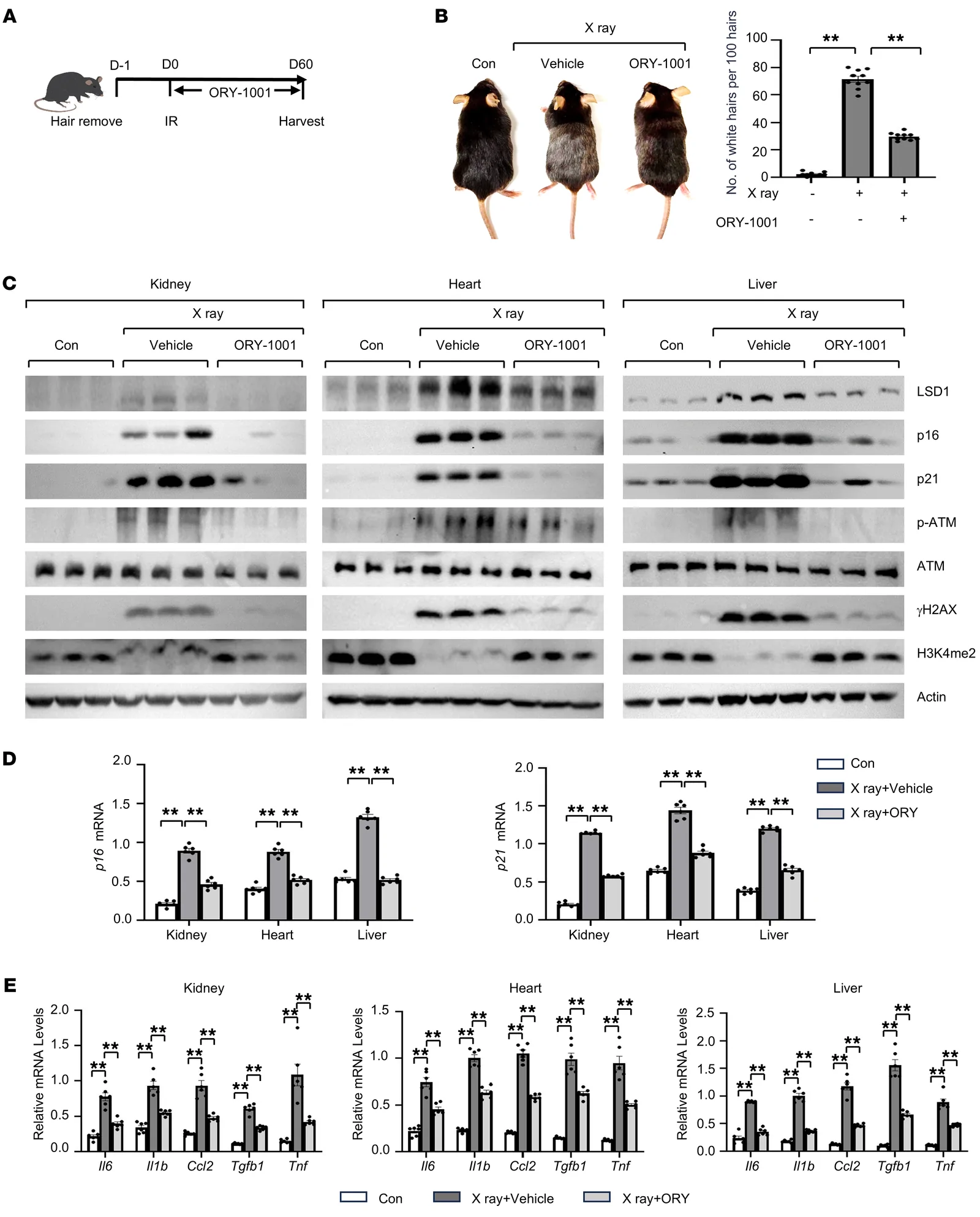
Treatment with ORY-1001 decreases IR-induced DNA damage in mouse organs and protects hair graying. (**A**) Schedule of the irradiation (IR) and ORY-1001 treatment in C57BL/6J mice. (**B**) Representative images of mice from the control group, IR group (7Gy single dose), and IR group plus ORY-1001 (0.04 mg/Kg) injection (60). Error bars represent the mean ± SEM values represent gray hairs numbers per 100 strands. *n* = 10; **P < 0.01; 1-way ANOVA. (**C**) Western blot analysis of the levels of LSD1, p16, p21, phospho-ATM (p-ATM), total of ATM, and γH2AX in mouse organs, including kidneys (left panel), heart (middle panel), and livers (right panel), collected from the mice treated with vehicle, IR, and IR plus ORY-1001. (**D–E**) qRT-PCR analysis of *p16/p21* (**D**), and inflammatory/SASP factors *Il6*, *Il1b*, *Ccl2*, *Tgfb1*, and *Tnfa*. (**E**) mRNA levels in mouse organs. *n* = 6/group; **P < 0.01; 1-way ANOVA.

Ionizing radiation (IR) generates a high level of DNA damage, which often leads to the induction of cellular senescence and multiorgan aging (28). To determine the functional role of LSD1 in these processes, 10-week-old C57BL/6J mice were subjected to a single 7 Gy radiation dose, and then treated with LSD1 inhibitor ORY-1001 (0.04 mg/kg) or PBS by intraperitoneal injections every other day for a duration of 8 weeks (Figure 3A). IR exposure induced hair graying, which was attenuated by ORY-1001 treatment (Figure 3B). IR also increased LSD1 expression in kidney, heart, and liver, accompanied by elevated p16 and p21 levels. Notably, ORY-1001 treatment reduced both mRNA and protein levels of p16 and p21 in these tissues (Figure 3, C and D), indicating attenuation of IR-associated senescence markers. Additionally, ORY-1001 treatment reduced the levels of SASP factors, including IL-1β, TNF-α, IL-6, MCP-1, and TGF-β, in IR-treated organs (Figure 3E).

To assess the impact of LSD1 inhibition on organismal aging, 22-month-old mice were treated with the LSD1 inhibitor ORY-1001 (0.04 mg/kg) and PBS via intraperitoneal injection every other day until 28 months of age (Supplemental Figure 4A). ORY-1001–treated mice exhibited protection against hair graying compared with controls (Supplemental Figure 4B) and markedly attenuated senescence, as seen by decreased senescence-associated β-galactosidase (SA-β-gal) staining, reduced expression of p16 and p21, and suppression of SASP factors (TGF-β, IL-1β, TNF-α, IL-6, and MCP-1), as well as attenuated fibrosis in aged kidney, heart, and liver tissues (Supplemental Figure 4, C–G). These results provide in vivo evidence that LSD1 promotes age-associated tissue dysfunction and that its inhibition mitigates senescence and aging phenotypes.

### LSD1-mediated regulation of senescence is independent of canonical histone methylation at p16/p21 loci.

LSD1 is known to demethylate dimethylated H3 lysine residues (e.g., H3K4me2 or H3K9me2) to monomethylated forms (29, 30). Consistent with this activity, aged tissues (kidney, heart, and liver) and senescent cells exhibited decreased H3K4me2 and H3K9me2, accompanied by increased H3K4me1 and H3K9me1 (Supplemental Figure 5, A and B). H3K4me1 is generally associated with transcriptional activation, whereas H3K9me2 is linked to transcriptional repression. Given these changes and the increased expression of LSD1 in naturally aged and senescent conditions, we investigated the potential involvement of H3K4me1 and H3K9me2 in regulating the transcription of *p16* and *p21*. Analysis of publicly available ChIP-seq databases (GSE106415) showed the binding of H3K4me1 on the promoter of *p21* and *p16* (Supplemental Figure 5, C and D). However, Analysis of ChIP-seq databases (GSE38442) revealed no significant changes in the occupancy or peak enrichment of H3K9me2 at these loci under senescent conditions (Supplemental Figure 5E). These results suggest that LSD1 promotes cellular senescence, at least in part, through canonical histone methylation at the *p16*/*p21* loci.

### LSD1 is involved in DNA damage–mediated senescence and DNA damage repair.

ATM is a central kinase in the DNA damage response (DDR) to double-strand breaks (31). Upon DNA damage, ATM is rapidly phosphorylated and recruited to sites of DNA lesions, where it remains active for minutes to hours before undergoing progressive dephosphorylation as repair is completed (32). Given that cellular senescence is frequently driven by DNA damage, we investigated whether LSD1 is functionally linked to DNA damage response pathways. We found that increased LSD1 protein was associated with elevated ATM phosphorylation in IR-treated tissues and aged (28-month-old) organs, whereas treatment with the LSD1 inhibitor ORY-1001 reduced levels of phosphorylated ATM and γH2AX in kidney, heart, and liver under both conditions (Figure 3C and Supplemental Figure 4F).

**Figure 4:**
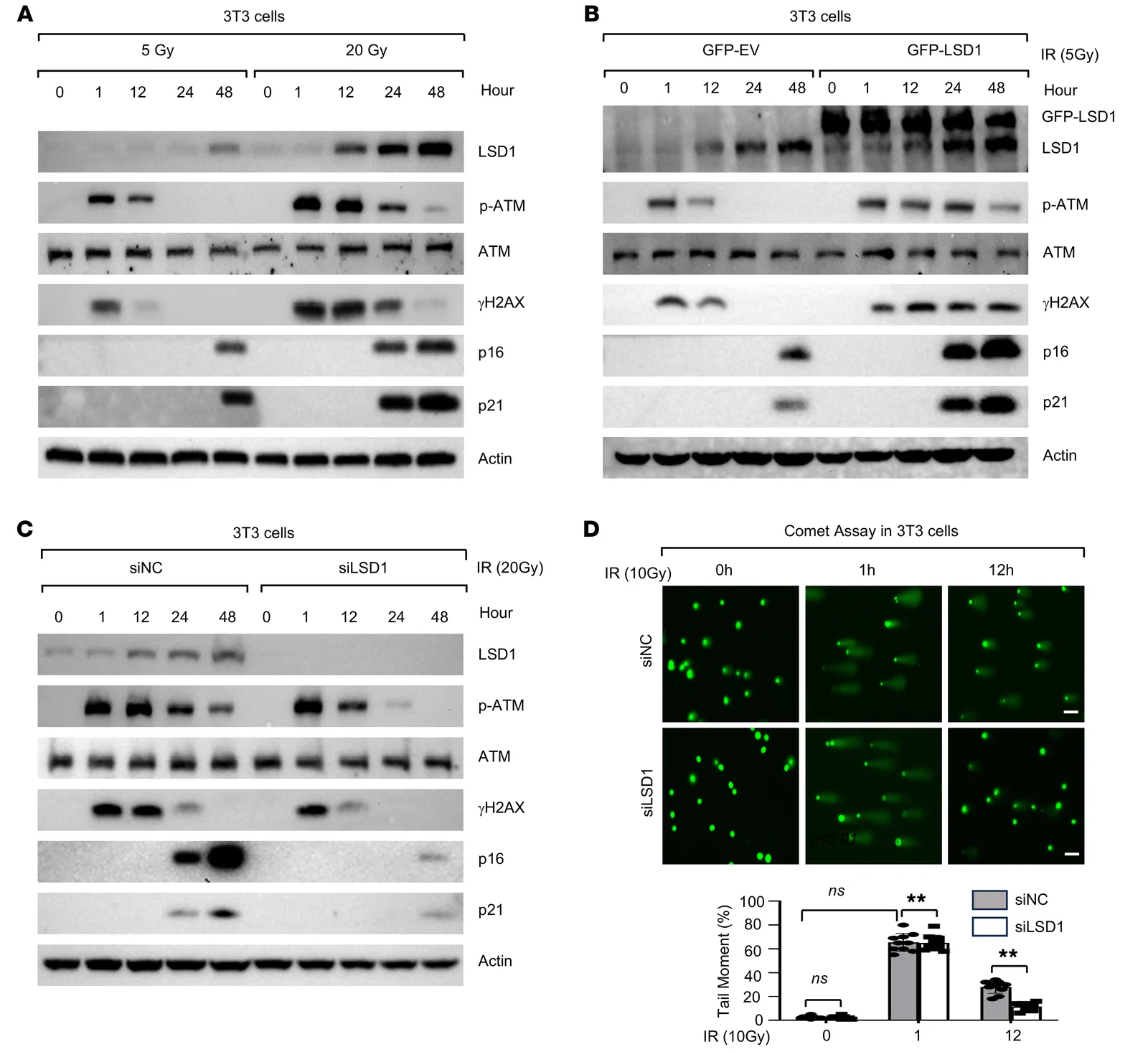
Increased LSD1 is involved in DNA damage–mediated senescence and DNA damage repair. (**A**) Western blot analysis of the levels of LSD1, phospho-ATM, and total of ATM, γH2AX. p16 and p21 in mouse 3T3 cells treated with either 5Gy- IR or 20Gy-IR at indicated time points. (**B**) Western blot analysis of the levels of GFP-LSD1, LSD1, phospho-ATM, and total of ATM, γH2AX, p16, and p21 in GFP-LSD1 and GFP-empty vector transfected 3T3 cells treated with IR (5 Gy) at indicated time points. (**C**) Western blot analysis of the levels of LSD1, phospho-ATM and total of ATM, γH2AX, p16, and p21 in *Lsd1* knockdown (siLSD1) 3T3 cells treated with IR (20 Gy) compared with the control siRNA (siNC) at indicated time points. Representative data from 3 independent experiments are shown. (**D**) Representative images and statistical graph of comet assay in *Lsd1* knockdown (siLSD1) 3T3 cells treated with IR at indicated time points. Error bars represent the SEM. Data were analyzed from 10 images in 3 independent experiments. Scale bar: 50 μm. ns, no significance, *P < 0.05, **P < 0.01; 1-way ANOVA.

To investigate the relationship between LSD1 and ATM following DNA damage, we examined whether LSD1 regulates ATM activation dynamics in IR-induced DDR and senescence. 3T3 cells were exposed to different doses of IR and analyzed over time. Low-dose IR (5 Gy) did not alter LSD1 expression until 48 hours after treatment, although it induced rapid ATM phosphorylation and γH2AX accumulation at 1 hour, which decreased to baseline by 12 hours. In contrast, high-dose IR (20 Gy) increased LSD1 expression at approximately 12 hours after irradiation and was associated with prolonged ATM phosphorylation and delayed resolution of γH2AX, persisting up to 24 hours (Figure 4A). Consistently, overexpression of GFP-tagged LSD1 under low-dose IR (5 Gy) prolonged ATM phosphorylation and delayed γH2AX resolution up to 48 hours, accompanied by increased p16 and p21 expression at 24 hours compared with controls (Figure 4B). Conversely, LSD1 knockdown under high-dose IR (20 Gy) accelerated ATM dephosphorylation and γH2AX clearance and reduced p16 and p21 expression (Figure 4C). Moreover, LSD1 knockdown enhanced DNA repair efficiency, as assessed by comet assay at 12 hours after IR (Figure 4D). These results indicate that LSD1 modulates the duration, rather than the initiation, of ATM signaling. While ATM phosphorylation is rapidly induced following DNA damage, LSD1 appears to contribute to the maintenance and amplification of ATM signaling, which is associated with sustained DNA damage response (DDR) activation and senescence. These results also provide insight into mechanistic prolonged ATM activation and suggest a role for LSD1 in limiting “checkpoint recovery” following genotoxic stress.

### LSD1 interacts with and demethylates ATM.

**Figure 5:**
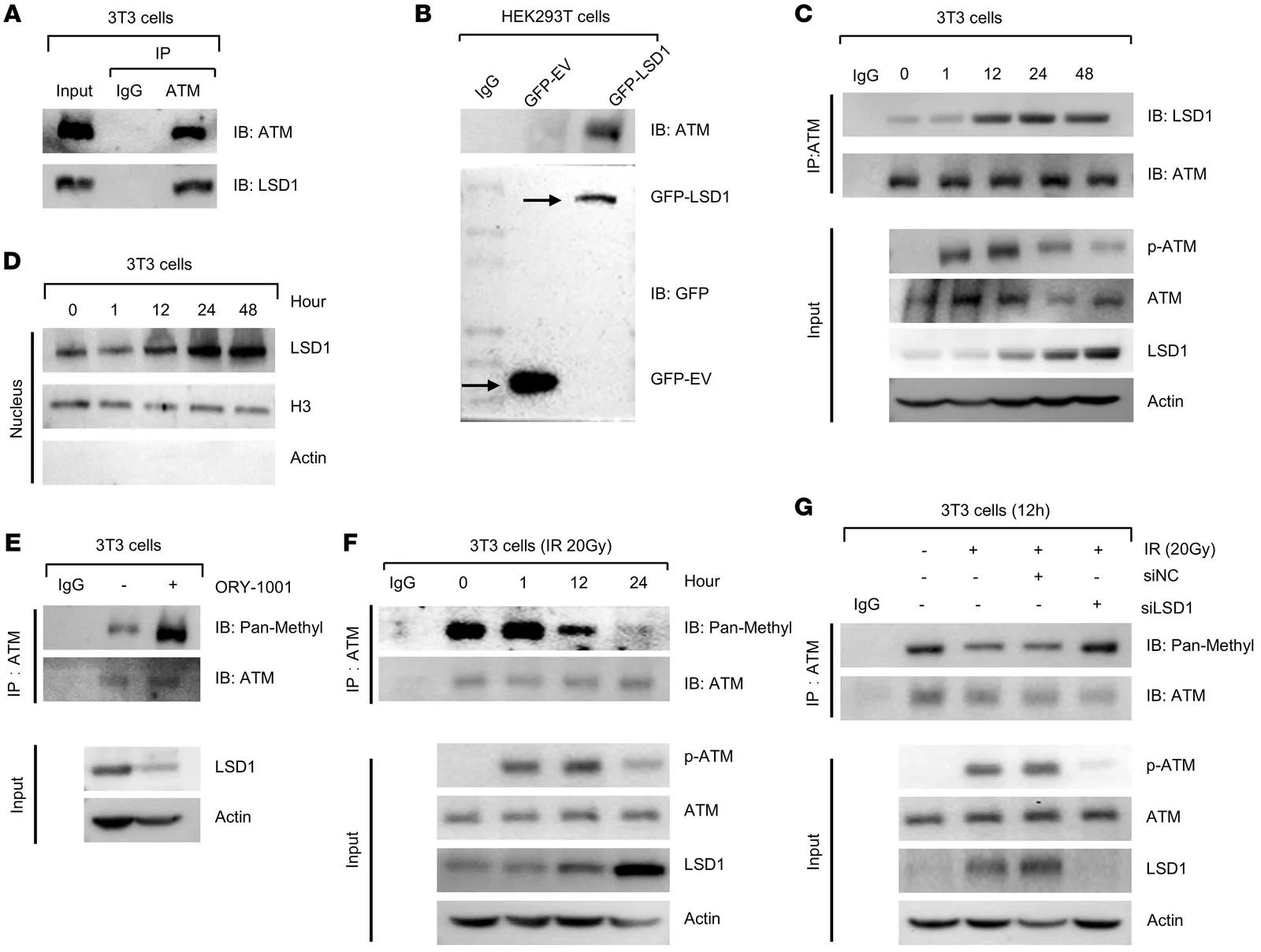
LSD1 interacts with and demethylates ATM. (**A**) Immunoprecipitation assay to detect the interaction between ATM and LSD1 in mouse 3T3 cells. Immunoglobulin G (IgG) was used as a negative control. (**B**) Immunoprecipitation assay to detect the interaction between GFP tagged LSD1 and ATM in HEK 293T cells transfected with GFP empty vector (EV) and GFP tagged LSD1 construct (GFP-LSD1). (**C**) Immunoprecipitation assay to detect the interactions between ATM and LSD1 in mouse 3T3 cells treated with IR (10 Gy) at indicated time points. (**D**) The nuclear fractions were isolated from mouse 3T3 cells treated with IR (10Gy) at indicated time points. Western blot analysis of the LSD1 expression in nuclear fractions. (**E**) Immunoprecipitation assay to detect the methylation of ATM in mouse 3T3 cells treated with ORY-1001 (5 μM) or vehicle. (**F**) Immunoprecipitation assay to detect the methylation of ATM in mouse 3T3 cells treated with IR (20 Gy) at indicated time points. (**G**) Immunoprecipitation assay to detect the methylation of ATM in mouse 3T3 cells treated with LSD1 siRNA or control siRNA (siNC) at 12 hours after IR (20 Gy) treatment.

To investigate how LSD1 regulates DNA damage repair, we found an interaction between LSD1 and ATM in 3T3 cells as examined with coimmunoprecipitation (co-IP) assay (Figure 5A). In addition, we found that GFP-tagged LSD1 could also pull-down ATM in HEK293T cells, as examined with anti-GFP antibody (Figure 5B). Notably, this interaction was markedly enhanced upon induction of DNA damage, particularly at 12 hours after IR, as demonstrated by co-IP assays at different time points of IR treatment in 3T3 cells (Figure 5C). We further found that the upregulation of LSD1 following IR treatment is accompanied by an increase of nuclear LSD1 where its interaction with ATM was also enhanced, as examined by nucleus separation assays (Figure 5D).

Modification of ATM has been demonstrated to be essential for its activation upon DNA damage, such as its acetylation and phosphorylation (17). However, whether the methylation of ATM affects its activation remains unknown. Based on the interaction of LSD1 with ATM, we examined whether LSD1 affects the methylation levels of ATM in 3T3 cells by using anti-ATM antibody for immunoprecipitation, followed by blotting with antimethyl lysine antibody. We found that inhibition of LSD1 with ORY-1001 increased the methylation of ATM in 3T3 cells (Figure 5E). To explore the dynamic regulation of ATM methylation, we examined changes in ATM methylation in response to DNA damage. We observed that ATM methylation progressively decreased by 12 hours following IR (20 Gy) treatment, coinciding with increased LSD1 expression in 3T3 cells (Figure 5F), indicating that LSD1 contributes to ATM demethylation at this stage. Notably, depletion of LSD1 with siRNA prevented this decrease in methylation of ATM at 12 hours after IR (20 Gy) treatment, suggesting that LSD1 is responsible for the demethylation of ATM at 12 hours after IR (Figure 5G). Our results suggest that LSD1-mediated demethylation of ATM is associated with sustained ATM phosphorylation and occurs predominantly during the later stages of DNA damage response.

### LSD1 demethylates ATM at lysine 3,016 (K3016).

**Figure 6:**
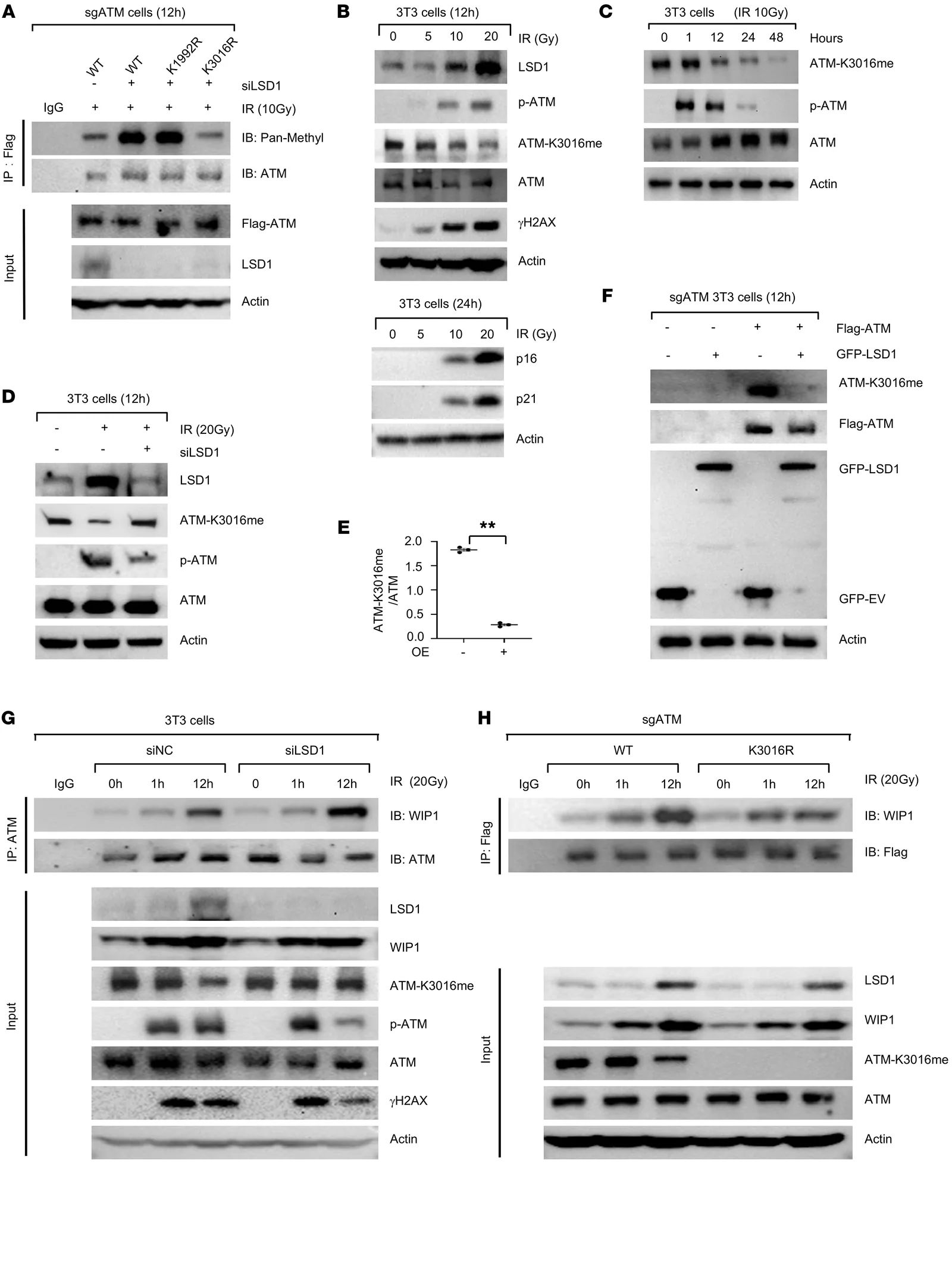
LSD1 demethylates ATM at K3016, and LSD1 mediated demethylation of ATM inhibits its interaction with WIP1. (**A**) Immunoprecipitation assay to detect the methylation of ATM in the indicated plasmids transfected ATM knockout 3T3 cells treated with LSD1 siRNA or control siRNA (siNC) at 12 hours post IR (10 Gy). (**B**) Western blot analysis of LSD1, ATM-K3016 methylation (ATM-K3016me), phospho-ATM (p-ATM), and total ATM at 12 hours after irradiation (IR), as well as p16 and p21 at 24 hours after IR, in 3T3 cells treated with the indicated irradiation doses. (**C**) Western blot analysis of the levels of ATM-K3016 methylation (ATM-K3016me), p-ATM, and total ATM in 3T3 cells treated with 10 Gy and harvest cells at indicated time points. (**D**) Western blot analysis of the levels of LSD1, methylated ATM at K3016, phospho-ATM, and total of ATM in 3T3 cells treated with LSD1 siRNA and control siRNA (siNC) at 12 hours after IR (10 Gy). (**E** and **F**) Western blot and statistical analysis of the levels of methylated ATM at K3016, Flag-tagged ATM, and GFP-tagged LSD1 in ATM knockout mouse 3T3 cells transfected with the indicated plasmids. All experiments were repeated at least 3 times; **P < 0.01; unpaired, 2-tailed Student’s *t* test. (**G**) Immunoprecipitation assay to detect the interaction between ATM and WIP1 in mouse 3T3 cells treated with LSD1 siRNA or control siRNA (siNC) at the indicated time points after IR (20 Gy). (**H**) Immunoprecipitation assay to detect the interaction between Flag-tagged ATM and WIP1 in ATM knockout 3T3 cells transfected with the indicated plasmids at indicated time points after 20 Gy irradiation injury.

The lysines of ATM at K1992 and K3016 have been associated with ATM phosphorylation in previous studies, in that the serine site at S1981 of ATM that is close to K1992 can be phosphorylated following DNA damage and the acetylation of K3016 is a prerequisite for the phosphorylation of ATM at S1981 (33). To determine whether LSD1 demethylates ATM at K1992 and K3016 sites, we generated 2 mutant ATM constructs by replacing these two lysine (K) sites with arginine (R), respectively. We also generated ATM knockout (KO) 3T3 cells using CRISPR-Cas9 technology (Supplemental Figure 6, A and B). We transfected flag-tagged WT ATM and its lysine-to-arginine mutants, including ATM-K1992R and ATM-K3016R, into ATM-KO 3T3 cells. We found that inhibition of LSD1 recovered the methylation of WT ATM and mutant ATM-K1992R, but not methylation in mutant ATM-K3016R in ATM-KO 3T3 cells at 12 hours after IR exposure (Figure 6A). To further demonstrate whether ATM is methylated at K3016 by LSD1, we generated a polyclonal antibody against methylated K3016 peptide of ATM, ATM-K3016me. The specificity of ATM-K3016me antibody was characterized by ELISA and the site-directed mutagenesis (Supplemental Figure 7; see details in Methods). These results confirmed that the newly generated antibody specifically recognizes methylation at ATM-K3016.

To define the relationship between ATM phosphorylation and ATM-K3016 methylation following DNA damage, we performed dose- and time-dependent analyses. At 12 hours after IR, LSD1 expression increased in parallel with a dose-dependent reduction in ATM-K3016 methylation (Figure 6B). Notably, during the early phase (1 hour) after DNA damage, ATM phosphorylation was already detectable, whereas ATM-K3016 methylation remained unchanged (Figures 6C). In addition, we found that knockdown of LSD1 with siRNA reversed IR-induced ATM-K3016 demethylation and reduced ATM phosphorylation at 12 hours after IR (20 Gy) in 3T3 cells (Figure 6D). Conversely, overexpression of GFP-tagged LSD1 decreased ATM methylation in ATM-deficient (KO) 3T3 and mouse IMCD cells reconstituted with Flag-ATM (Figure 6, E and F, and Supplemental Figure 6C). Our results demonstrate that LSD1 demethylates ATM at K3016, thereby modulating the phosphorylation of ATM following DNA damage.

### LSD1-mediated demethylation of ATM inhibits its interaction with WIP1.

It has been reported that WIP1 (WT p53-induced phosphatase 1) can dephosphorylate ATM to attenuate DNA damage–induced stress response (34). We found that ATM interacted with WIP1 at late stages of DNA damage repair in 3T3 cells (Figure 6G), which led to ATM dephosphorylation and deactivation once DNA repair was completed (35). In LSD1-knockdown 3T3 cells, this interaction was markedly increased compared with control cells at 12 hours after IR exposure (Figure 6G). To investigate whether the methylation of ATM at lysine 3,016 affects its interaction with WIP1, we reintroduced either flag-tagged WT and K3016R mutant ATM into ATM-stable knockout 3T3 cells. We found that the interaction of flag-tagged WT ATM with WIP1 could be detected as early as 1 hour and was increased at 12 hours after IR treatment, whereas the interaction of flag-tagged mutant ATM-K3016R with WIP1 could be detected at 1 hour but was decreased at 12 hours after IR treatment in ATM-KO 3T3 cells (Figure 6H).

Previous studies have shown that acetylation at ATM-K3016 regulates ATM activation (36). To compare the roles of acetylation and methylation at this site, we generated an acetylation-mimetic mutant (K3016Q). At 12 hours after IR, K3016Q mutant showed modestly increased ATM phosphorylation and reduced interaction with WIP1 compared with the methylation-deficient K3016R mutant (Supplemental Figure 8A). Consistent with prior reports, acetylation at K3016 predominates during the early phase of the DNA damage response — 10 minutes to approximately 8 hours post-IR (17) — whereas our results indicate that methylation at this site becomes more prominent at later stages (approximately 12 hours) (Figure 6C). To further define this temporal switch, we enhanced acetylation using nicotinamide (NAM) or inhibited LSD1-mediated demethylation using ORY-1001 at 8 hours after IR for 4 hours. Enhancing acetylation at this stage had minimal effect on ATM phosphorylation, whereas inhibition of demethylation markedly reduced ATM phosphorylation (Supplemental Figure 8B). These results support a model in which LSD1-mediated demethylation of ATM at K3016 sustained ATM phosphorylation during the late phase of the DNA damage response, at least in part by limiting its interaction with WIP1, which further suggests a role for LSD1 in prolonging ATM signaling following genotoxic stress.

### LSD1 is subjected to autophagosome-lysosome degradation during organ senescence and aging.

**Figure 7:**
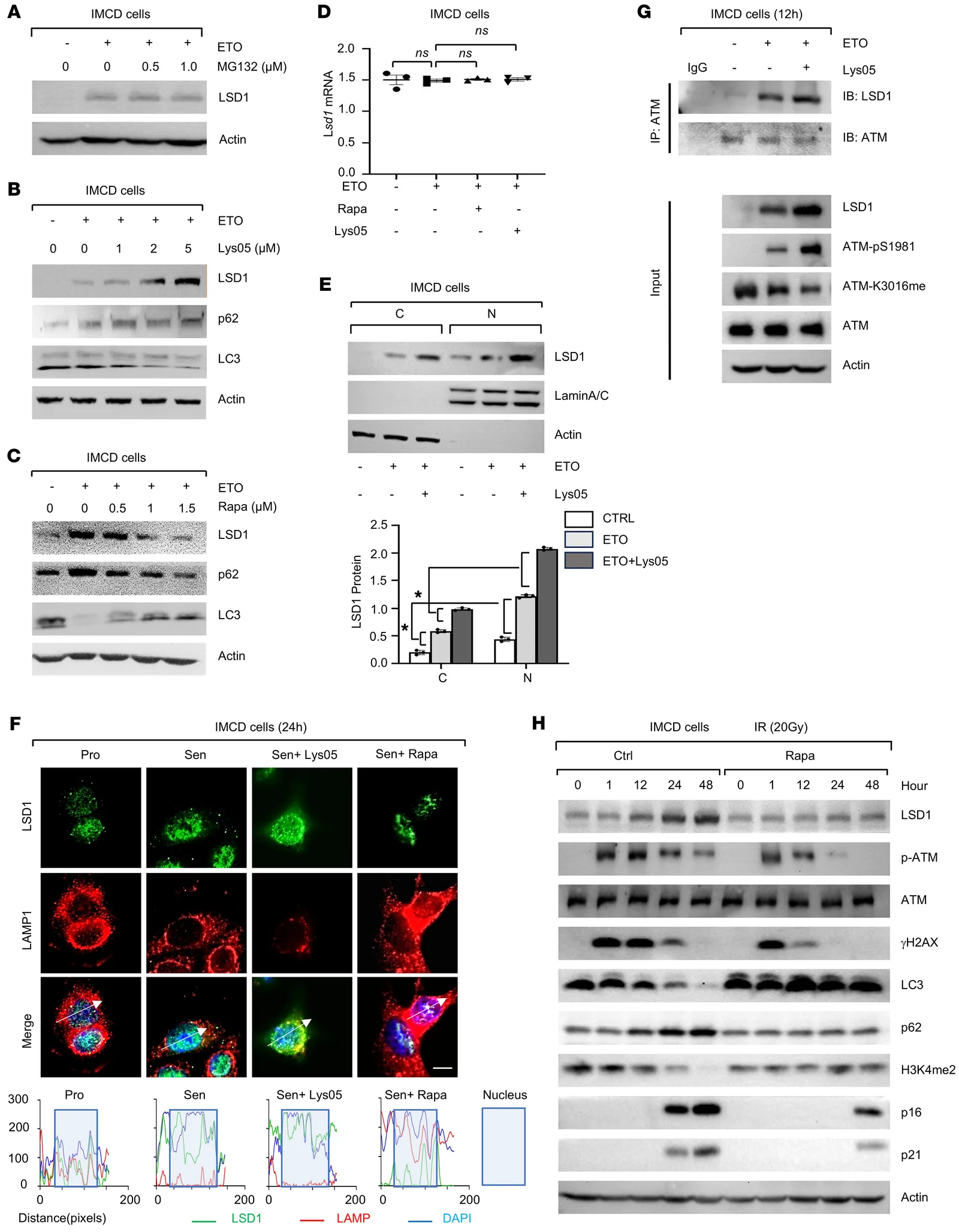
LSD1 is subjected to autophagosome-lysosome degradation during cellular senescence and organ aging. (**A**) Western blot analysis of the expression of LSD1 in mouse IMCD cells treated with ETO and MG132 with indicated doses. (**B** and **C**) Western blot analysis of the expression of LSD1 in mouse IMCD cells treated with ETO, and cotreated with Lys05 (**B**) or Rapamycin (**C**) with indicated doses. (**D**) qRT-PCR analysis of *Lsd1* mRNA in mouse IMCD cells treated with ETO and cotreated with Lys05 or Rapamycin for 48 hours. All experiments were repeated at least 3 times; ns, not significant; 1-way ANOVA. (**E**) Western blot analysis of the expression of LSD1 in nuclear (N) and cytoplasmic (C) fractions isolated from mouse IMCD cells treated with ETO and then Lys05 (5 μM). All experiments were repeated at least 3 times; *P < 0.05; 1-way ANOVA. (**F**) Representative image and quantification of the immunofluorescence staining for LSD1 (green) and LAMP1 (red) in mouse ETO-induced IMCD cells treated with lys05 (5 μM) or rapamycin (1.5 μM). Scale bar: 25 μm. (**G**) Immunoprecipitation assay to detect the interaction between ATM and LSD1 in mouse IMCD cells treated with or without Lys05. (**H**) Western blot analysis of the levels of LSD1, phospho-ATM (p-ATM) and total ATM, p62, LC3B, H3K4me2, p16, and p21 in IR-induced mouse IMCD cells treated with rapamycin or vehicle at the indicated time points. ns, no significance, *P < 0.05.

Next, we investigate the mechanism of how LSD1 protein is increased during organ senescence and aging. We found that treatment with the proteasome inhibitor MG132 had no effect on the levels of LSD1 protein in senescent mouse IMCD cells (Figure 7A). We then tested whether lysosome-mediated degradation contributes to LSD1 protein upregulation. A central lysosomal-mediated process is autophagy, which is inhibited upon cellular senescence (37). We found that treatment with autophagy inhibitor Lys05 (a dimeric form of chloroquine) increased LSD1 protein in senescent mouse IMCD cells in a dose-dependent manner (Figure 7B), accompanying a decrease of the expression of LC3 and an increase of the expression of p62, a substrate of autophagy, whereas treatment with one of the autophagy inducers, rapamycin (Rapa), decreased LSD1 protein in senescent mouse IMCD cells in a dose-dependent manner, accompanying an increase of the expression of LC3 and a decrease of the expression of p62 (Figure 7C). Treatment with Lys05 or Rapa did not affect the levels of *Lsd1* mRNA in mouse IMCD cells treated with ETO, as examined by qRT-PCR analysis (Figure 7D), indicating posttranslational regulation.

Nuclear-cytoplasmic fractionation revealed that LSD1 is predominantly localized in the nucleus in senescent cells, with autophagy inhibition further enhancing its nuclear accumulation (Figure 7E). Immunofluorescence staining confirmed this dynamic regulation, showing increased nuclear LSD1 upon autophagy inhibition and reduced nuclear localization upon autophagy activation (Figure 7F). Coimmunoprecipitation experiments demonstrated that inhibition of autophagy sustained the interaction between LSD1 and ATM following DNA damage, accompanied by decreased ATM-K3016 methylation and prolonged ATM phosphorylation at 12 hours after IR (Figure 7G). Conversely, activation of autophagy with rapamycin reduced LSD1 protein levels in IR-treated IMCD cells, along with decreased ATM phosphorylation, γH2AX and senescence markers (p16 and p21), as well as reduced H3K4me2 (Figure 7H).

Importantly, upregulation of LSD1 was associated with a decrease of autophagy in aged organs, including kidney, heart, and liver, characterized by a decrease of the expression of LC3 but an increase of the expression of p62 (Supplemental Figure 9A). Pharmacological inhibition of autophagy with Lys05 increased LSD1 protein in young organs, including kidneys, hearts, and livers, compared with that in age-matched organs treated with vehicle, in which the inhibition of autophagy was confirmed by the accumulation of p62 in those organs (Supplemental Figure 9B). Notably, *Lsd1* mRNA levels remained unchanged following Lys05 treatment in those organs (Supplemental Figure 9C). Activation of autophagy with rapamycin decreased LSD1 protein without affecting its mRNA in aged organs from 20–22-month-old mice compared with age-matched vehicle treated controls (Supplemental Figure 9, D and E). In sum, these results indicate that LSD1 is regulated posttranscriptionally by autophagy and support a dynamic interplay among DNA damage, autophagy, and LSD1-mediated regulation of cellular senescence both in vitro and in vivo.

### LSD1 interacts with autophagy proteins LC3 and Beclin1.

**Figure 8:**
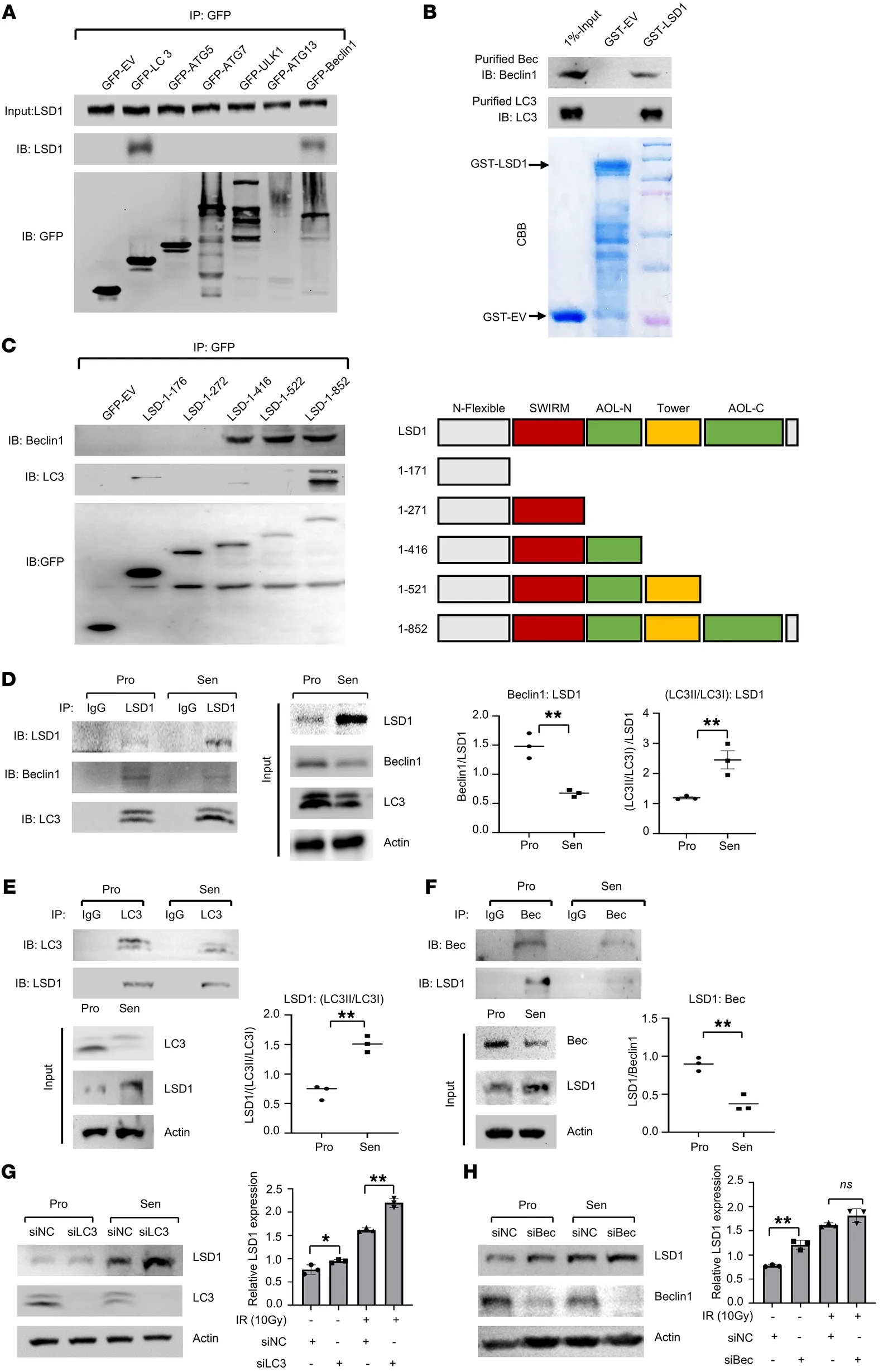
LSD1 interacts with autophagy protein LC3 and Beclin1. (**A**) Immunoprecipitation assay to detect the interaction of LSD1 with GFP-tagged constructs, including LC3, ATG5, ATG7, ULK1, ATG13, and Beclin1. (**B**) GST pull-down assay of bacteria-expressed LSD1 with recombination LC3 and Beclin1 protein. (**C**) Immunoprecipitation assay to detect the interaction of GFP-tagged LSD1 truncated constructs with LC3 or Beclin1. (**D**–**F**) Immunoprecipitation assay to detect the interactions between LSD1 and LC3 or Beclin1 in mouse IMCD cells treated with ETO or vehicle. Statistical analysis of the 3 independent expressions of IP/IB is shown in **D**–**F**. **P < 0.01; unpaired, 2-tailed Student’s *t* test. (**G** and **H**) Western blot analysis and quantitative data of the expression of LSD1 in mouse IMCD cells treated with LC3 siRNA (**G**) or Beclin1 siRNA (**H**) or control siRNA (siNC) at 48 hours. Statistical analysis of the 3 independent expression; ns, not significant, *P < 0.05, **P < 0.01; 1-way ANOVA.

Binding to autophagy proteins is essential for the degradation of autophagy substrates (37). To determine whether LSD1 interacts with autophagy component(s), we transfected GFP-tagged constructs of autophagy components, including LC3, ATG5, ATG7, ATG13, Beclin1, and ULK1, into HEK293T cells and performed immunoprecipitation assay. The expression of GFP-tagged autophagy proteins was detected with Western blot analysis. We found that only GFP-tagged LC3 and Beclin1 could pull down LSD1 (Figure 8A). In addition, GST pull down using bacteria-expressed and purified GST-LSD1 and LC3/Beclin1 proteins showed that LSD1 directly binds to LC3 and Beclin1 (Figure 8B).

Next, we defined the region within LSD1 that interacted with Beclin1 and LC3 by generating GFP-tagged LSD1 subdomain deletion constructs. The GFP pull-down assay showed that Beclin1 interacts with the AOL-N domain of LSD1, and LC3B interacts with the AOL-C domain of LSD1 (Figure 8C). The AOL domain has been defined as the catalytic center and is responsible for targeting substrate proteins (38). These results suggest that the dysregulation of LSD1 by autophagy is possibly regulated through the interaction of the AOL domain of LSD1 with Beclin1 and LC3.

### LC3 is responsible for the lysosomal degradation of LSD1 in senescent cells.

Our above results indicated that LSD1 interacted with LC3 and Beclin1 at the proliferating state, and we next investigated whether induction of senescence affected their interaction by performing co-IP assay with anti-LSD1, anti-LC3, and anti-Beclin1 antibodies. We found that the interaction of LSD1 with LC3 was at a similar level in proliferating and senescent mouse IMCD cells, whereas the interaction of LSD1 with Beclin1 was decreased in senescent mouse IMCD cells compared with the interaction in proliferating mouse IMCD cells (Figure 8, D–F). We further found that knockdown of Beclin1 could enhance the expression of LSD1 in proliferating cells but not expression in senescent cells, whereas knockdown of LC3 could augment the expression of LSD1 in both proliferating and senescent cells (Figure 8, G and H). These results suggested that,in the context of cellular proliferation and senescence, the degradation of LSD1 is regulated by distinct autophagy-related proteins, Beclin1 and LC3.

To further investigate the interaction of LSD1 with either Beclin1 or LC3, we assessed the affinity by performing the molecular docking experiments and molecular dynamic simulation assay (39). The Root Mean Square Deviation (RMSD) value of the protein backbone of LSD1 and LC3, as well as LSD1 and Beclin1, was calculated to determine the stability of protein structure during the period of simulation by protein docking test. We found an instability of the interaction of LSD1 and Beclin1 as well as LSD1 and LC3B in the initial 20 nanosecond (ns) of the simulation, whereas a stable interaction was observed from 20–50 nanoseconds by dynamics simulation. The plots of LSD1 and Beclin1 as well as LSD1 and LC3B exhibit average RMSD values of 18 Å and 15 Å, respectively (Supplemental Figure 10, A and B). We also utilized AlphaFoldmultimer (40) to predict the 3D structure and interaction between LSD1 and Beclin1 as well as LSD1 and LC3. The red box represents the predicted Aligned error (pAE) of protein-protein interactions. We found that the value of pAE, which was indicated in the red boxes, of LSD1 with LC3B is lower than that of LSD1 with Beclin1 (Supplemental Figure 10C). During the entire 50 nanoseconds of molecular dynamics (MD) simulation, LSD1 formed a maximum of 65 hydrogen bonds with Beclin1, while LSD1 formed a maximum of 700 hydrogen bonds with LC3, as shown by the frequency plots (Supplemental Figure 10D). These findings suggest that the interaction between LSD1 and LC3 exhibits a higher binding affinity than Beclin1. Collectively, our results suggest that LSD1 interacts with Beclin1/LC3 at the basal state. However, due to the reduced expression of Beclin1 during cellular senescence, LSD1 cannot be fully cleared. Meanwhile, LC3 exhibits enhanced binding affinity with LSD1 compared with Beclin1, supporting that LC3 is responsible for the degradation of LSD1 in senescent cells.

## Discussion

**Figure 9:**
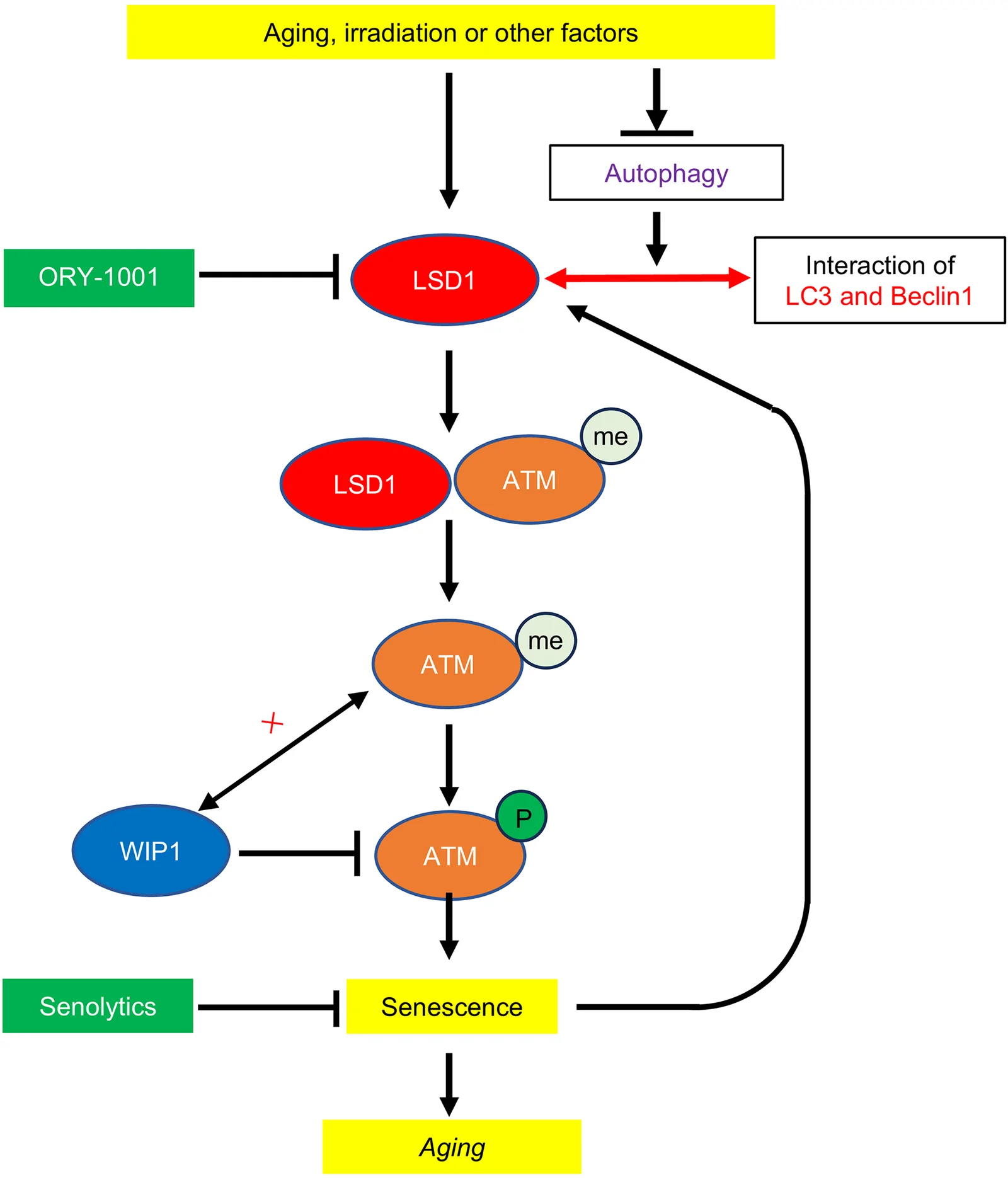
Working model. Cellular senescence is marked by a state of stable cell cycle arrest and plays a crucial role in aging and age-related diseases. DNA damage, a fundamental driver of cellular senescence, influences all hallmarks of aging. DNA damage response (DDR) is regulated either by histone modifications or by their interactions with and modifications of nonhistone substrates. In this study, we define the roles of lysine-specific histone demethylase 1 (LSD1) in senescence and aging and mechanistically address (a) a crosstalk of LSD1 and ATM activation, and (b) how the stability of LSD1 is regulated by autophagy during senescence and organ aging. Aging, irradiation or other factors led to increased expression of LSD1. In response to DNA damage, upregulation of LSD1 results in: (a) LSD1 interaction with and demethylation of ATM at its Lysine-3016 (K3016), (b) LSD1 mediated demethylation of ATM and inhibition of its interaction with WIP1, which blocks DNA damage repair and promotes cellular senescence, (c) aging-induced impairment of autophagy, leading to a reduced degradation of LSD1, (d) LC3 and Beclin1 interaction with LSD1, subjecting it to autophagy-mediated degradation. Thus, treatment with senolytic drugs (Dasatinib and Quercetin) to eliminate senescent cells reduces LSD1 expression, and inhibition of LSD1 by its specific inhibitor ORY-1001 ameliorates cellular senescence and organ aging.

Cellular senescence and DNA damage are central drivers of aging; however, how epigenetic mechanisms intersect with these processes remains incompletely understood. Here, we identify a role for LSD1 in regulating senescence and organ aging and define mechanisms governing its stability. We show that LSD1 is upregulated in senescent cells and aged organs, where it interacts with and demethylates ATM at K3016. This modification is associated with sustained ATM phosphorylation, impaired DNA damage checkpoint recovery, and persistent DDR signaling, features linked to senescence and tissue dysfunction. Inhibition or knockdown of LSD1 promotes ATM dephosphorylation via WIP1 and facilitates DNA repair (Figure 9). In vivo, pharmacologic inhibition of LSD1 with ORY-1001 attenuates age- and irradiation-induced tissue injury and reduces hair graying, supporting a functional role for LSD1 in aging phenotypes. Additionally, clearance of senescent cells reduces LSD1 protein levels, suggesting a feedback relationship between LSD1 and senescence. We further demonstrate that LSD1 is regulated posttranslationally through autophagy. LSD1 interacts with LC3 and Beclin1 and undergoes autophagosome-lysosome degradation. Impaired autophagy in senescent cells and aged tissues leads to LSD1 accumulation, whereas autophagy activation reduces LSD1 protein levels, demethylase activity, and downstream DDR signaling. Notably, LSD1 accumulates in the nucleus under senescent conditions, where reduced nuclear autophagy is associated with enhanced LSD1-ATM interaction. These findings link declining autophagic capacity during aging to sustain LSD1 activity and prolonged DDR signaling.

Cellular senescence is triggered by diverse endogenous and exogenous stresses, including telomere dysfunction, oncogene activation, persistent DNA damage, epigenetic dysregulation, and mitochondrial impairment (6). Among these, DNA damage represents a central driver that influences multiple hallmarks of aging and promotes senescence across tissues (41). Posttranslational modifications are critical regulators of the DNA damage response (DDR) and DNA repair pathways, with coordinated inputs from phosphorylation, acetylation, and ubiquitylation shaping key outputs such as cell cycle arrest, DNA repair, apoptosis, and senescence (42, 43). In this context, the role of epigenetic enzymes that mediate methylation of nonhistone proteins in DDR regulation is an emerging area of investigation.

LSD1, the first identified lysine demethylase, regulates chromatin plasticity through the demethylation of histones and nonhistone substrates (22). LSD1 has also been implicated in the DNA damage response (DDR) through its recruitment to sites of DNA damage, accompanying local reduction of H3K4 demethylation (44). Although LSD1 demethylates H3K4me2 and H3K9me2 without affecting the corresponding monomethylated states (30), our data do not exclude the role of these monomethylated histones in mediating senescence in this context. Notably, we show that LSD1-mediated ATM demethylation operates predominantly in the context of DNA damage–induced senescence and aging, particularly at later stages of the response. This function is distinct from previously described chromatin- or metabolism-centered roles of LSD1, including HIF-1α signaling or Sirt4 induction, which are primarily linked to metabolic adaptation or stress responses (24, 25). Rather, our study defines a DNA damage–specific role of LSD1 in DNA damage–induced senescence and aging, which complements previously described metabolic and signaling pathways and links epigenetic regulation to sustained ATM activation and downstream effectors, including γH2AX, p16, p21, and SASP factors. These results underscore the versatility of LSD1 in coordinating diverse regulatory networks that govern cellular senescence and support a model in which context- and tissue-dependent LSD1 activities converge to drive aging-related phenotypes.

The DNA damage checkpoint transiently arrests cell cycle progression to allow time for DNA repair, relying on damage sensor proteins, such as ATM and ATR, to detect DNA damage and to initiate downstream signaling cascades (45). Upon completion of DNA repair, checkpoint signaling is terminated and cell cycle progression is resumed through a process termed checkpoint recovery (46). Persistent ATM activation is known to impair DNA repair and contributes to organ aging by promoting cellular senescence and tissue dysfunction (47). However, the mechanisms governing checkpoint recovery remain incompletely understood. We show that LSD1-mediated demethylation is dispensable for initial ATM activation but contributes to the persistence of ATM signaling. Notably, K3016, the site of LSD1-mediated demethylation, is also a known acetylation site linked to ATM activation (48), suggesting potential crosstalk between acetylation and methylation. Consistent with prior studies, K3016 acetylation predominates during the early phase of the DNA damage response (minutes to approximately 8 hours after IR) (48), whereas our results show that demethylation at this site becomes more prominent at later stages (approximately 12 hours after IR), coinciding with sustained ATM phosphorylation and delayed dephosphorylation and resulting in a defect of DNA damage repair. This study supports a temporally regulated model in which acetylation drives early ATM activation, while LSD1-mediated demethylation sustains ATM signaling at later stages, at least in part by modulating the ATM-WIP1 interaction. By prolonging ATM activity, LSD1 may limit checkpoint recovery, an essential step for restoring cell cycle progression following DNA repair (15), thereby contributing to persistent DDR signaling and cellular senescence.

Autophagy and DNA repair are essential for cellular homeostasis (49), and enhanced autophagy is associated with improved health span and longevity (50). This study identifies LSD1 as a substrate of autophagy, with its turnover dependent on core autophagy components, including LC3 and Beclin1, under both basal and senescent conditions. While LC3 is predominantly cytoplasmic under starvation conditions, it remains partially retained in the nucleus in senescent cells (37). Beclin1 functions in autophagy initiation (51), while LC3 promotes autophagosome formation, reflecting their distinct roles in autophagic flux and cellular proliferation and senescence (52). Notably, LC3 interacts more strongly with LSD1 than Beclin1, suggesting a primary role for LC3 in mediating LSD1 turnover. Although LSD1 is present in cytoplasmic puncta following IR treatment, it exhibits pronounced nuclear enrichment in senescent cells. Under senescent and aging conditions, reduced autophagy, accompanied by decreased LC3 and Beclin1, limits the degradation of LSD1 in both nuclear and cytoplasmic compartments, leading to its accumulation and enhanced LSD1-ATM interaction. Consistent with this model, pharmacologic activation of autophagy following DNA damage reduces LSD1 protein levels and demethylase activity following DNA damage, supporting posttranslational regulation of LSD1 by autophagy. Together, these findings indicate that impaired autophagy sustains LSD1 abundance and activity in senescent cells, particularly in the nucleus, thereby promoting persistent ATM signaling and reinforcing the temporal control of LSD1 during the DNA damage response.

Although we systematically define the role and mechanisms of LSD1 in senescence and aged organs, several limitations warrant consideration. First, the absence of a methylation-mimetic mutation precludes direct interrogation of the interplay between K3016 methylation and acetylation. Second, the lack of LSD1 catalytic mutants selectively targeting histone versus nonhistone substrates limits our ability to exclude additional contributing pathways. Third, although LSD1 inhibition attenuates hair graying, whether similar mechanisms extend to other aging phenotypes remains to be determined. Finally, as cellular senescence represents only one component of organismal aging, future genetic models and longitudinal studies are needed to establish the causal role of the LSD1-ATM axis in aging.

In conclusion, this study identifies LSD1 as a regulator of DNA damage-induced senescence and organ aging through modulation of ATM demethylation and signaling duration. LSD1 is controlled by autophagy-dependent degradation, linking impaired proteostasis to sustained DDR. This study suggests that targeting LSD1 may be a novel strategy to mitigate senescence-associated tissue dysfunction and aging-related phenotypes, such as hair graying, in elder populations.

## Methods

### Sex as a biological variable.

Our study utilized both male and female mice in comparable ratios and reported similar findings for both.

### Cell culture and reagents.

Mouse IMCD3 (mIMCD3) cells, HEK293T, and NIH/3T3 cells (American Type Culture Collection, ATCC) cells were maintained in DMEM (Invitrogen) supplemented with 10% FBS and 1% penicillin and streptomycin at 37°C. To induce cellular senescence, oxidative stress injury agent D-galactose (D-gal) (#G0750, sigma) and the DNA damaging agent etoposide (ETO) (#E1383, sigma) were placed. For ionizing radiation experiments, mIMCD3 cells and 3T3 cells were exposed with irradiation by the X-ray irradiator (RS-2000 X-ray irradiator) (53). The following inducers were obtained for the study: ORY-1001 (LSD1 inhibitor) was purchased from Selleckchem, Lys05 (lysosomal autophagy inhibitor) was acquired from MCE, rapamycin (lysosomal autophagy agonist) was obtained from Millipore Sigma, and Nicotinamide (inhibitors of Sirtuin family proteins) was purchased from Millipore (Cat. 481907). All stock solutions were stored at –20°C.

The RNA oligonucleotides that specifically targeted mouse LSD1 (sc-60971), mouse Beclin1 (sc-29798) and mouse LC3B (sc-43391) were purchased from Santa Cruz Biotechnology, Inc. The RNA oligonucleotides were transfected with DharmaFECT siRNA transfection reagent (Dharmacon). 48 hours after transfection, cells were harvested and analyzed by Western blotting.

### Plasmids.

The plasmids encoding GFP–LC3 wild-type, GFP-LC3B, GFP-ATG5, GFP-ATG7, GFP-ULK1, and GFP-ATG13 were purchased from Addgene, and their constructs have been previously described (54). The plasmid encoding GFP-Beclin1 was purchased from Origin, and its construct has also been previously described (54). The GFP-tagged LSD1 plasmid was constructed by cloning full-length *Lsd1* into the pAc-GFP-C1 vector (Clontech). The truncated LSD1 was generated from GFP-LSD1 for direct transfection. The Flag-tagged wild-type ATM plasmid was purchased form Addgene (#31985). The Flag-tagged ATM-K1992R, Flag-tagged ATM-K3016R and Flag-tagged ATM-K3016Q mutant plasmid were purchased from Tsingke Biotech Co., Ltd. After 48 hours, the cells were harvested for further analysis. All constructs in this study were verified by DNA sequencing.

### The isolation of mouse primary tubular cells.

After excision of the renal capsules and medulla, kidney sections were finely minced and incubated in 10ml of a 1% collagenase type I buffer at 37°C with gentle rotation for 30 minutes. Subsequently, undigested kidney tissues were eliminated using a 70 μm filter. Following centrifugation, the cell pellet was washed and cultured in DEME/F12 medium supplemented with 10% FBS along with penicillin and streptomycin.

### RNA extraction and qRT-PCR.

Total RNA was extracted using the RNeasy Plus Mini Kit (QIAGEN). Total RNA (1 μg) was used for reverse transcription reactions in a 20 μl reaction to synthesize cDNA with an iScript cDNA Synthesis Kit (Bio-Rad). RNA expression profiles were analyzed by real-time PCR using iTaq SYBR Green Supermix with ROX (Bio-Rad) in an iCycler iQ Real-Time PCR Detection System. The complete reactions were subjected to the following program of thermal cycling: 40 cycles of 10 seconds at 95°C and 20 seconds at 60°C. A melting curve was run after the PCR cycles followed by a cooling step. Each sample was run in triplicate in each experiment, and each experiment was repeated three times. Expression levels of target genes were normalized to the expression level of actin. Relative changes in target mRNA expression were determined by using the 2^–ΔΔCT^ method.

### CRISPR-Cas9–based gene editing.

The ATM knockout (KO) 3T3 cell and mouse IMCD cell lines were generated via CRISPR-Cas9 technology. In brief, the guide RNAs (Sequence 1, F: CACCGTGATGCAGATACCAGATCCG; R: AAACCGGATCTGGTATCTGCATCAC; Sequence 2, F: CACCGGATCACGGAGTACATCCAG; R: AAACCTGGATGTACTCCGTGATCC; Sequence 3, F: CACCGCTTCTGCCTCAACAGCGACG; R: AAACCGTCGCTGTTGAGGCAGAAGC) were cloned into a p × 459/Puro vector, and then were transfected into 3T3 cells with Lipofectamine 3000 and selected as in the literature (55).

### Extraction of cytoplasmic and nuclear proteins.

Briefly, the cytosol extracts (C) and nuclear extracts (N) were obtained from whole-cell lysates. Cell pellets were collected and resuspended in a cytoplasmic extract buffer (10 mM HEPES pH 7.9, 10 mM KCl, 0.1 mM EDTA, 0.3% DS630). The resuspended cell pellet was vortexed for 5 min on ice followed by centrifugation at 3000 rpm for 5 minutes at 4°C to collect the supernatants as cytoplasmic extracts. The pellet was then resuspended and washed in a modified cytoplasmic extract buffer without DS630 before being centrifuged again at the same conditions mentioned above. Subsequently, the pellet was resuspended in a nuclear extract buffer composed of 20 mM HEPES pH7.9, 0.4 M NaCl, 1 mM EDTA,25% Glycerol, and protease inhibitors at a concentration of 1× and incubated on ice for 10 minutes. The samples were utilized for Western blot analysis as previous reported (56).

### Western blot analysis and immunoprecipitation.

Protein from renal and cultured cells was extracted using radio immunoprecipitation assay lysis buffer. Western blot analysis was performed as previously described (57). In brief, after nonspecific binding was blocked with 5% fat-free milk powder, nitrocellulose membranes were incubated overnight at 4°C with primary antibodies.

For immunoprecipitation, primary antibody or control IgG was coupled to protein A agarose bead (Pierce) in PBS containing 5 mg/ml bovine serum albumin (Sigma) for 6 hours at 4°C on a rotating platform. The cell lysates were then incubated with the beads coupled with antibody or control IgG overnight at 4°C. The next day, beads were washed with lysis buffer containing additional 300 mM NaCl and the immunoprecipitants were eluted off the beads using loading buffer with boiling for 5 minutes. The antibodies used for Western analysis included: anti-LSD1 (no. 17721, Abcam), anti-H3k4me1 (no. 8895, Abcam), anti-H3k4me2 (no. 7766, Abcam), anti-p16 (no. sc-1661, Santa Cruz), anti-p21 (no. sc-6246, Santa Cruz), anti-Beclin-1(no. sc-48381, Santa Cruz), anti-LC3 (no. 2775s, CST), anti-p62 (no. 23214, CST), anti-ATM (GTX111106, Gene Tex), anti-ATM-S1981p (GTX77613, Gene Tex), anti-cycling D (no. sc-8396, Santa Cruz), anti- γH2HX (no. 07627, Millipore Sigma), anti-H3 (no. sc-517576, Santa Cruz), anti-GFP (no. sc-9996, Santa Cruz), anti-pan-methylation (no. ab7315, Abcam), anti-actin (no. A5316, Sigma-Aldrich), anti-PCNA (no. 2586, CST), anti-cleaved caspase-3 (no. #9661, CST), anti-Flag (no. F3165, Sigma-Aldrich), anti-WIP1 (no. #94886, CST), anti-Lamin A/C (no. 10298-1-AP, Proteintech) and anti-pan-acetylated-lysine (no. 9681, CST) antibodies. Secondary antibody including donkey anti-rabbit IgG-horseradish peroxidase (sc-2313), and goat anti-mouse IgG-horseradish peroxidase (sc-2005) antibodies purchased from Santa Cruz Biotechnology. Donkey anti-goat IgG (H + L) horseradish peroxidase (no. A15999) was purchased from Thermo Fisher Scientific.

For immunoprecipitation, antibodies against LSD1, ATM, GFP, Flag (F3165, Sigma), LC3, or Beclin1, along with corresponding isotype control IgG, were coupled to Protein A agarose beads (Pierce) in phosphate-buffered saline (PBS) containing 5 mg/mL bovine serum albumin (Sigma-Aldrich) for 6 hours at 4°C with rotation. Cell lysates were then incubated with antibody-conjugated beads overnight at 4°C. The following day, beads were washed with lysis buffer supplemented with 300 mM NaCl, and immune complexes were eluted by boiling in SDS loading buffer for 5 minutes. Eluates were subsequently analyzed by Western blotting.

### Histology and IHC.

Paraffin-embedded kidney, heart, and liver sections (4-μm thickness) were prepared according to a standard procedure. Sirius staining was performed by using a standard protocol. For immunohistochemistry staining, a polyclonal rabbit Ki67 (ab15580) antibody (1:100 dilution), biotinylated secondary antibody (Santa Cruz Biotechnology Inc.; 1:100 dilution), and DAB substrate system were used. Kidney sections were counterstained by hematoxylin. Images were analyzed with a Nikon Eclipse 80i microscope.

### Immunofluorescence microscopy.

Cells on coverslips were fixed in pre-cold methanol for 10 minutes at –20°C, followed by permeabilization with 0.1% Triton X-100 for 15 minutes at room temperature. After blocking in 2% BSA, cells were incubated with appropriate primary and secondary antibodies. Images were acquired using a Zeiss confocal microscope LSM780 or a Nikon Eclipse 80i microscope. Three-dimensional structured illumination microscopy experiments were performed using a Zeiss ELYRA super-resolution microscopy system using 63 × 1.4 oil immersion lens. Statistical analyses were performed in GraphPad Prism version 8.0 (GraphPad Software).

### Generation of anti-ATM-K3016me antibodies.

The anti-ATM-K3016me antibody was generated by KMD Bioscience. Three methylated peptides and one unmethylated peptide were synthesized as follows: methylated peptide 1 (ERVLMLRLQE-Lys(Me)-LKG), methylated peptide 2 (QE-Lys(Me)-LKGVEEGTVLSV), methylated peptide 3 (LQE-Lys(Me)-LKG), and the unmethylated peptide (ERVLMLRLQEKLKGVEEGTVLSV). Peptides 1 and 2 were conjugated to keyhole limpet hemocyanin (KLH), while peptide 3 was conjugated to both KLH and BSA. The unmethylated peptide was conjugated to BSA as control. Mass spectrometry analysis before and after conjugation revealed changes in absorbance within the 200–1000 nm range, confirming successful conjugation (Supplemental Figure 7, A and B). Two rabbits that had not been previously immunized were immunized five times at indicated time points. The immunization sequence is as follows: peptide 1-KLH, peptide 2-KLH, peptide 3-KLH, peptide 3-BSA and peptide 4-BSA. ELISA analysis showed that the titer of peptide 3-BSA antibodies increased in rabbit 1# but remained unchanged in rabbit 2#, indicating a failed immune response in rabbit 2# (Supplemental Figure 7, C and D). Serum from rabbit 1# was collected and antibodies were purified using Protein A/G affinity chromatography. The purity of the antibodies was assessed by SDS-PAGE (Supplemental Figure 7E). Further purification was performed using affinity purification with methylated peptide antigens and negative selection with non-methylated peptides, followed by SDS-PAGE analysis to confirm antibody purity (Supplemental Figure 7F).

### GST pull-down assays.

GST pull-down assays were performed as previously described (57). In brief, the GST-LSD1 fusion protein-expressing constructs were generated in a pGEX-6p-1 vector (GE Healthcare) by cloning fragments of human LSD1 complementary DNA (cDNA) (GenScript) into the Eco RIand Xho I sites via polymerase chain reaction (PCR), utilizing the In-Fusion HD Cloning Kit (Clontech). GST-LSD1 constructs were transformed to BL21 E. coli and induced using 0.2 mM isopropyl-β-d-thiogalactopyranoside (IPTG). The GST-LSD1 fusion proteins were purified using glutathione-agarose beads (Pierce), and expression of the constructs was analyzed on SDS-PAGE gels stained with Coomassie blue. The purified GST-LSD1 fusion proteins were incubated with recombinant LC3B protein (ab103506, abcam) and recombinant Beclin1 protein (ab137161, abcam), each at a concentration of 1 μg. Following washing with PBST buffer, the protein complexes were separated by SDS-PAGE gels and subjected to immunoblot analyses using anti-LSD1 antibody.

### Comet assay.

Comet assay was performed using a commercial kit (ab238544, Abcam) according to the manufacturer’s instructions.

### SA-β-gal staining.

According to the manufacturer’s instructions, the cultured cells and frozen tissues (kidney, heart and liver) slices were fixed and stained using an SA-β-gal staining kit (9860, Cell Signaling Technology).

### Terminal deoxynucleotidyl transferase-mediated dUTP nick end-labeling (TUNEL) assay.

TUNEL assays were performed on mouse IMCD cells following DNA damage using the In Situ Cell Death Detection Kit, Fluorescein (Roche), according to the manufacturer’s instructions. Nuclei were counterstained with ProLong Gold Antifade Mountant containing DAPI (Thermo Fisher Scientific). Fluorescence images were captured using a Nikon Eclipse 80i microscope under standardized exposure conditions, with identical acquisition parameters applied across all experimental groups.

### Protein-protein docking test.

The amino acid sequences of LSD1(Uniprot ID: Q6ZQ88), Beclin1 (Uniprot ID: O88597), and LC3 (Uniprot ID: Q9CQV6) were downloaded from Uniprot (https://www.uniprot.org/). The three-dimensional structures and interactions of the complexes between LSD1 and Beclin1, as well as between LSD1 and LC3, were computationally predicted using AlphaFold-Multimer. To achieve more stable systems, molecular dynamics simulations of 50 ns were performed for each protein interaction pair using GROMACS. Interaction information was analyzed after the molecular motion trajectories reached equilibrium. The test was conducted by Shanghai Yuyibiotech Inc. (58, 59).

### Animals and treatments.

C57BL/6J mice were purchased from the Jackson Laboratory. Mice were maintained in a pathogen-free facility at 23–24°C with relative humidity no higher than 50 percent under a 12-hour light-dark cycle regimen with free access to a NCD (standard mouse diet Lab Diet 5053) and water. Both male and female mice were used for the study, and the age was indicated in the figure legends.

Total 16 C57BL/6J mice were exposed to 7 Gy TBI at a dose rate of 0.99Gy/min (Precision X-Ray) (225kVp, 20mA). To examine the role of LSD1 in irradiation-induced cellular senescence, LSD1 inhibitor (0.04 mg/kg) in DMSO was administered intraperitoneally after irradiation treat every two days.

Natural aging mice (22-month-old) treated with or without LSD1 inhibitor (0.04 mg/kg) every 2 days for 6 months. In parallel, natural aging mice (20–22-month-old) treated with or without rapamycin (8 mg/kg) every 2 days for 2 weeks, and young mice (2-month-old) treated with or without lys05 (20mg/kg) for 2 weeks. Additionally, natural aging mice (20–22-month-old) treated with or without 2 senolytic drugs, Dasatinib (D; an FDA-approved tyrosine kinase inhibitor) and Quercetin (Q; a flavonoid present in many fruits and vegetables). For D + Q treatments, D (5 mg/kg) and Q (50 mg/kg) were administered via oral gavage in 100 μl PBS every other day for 1 month. D and Q were purchased from Santa Cruz Biotechnology, Inc.

### Statistics.

All data are presented as mean ± SEM. All statistical analyses were performed using GraphPad Prism (GraphPad Software). All in vitro experiments were independently repeated at least 3 times. Comparisons between 2 groups were performed using a 2-tailed unpaired Student’s *t* test, whereas comparisons among multiple groups were analyzed by 1-way ANOVA. A *P* value < 0.05 was considered statistically significant.

### Study approval.

All animal experiments were performed in accordance with protocols approved by the Institutional Animal Care and Use Committee (IACUC) of Mayo Clinic (No. A00003756-18-R24, Rochester, Minnesota, USA). No human subjects were involved in this study.

### Data availability.

The ChIP-seq datasets supporting the findings of this study have been deposited in the Gene Expression Omnibus (GEO) under accession numbers GSE106145, GSE38442. Source data underlying all graphs are provided in the Supporting Data Values file.

## Author contributions

YZ performed most of experiment and prepared the manuscript. XZ and LXL performed some of the experiments and data analysis. ASC and CY performed data analysis. XL supervised the whole project and manuscript editing.

## Confict of interest

The authors have declared that no conflict of interest exists.

## Funding support

This work was funded, in whole or in part, by the NIH and is therefore subject to the NIH Public Access Policy. In accordance with this policy, the NIH retains the right to make the final peer-reviewed manuscript publicly available through PubMed Central.

- National Institutes of Health (NIH) grants R01 DK129241 and R01 DK126662 (to XL).
- U.S. Department of Defense Focused Program Award PR221810 (to XL).

## Supplementary Material

Supplemental data

Unedited blot and gel images

Supporting data values

## Version 1 09/15/2026

Electronic publication
