# Supplementary material for: LSD1-mediated demethylation of the DNA damage response factor ATM promotes senescence and organ aging
Source: J Clin Invest. 2026 Sep 15;136(18):e199854. doi: 10.1172/JCI199854 (PMC13574142; doi:10.1172/JCI199854)
Supplement: Supplementary file 1 [file jci-136-199854-s217.pdf]

Supplementary Figures and Legends

Supplementary Figure 1

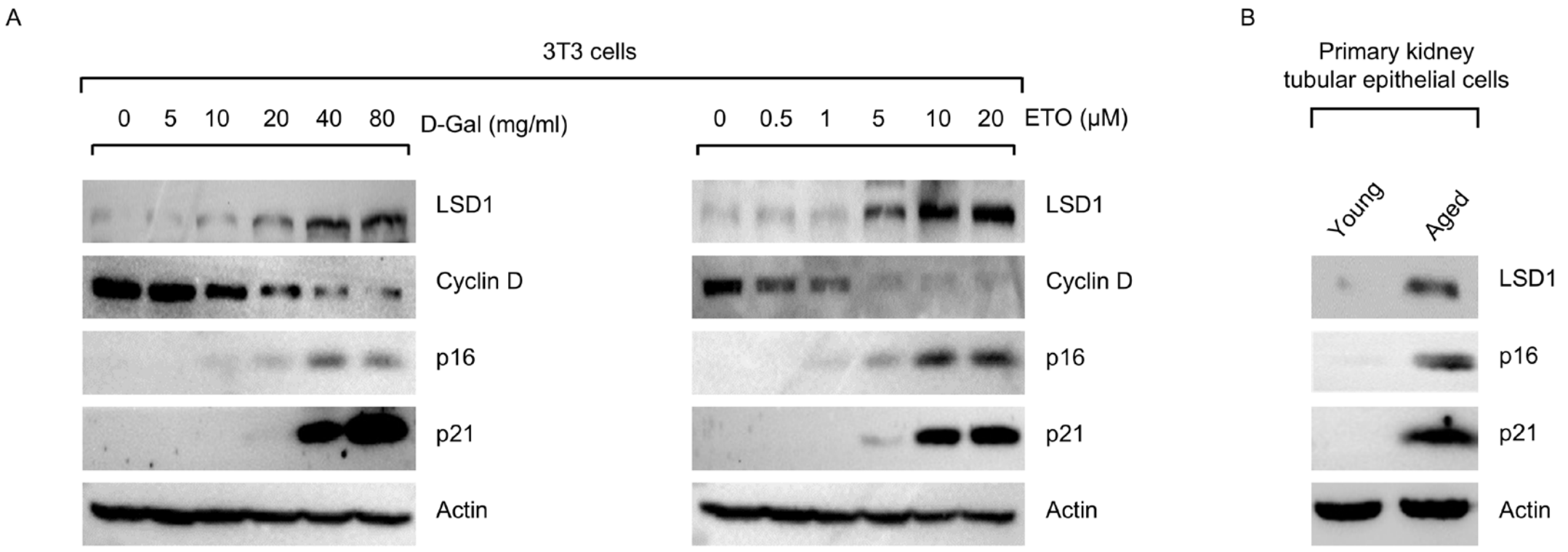

Supplementary Figure 1. The expression of LSD1 is increased in senescent 3T3 and primary kidney tubular epithelial cells. (**A**) Western blot analysis of the levels of LSD1, Cyclin D, p16 and p21 in mouse 3T3 cells treated with different dose of D-Gal and etoposide (ETO). n=3. (**B**) Primary tubular epithelial cells (TECs) were isolated from 2-month-old mice (young) and 22-month-old mice (aged). Western blot analysis of the levels of p16 and p21 in primary tubular epithelial cells (TECs) isolated from kidneys of 2-month-old mice (young) and 22-month-old mice (aged). n=3.

Supplementary Figure 2

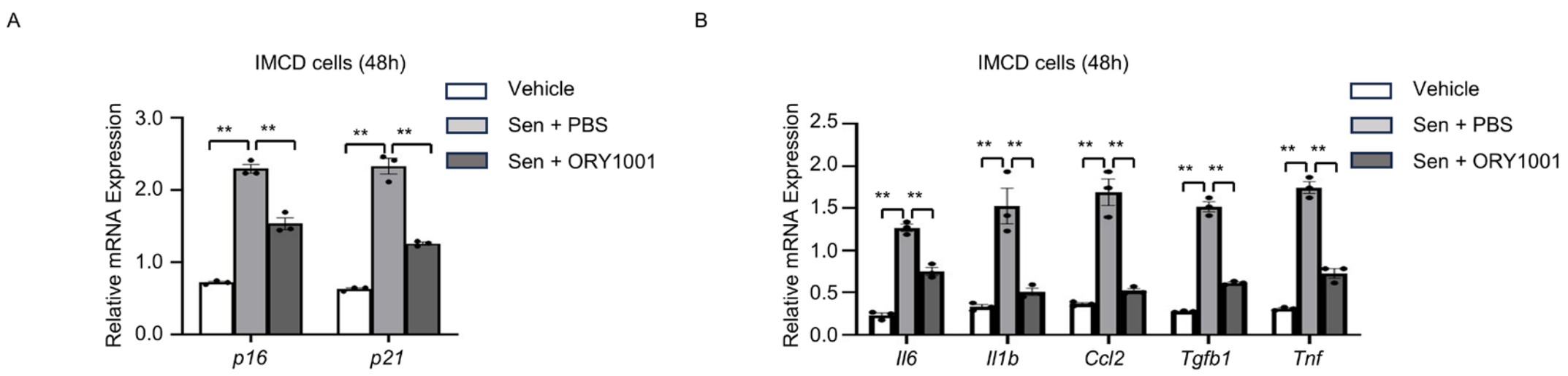

Supplementary Figure 2. Inhibition of LSD1 reduces cellular senescence and the expression of senescence-associated secretory phenotype (SASP) factors in mouse IMCD cells. (**A, B**) qRT-PCR analysis of *p16* and *p21* mRNAs (**A**) and Key SASP cytokines, including *Il6, Il1b, Ccl2, Tgfb1* and *Tnfa.* (**B**) in mouse IMCD cells treated with or without ORY1001 following ETO treatment. (Statistical analysis of the three independent expression; $^{**}p < 0.01$; one-way ANOVA)

Supplementary Figure 3

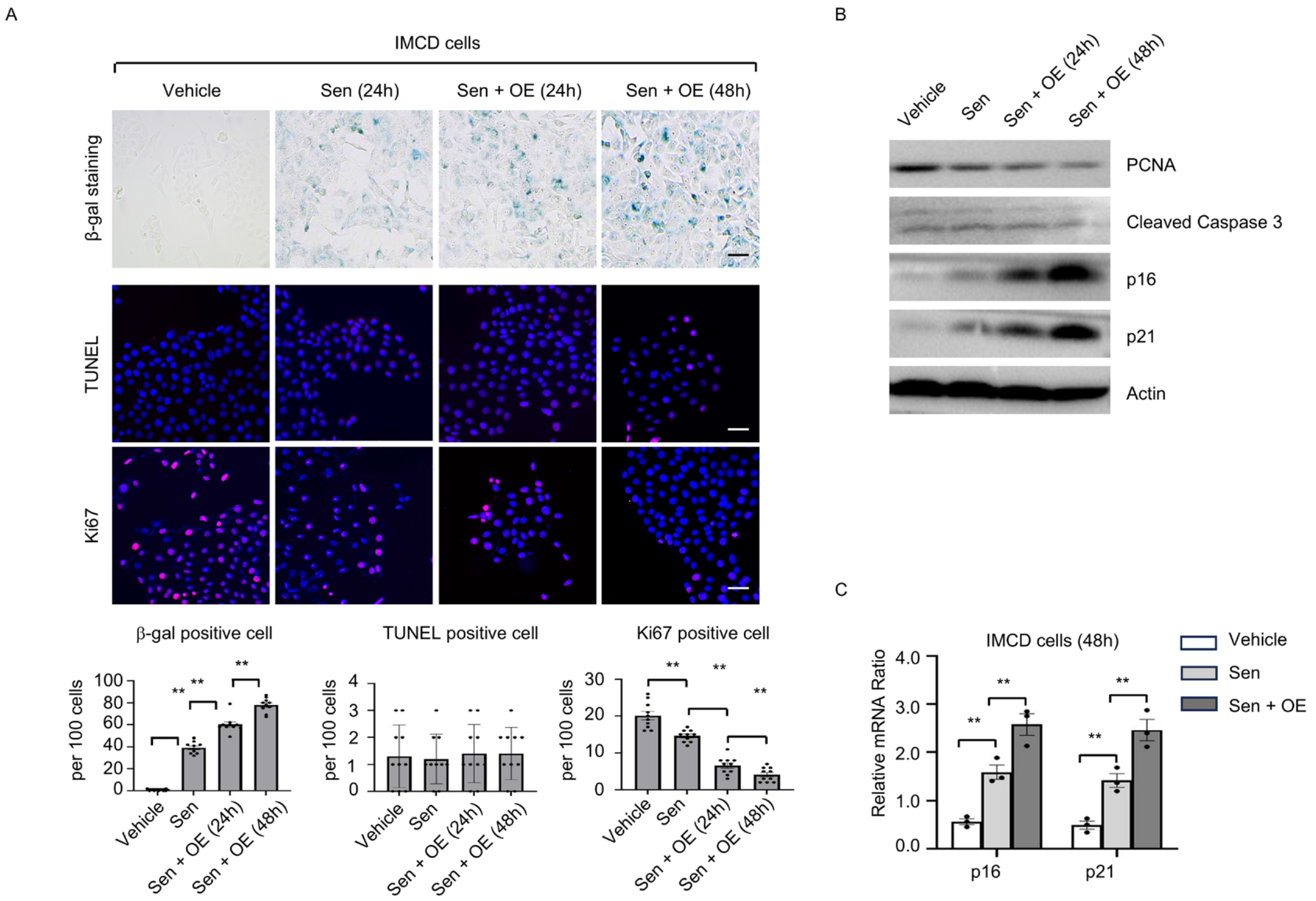

Supplementary Figure 3. LSD1 overexpression enhances cellular senescence in a time dependent manner. (**A**) Representative images and quantification of SA-β-gal staining (*top*), TUNEL assay (*middle*), and Ki67 staining (*bottom*) in mouse IMCD cells overexpressing LSD1 at 24 hours and 48 hours after ETO (5 μM) treatment. bar=50μm. (The percentage of positive cells was quantified using the same software in 10 randomly selected fields; $^{**}p < 0.01$; one-way ANOVA) (**B**) Western blot analysis of LSD1, PCNA, cleaved caspase-3, p21, and p16 in GFP-LSD1-overexpressing senescent mouse IMCD cells at 24 and 48 hours post-ETO treatment. Represent data from 3 independent experiments are shown. (**C**) qRT-PCR analysis of *p16* and *p21* mRNAs in mouse IMCD cells with or without LSD1 overexpression at 48 hours after ETO (5 μM) treatment. (All experiments were repeated at least 3 times; $^{*}p < 0.05$; one-way ANOVA)

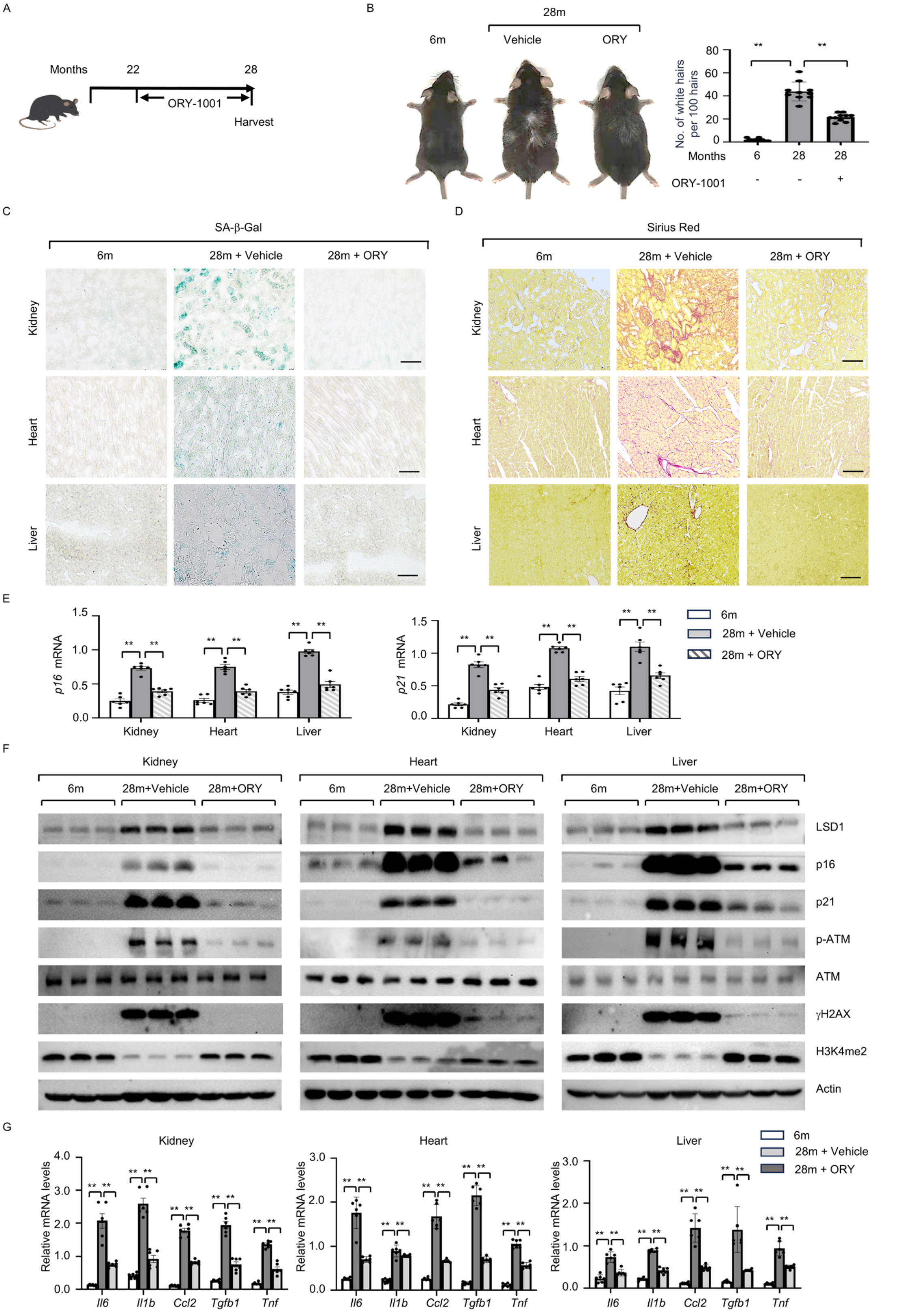

Supplementary Figure 4. Treatment with ORY-1001 decreases natural aging-induced DNA damage in mouse organs and protects hair graying. (A) Schematic of the ageing

and ORY-1001 treatment regimen in C57BL/6 mice (treatment from 22 to 28 months of age). (**B**) Representative images of mice from the young (6-month-old), aged (28-month-old), and aged plus ORY-1001 treatment groups. Quantification of gray hair is shown as the number of gray hairs per 100 strands (mean ± SEM). (n=10; ***p* < 0.01; one-way ANOVA) (**C-D**) SA-β-gal staining (**C**) and Sirius Red staining (**D**), analysis in mouse organs, including kidneys (bar=100μm); heart (bar=100μm); and livers (bar=50μm) from aged and aged plus ORY-1001 treated groups. n=6. (**E**) qRT-PCR analysis of *p16*, *p21* mRNAs in kidney, heart and liver tissues. n=6. (**F**) Western blot analysis of the levels of LSD1, p16, p21, phospho-ATM (p-ATM), total ATM, and in mouse organs, including kidney (*left*), heart (*middle*) and livers (*right*) from aged and aged plus ORY-1001 treated groups. n=6. (**G**) qRT-PCR analysis of *Il6, Il1b, Ccl2, Tgfb1* and *Tnfa* mRNAs in kidney, heart and liver tissues. (n=6/group; ***p* < 0.01; one-way ANOVA)

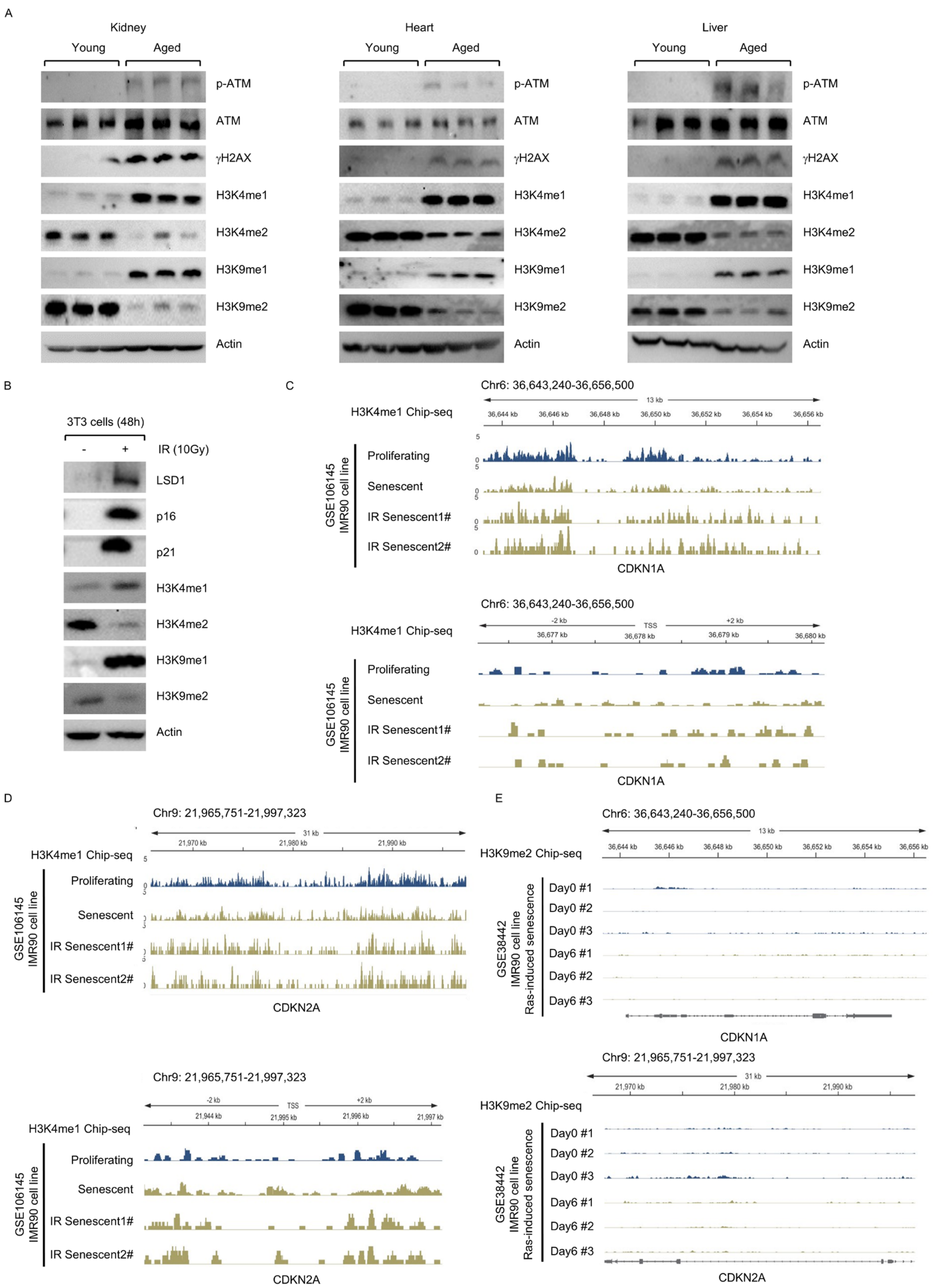

Supplementary Figure 5. DNA damage signaling and histone methylation are altered in aged organs and senescent cells. (**A**) Western blot analysis of the levels of phospho-ATM (p-ATM) and total ATM, H3K4me1/2, H3K9me1/2 in mouse organs, including kidneys (*left*), heart (*middle*) and livers (*right*) from young (2-month-old) and aged (22-24-month-old) mice.

(**B**) Western blot analysis of LSD1, p16, p21, H3K4me1, H3K4me2, H3K9me1, H3K9me2 in mouse 3T3 cells with or without IR treatment for 48 hours. (**C-D**) Integrative Genomic Viewer (IGV) tracks showing H3K4me1 ChIP-seq signals at the *CDKN1A* (**C**) and *CDKN2A* (**D**) locus in human fibroblast cells (IMR90) under different statuses (GSE106415). (**E**) Integrative Genomic Viewer (IGV) showing the H3K9me2 ChIP-seq signals at the *CDKN1A* locus in human fibroblast cells (IMR90) in different statuses (GSE38442).

Supplementary Figure 6

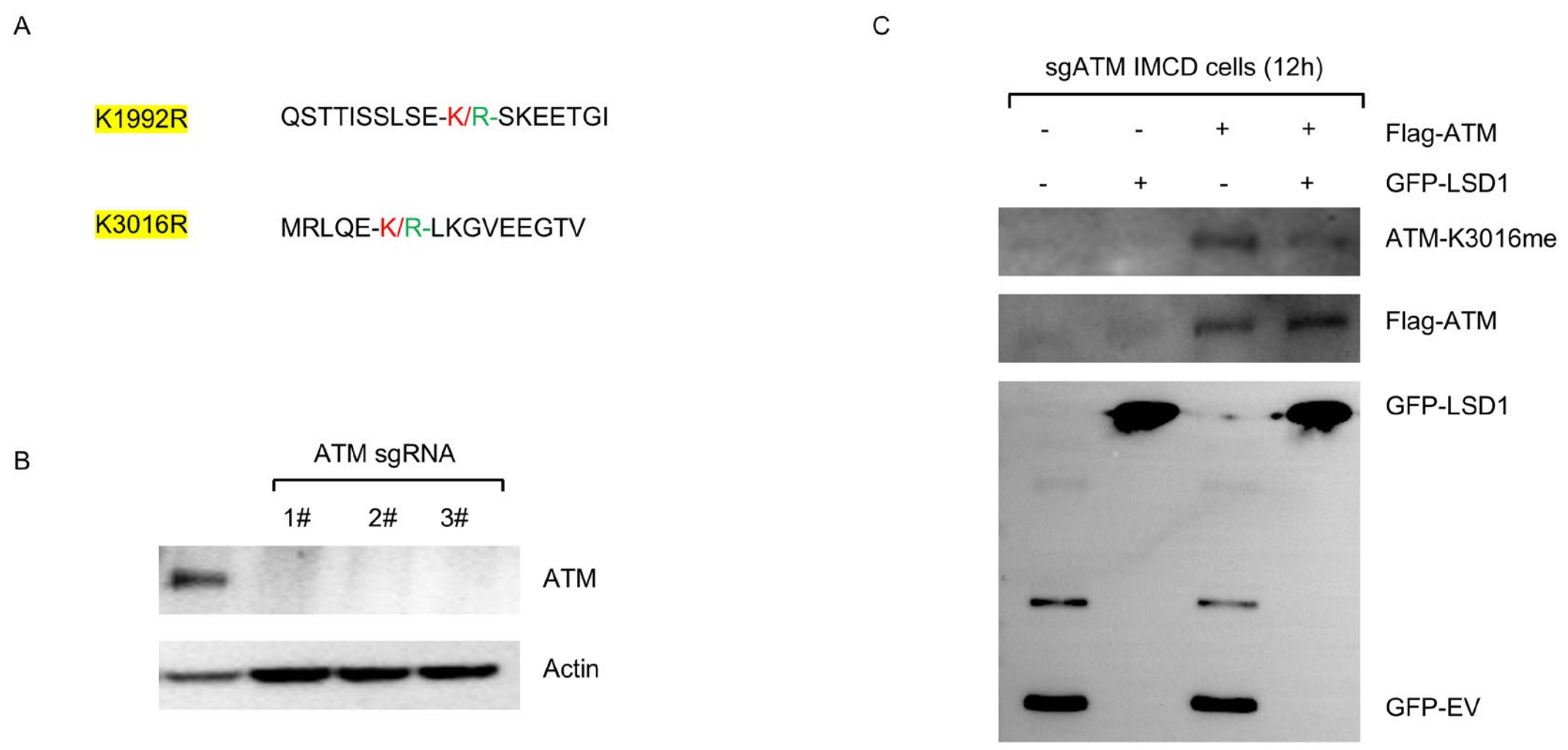

Supplementary Figure 6. Generation of ATM knockout cell lines. (**A**) Alignment of human ATM amino acid sequences surrounding lysine 1981 and lysine 3016. Substitution of Lysine (R) with Arginine (K) was indicated. (**B**) Western blot analysis of ATM expression in mouse 3T3cells transfected with three sgRNAs targeting ATM (sgATM). (**C**) Western blot analysis of the levels of ATM-K3016 methylation, Flag-tagged ATM and GFP-tagged LSD1 in ATM knockout mouse IMCD cells transfected with the indicated plasmids at 12 hours post-IR.

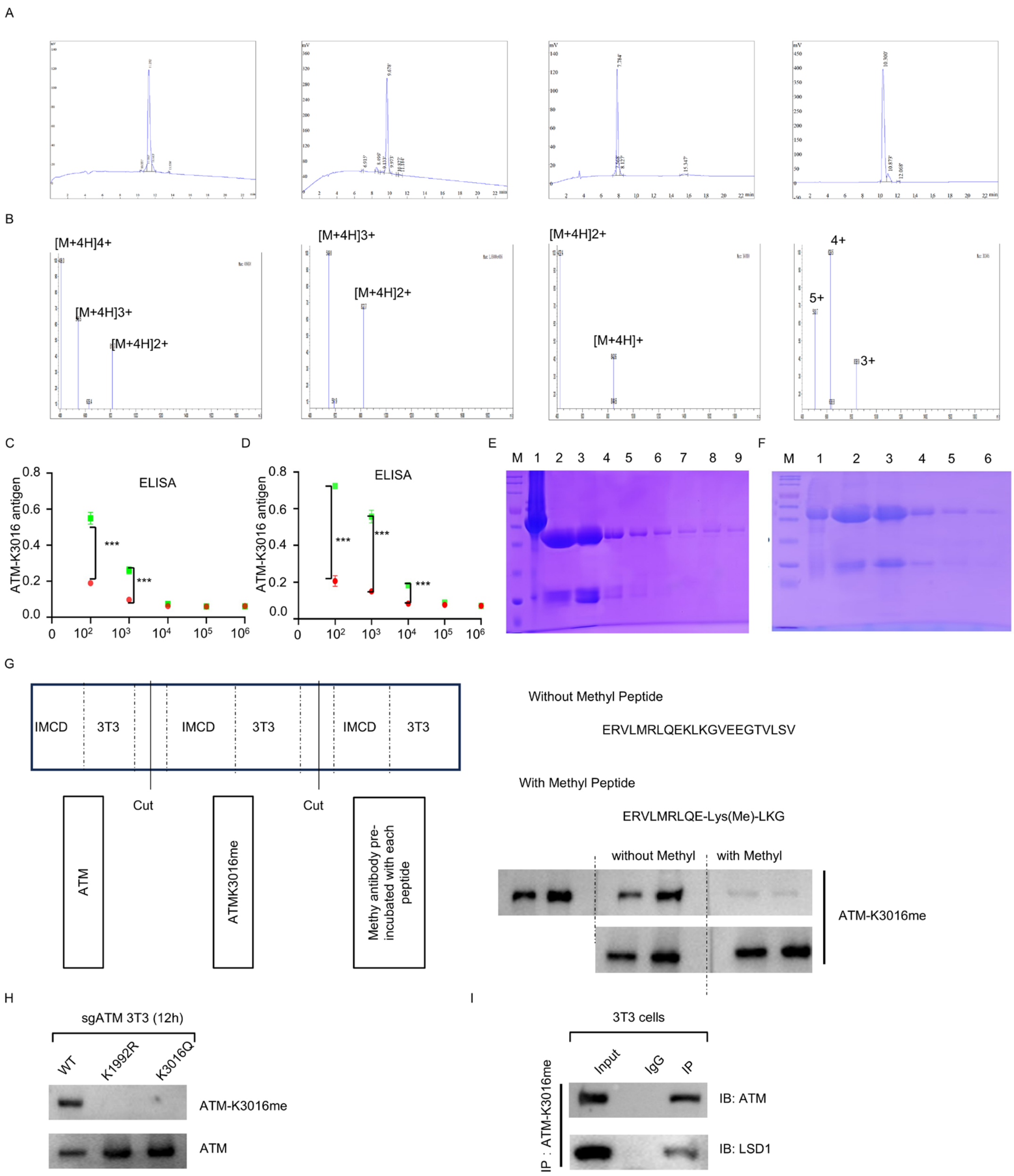

Supplementary Figure 7. Generation and validation of the ATM-K3016me antibody. (**A**) High-performance liquid chromatography (HPLC) analysis confirmed the successful synthesis of three methylated peptides and one unmethylated peptide. (**B**) Mass Spectrometry (MS) analysis further validated the successful synthesis of three methylated peptides and one unmethylated peptide surrounding lysine 3016. (**C**) ELISA analysis detects the immunogenicity titer of serum ATM-K3016me following four polypeptide immunizations in rabbit, comparing peptide-immunized group (*green*) and control (*red*) groups. HRP-conjugated goat anti-rabbit IgG (100 μl) (diluted 1:15000) was added to each well, followed

by the addition of 100 μl of tetramethylbenzidine (TMB) substrate solution. Absorbance readings were taken at a wavelength of 450 nm. (**D**) ELISA analysis detects the immunogenicity titer of serum following five peptide immunizations in rabbit. (**E**) The antibodies were purified by Protein A/G beads and the purity of the antibodies was assessed by SDS-PAGE. (**F**) SDS-PAGE analysis of the purity of methylated peptide antigens following affinity purification. (**G**) Peptide competition assay for ATM-K3016me antibody. Cell lysates (50 μg) from IMCD cells and 3T3 cells were run on 6% SDS-PAGE gels, transferred to PVDF membranes, and then immunoblotted with ATM-K3016 antibody pre-incubated with un-methylated and methylated peptides for 60 minutes at room temperature. Membranes were processed and exposed under identical conditions. (**H**) Western blot analysis of the levels of ATM-K3016 methylation in ATM knockout mouse 3T3 cells transfected with the indicated plasmids. (**I**) Immunoprecipitation assay assessing the interaction between ATM-K3016 methylated and LSD1 using ATM-K3016me antibody. ***$p < 0.001$.

Supplementary Figure 8

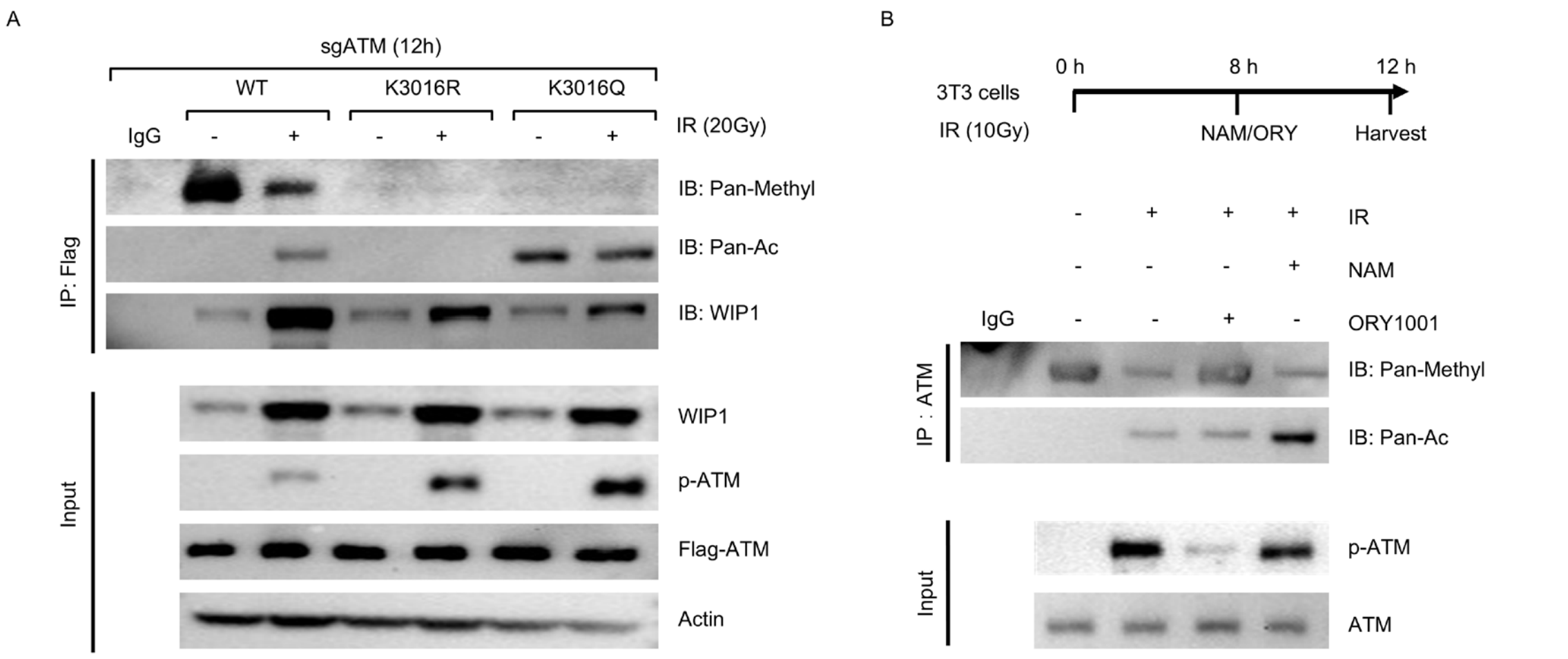

Supplementary Figure 8. The acetylation and methylation of ATM at K3016 is detected upon DNA damage. (**A**) Immunoprecipitation assay detecting the methylation and the acetylation of ATM, as well as the interaction between Flag-tagged ATM and WIP1, in ATM knockout 3T3 cells transfected with the indicated plasmids with or without IR (20 Gy) treatment. (**B**) Immunoprecipitation assay detecting the methylation and acetylation of ATM following NAM or ORY treatment at 8 hours post IR (10 Gy) treatment for 4 hours in 3T3 cells.

Supplementary Figure 9

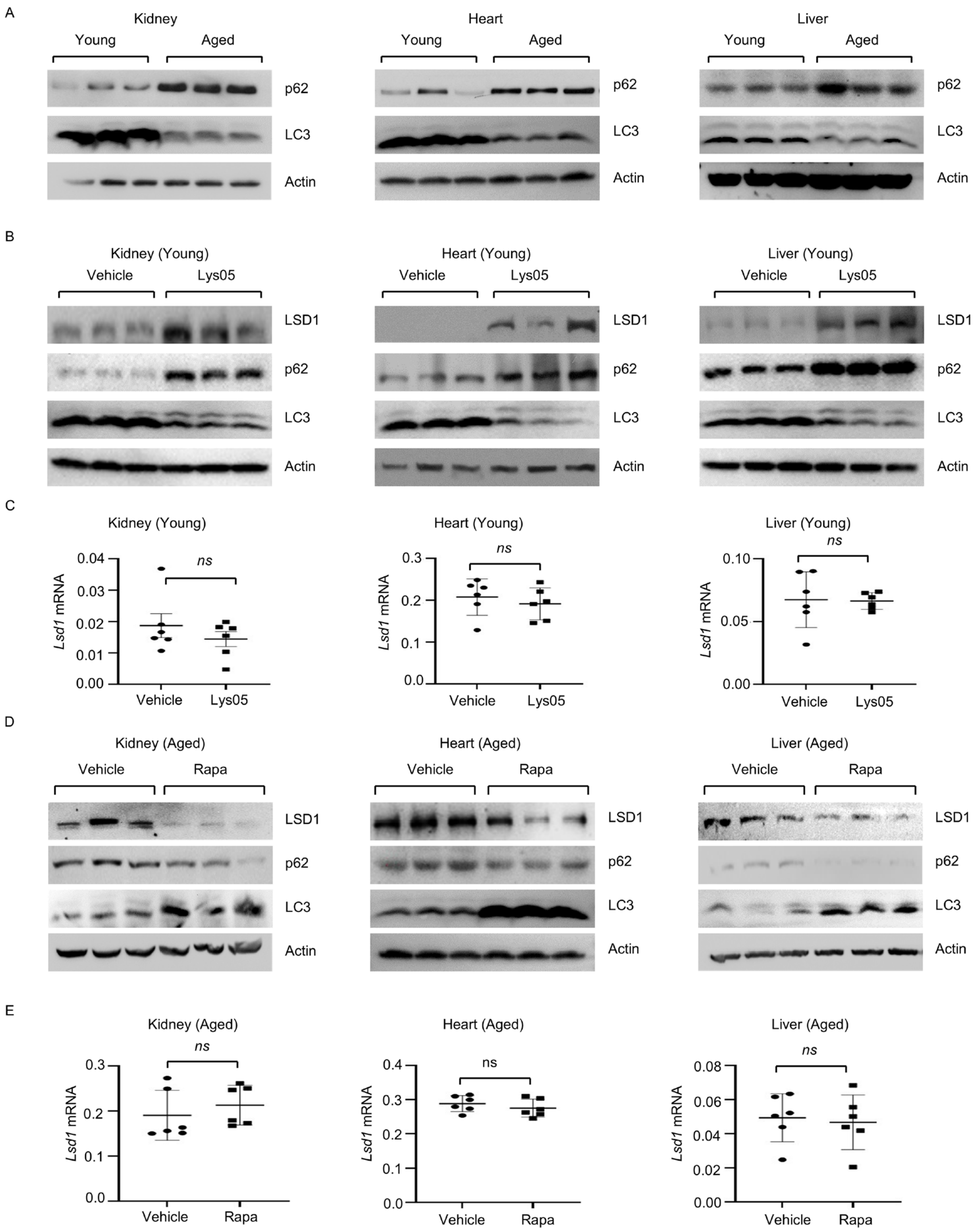

Supplementary Figure 9. Autopage modulates the stability of LSD1 protein in young and aged mouse organs. (**A**) Western blot analysis of the expression of p62 and LC3 in mouse organs, including kidneys (left), hearts (middle), and livers (right), collected from 20-22-months-old mice (*aged*) compared to that in organs collected from 2-month-old mice (*young*). (**B**) Western blot analysis of the expression of LSD1, p62 and LC3 in organs, including kidneys (*left panel*), heart (*middle panel*) and livers (*right panel*) collected from 2-

month-old mice treat with Lys05 or vehicle. (**C**) qRT-PCR analysis of *Lsd1* mRNA in mouse organs, including kidneys, hearts and livers, treated with Lys05 and vehicle. (n=6/group; **$p < 0.01$; unpaired, 2-tailed Student's *t* test) (**D**) Western blot analysis of the expression of LSD1, p62 and LC3 in organs, including kidneys (*left panel*), heart (*middle panel*) and livers (*right panel*), collected from aged mice treated with rapamycin and vehicle. (E) qRT-PCR analysis of *Lsd1* mRNA in aged organs, including kidneys, hearts and livers, treated with rapamycin and vehicle. (n=6/group; ns, not significant; unpaired, 2-tailed Student's *t* test)

Supplementary Figure 10

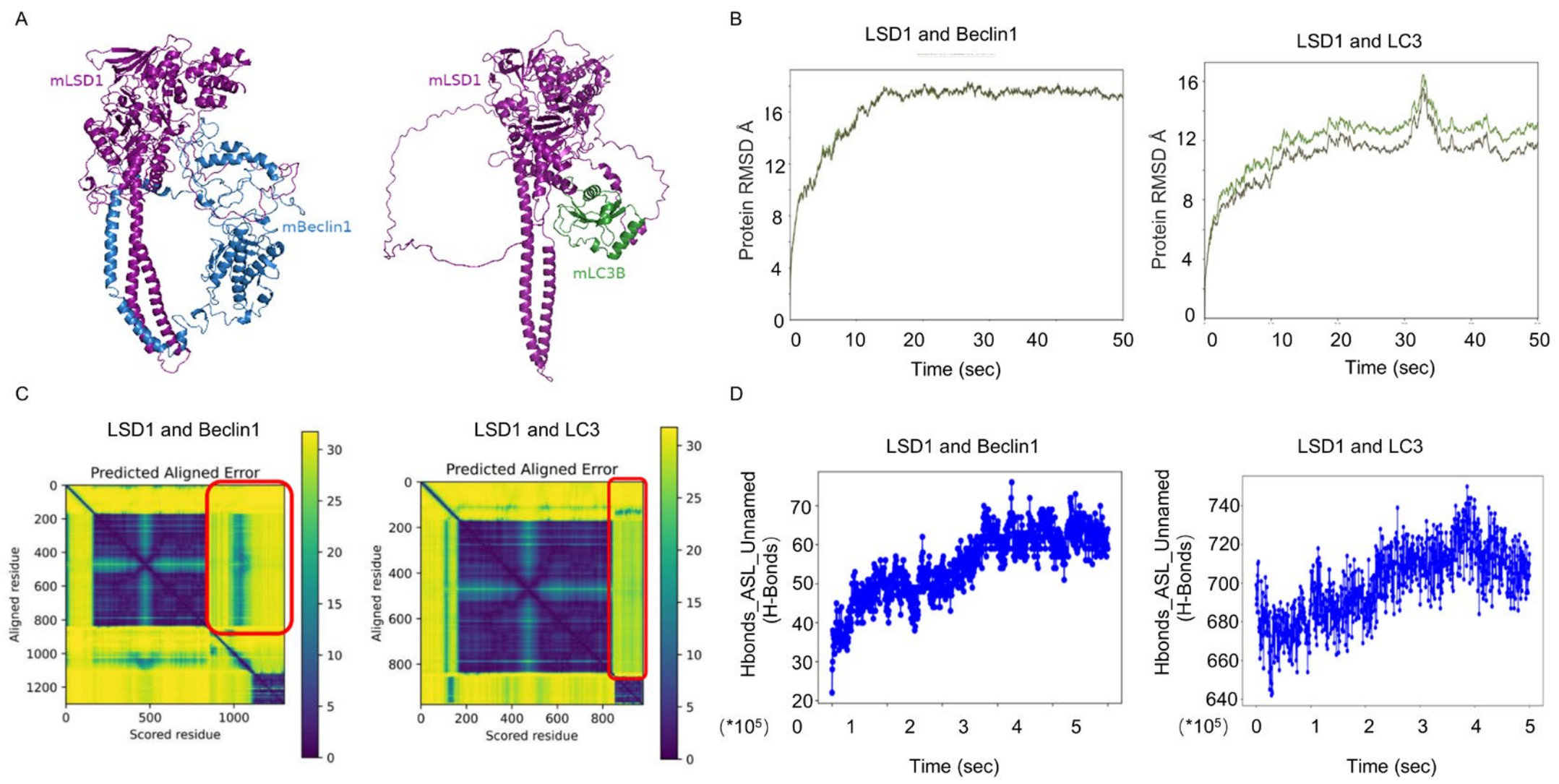

Supplementary Figure 10. LSD1 interacts with LC3 and Beclin1 by molecular dynamics simulation experiments. (**A**) Uniport acquired the complete structural models of mLSD1 (*purple*), mBeclin1 (*blue*) and mLC3B (*green*). (**B**) The Root Mean Square Deviation (RMSD) value of the protein backbone of LSD1 and LC3 as well as LSD1 and Beclin1, which was calculated based on backbone atoms. (**C**) The calculation of the predicted aligned error (pAE) value between LSD1 and Beclin1 as well as LSD1 and LC3 by using AlphaFold multimer. (**D**) The detection of the dynamic changes of hydrogen bonds between LSD1 and Beclin1 as well as LSD1 and LC3B by using the molecular dynamics simulation-driven method.
