# Supplementary material for: LSD1-mediated demethylation of the DNA damage response factor ATM promotes senescence and organ aging
Source: J Clin Invest. 2026 Sep 15;136(18):e199854. doi: 10.1172/JCI199854 (PMC13574142; doi:10.1172/JCI199854)

Figure 1A-1C

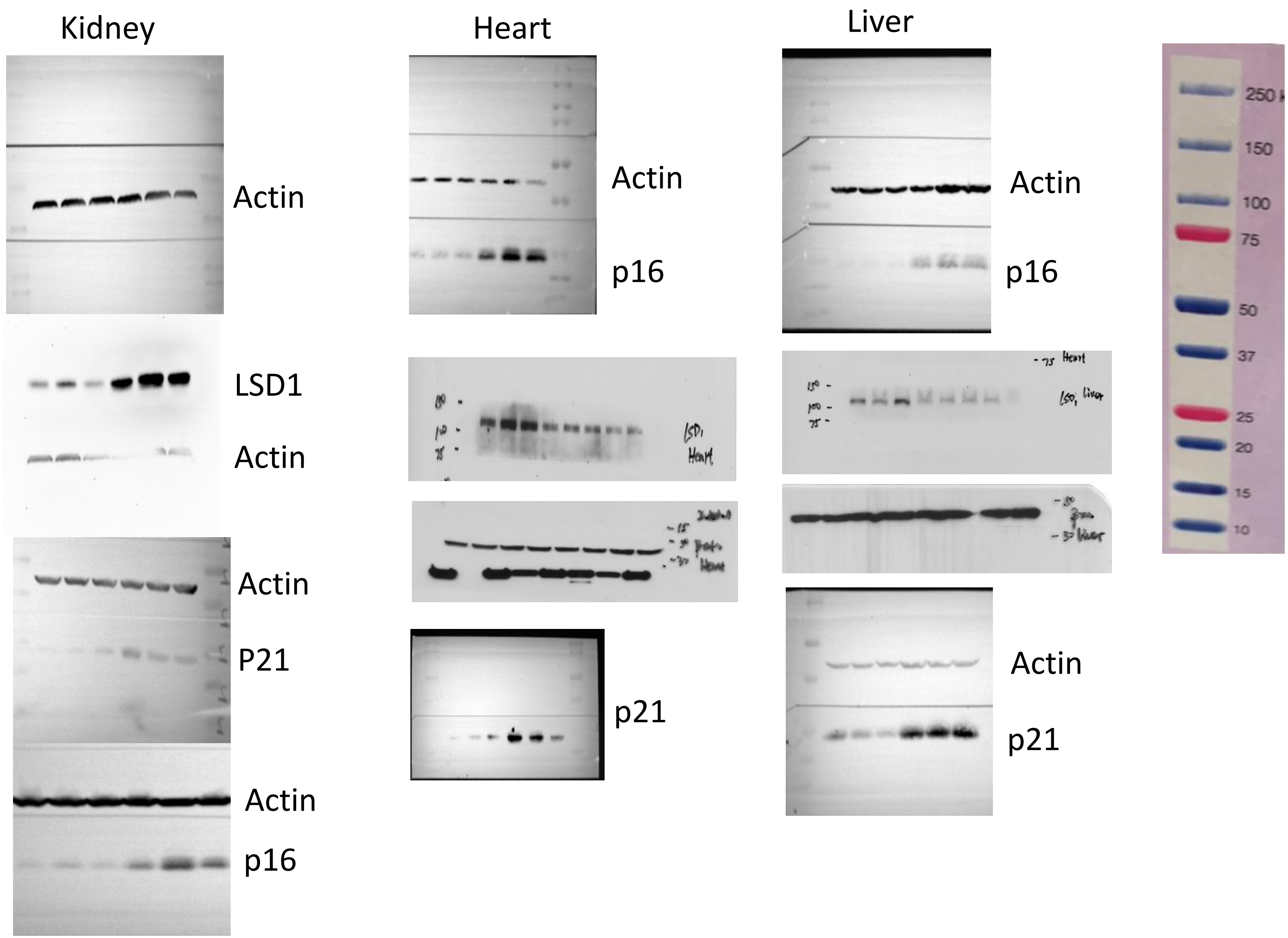
Kidney
Heart
Liver
Actin
LSD1
Actin
Actin
P21
Actin
p16
Actin
p16
p21
Actin
p16
Actin
p21
250
150
100
75
50
37
25
20
15
10

Figure 1D -1F

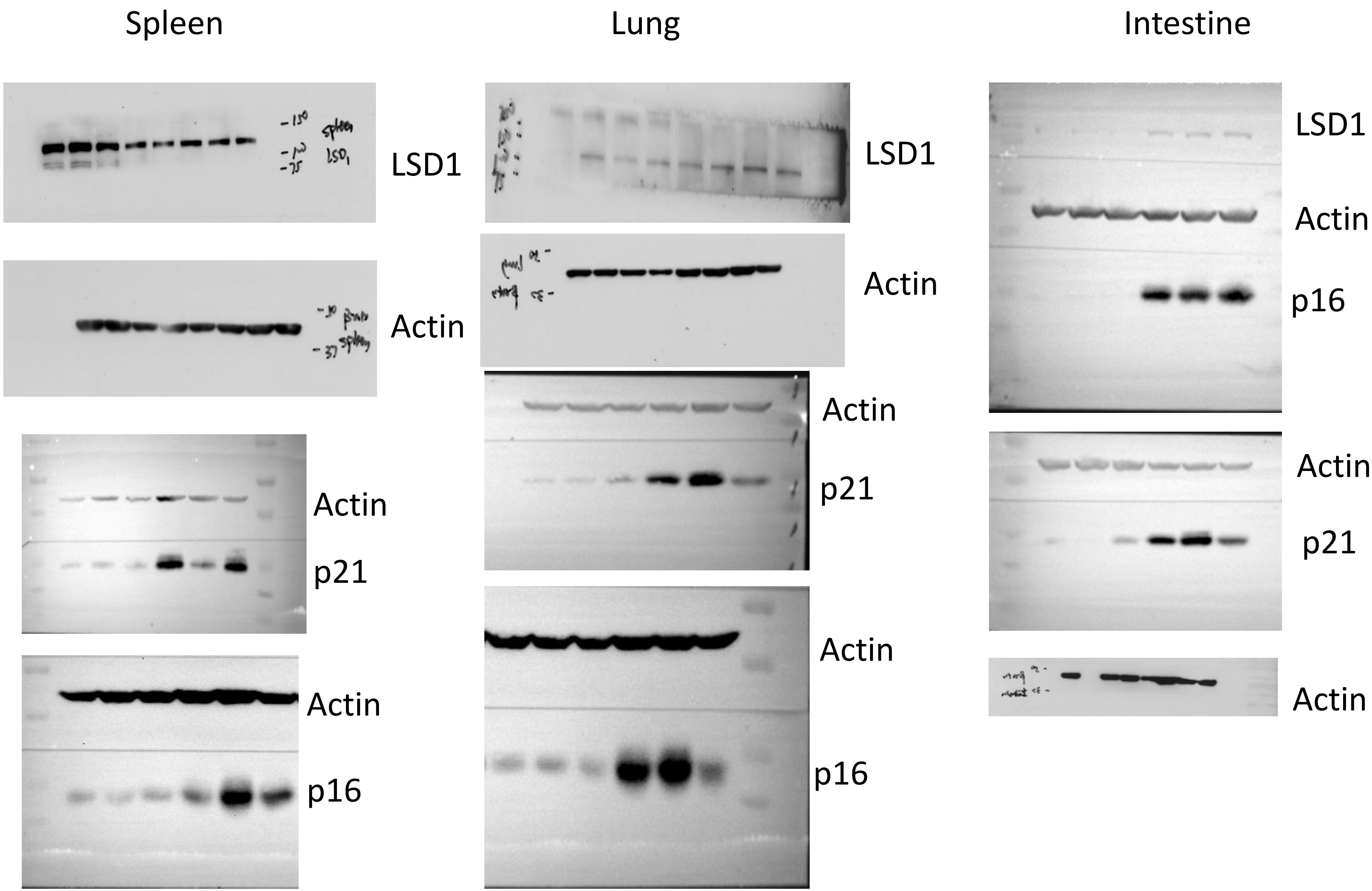
Spleen
Lung
Intestine
LSD1
Actin
Actin
p21
Actin
p16
LSD1
Actin
Actin
p21
Actin
p16
LSD1
Actin
p16
Actin
p21
Actin

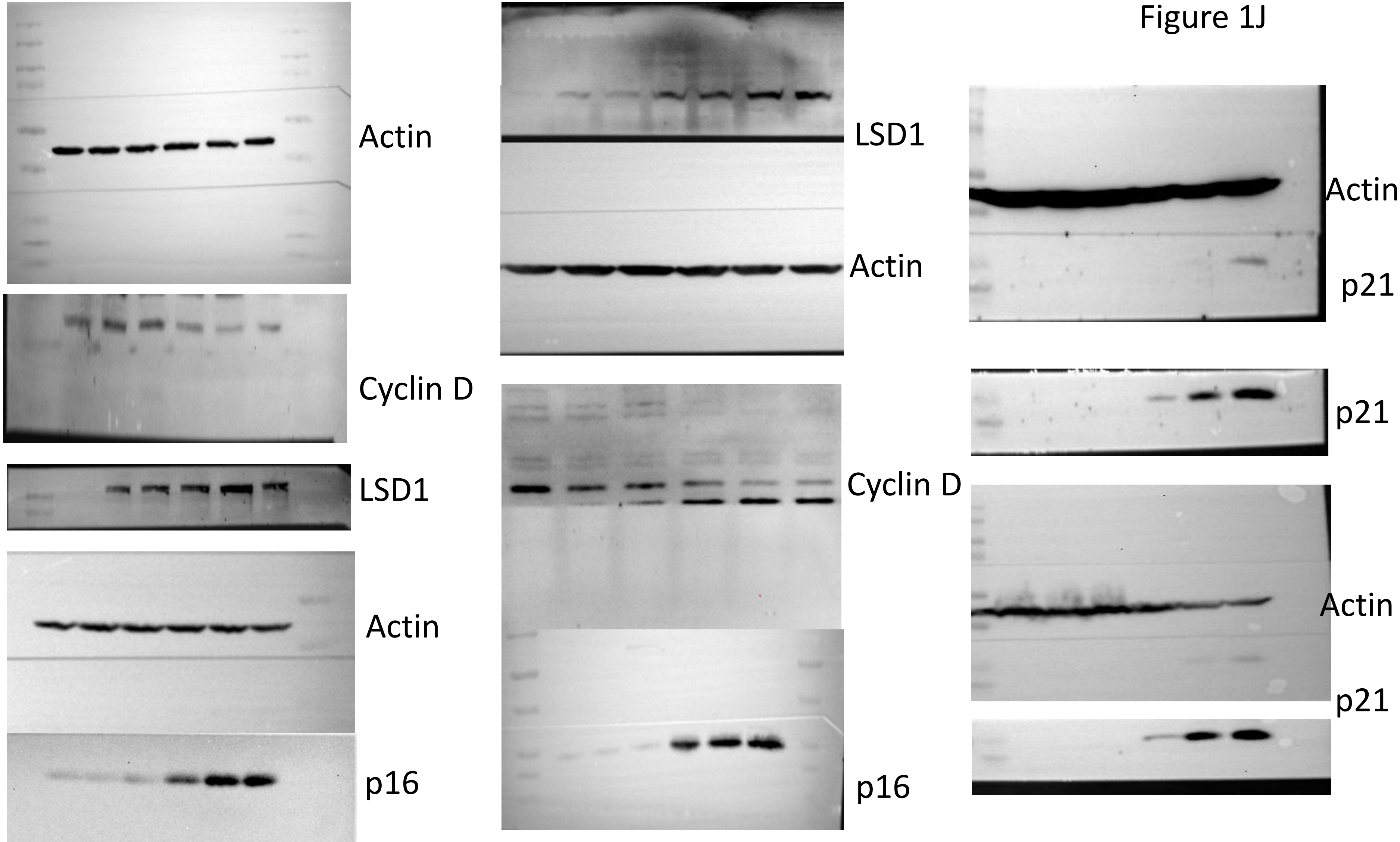
Actin
Cyclin D
LSD1
Actin
p16
LSD1
Actin
Cyclin D
p16
Actin
p21
p21
Actin
p21

Figure 1J

Figure 1K

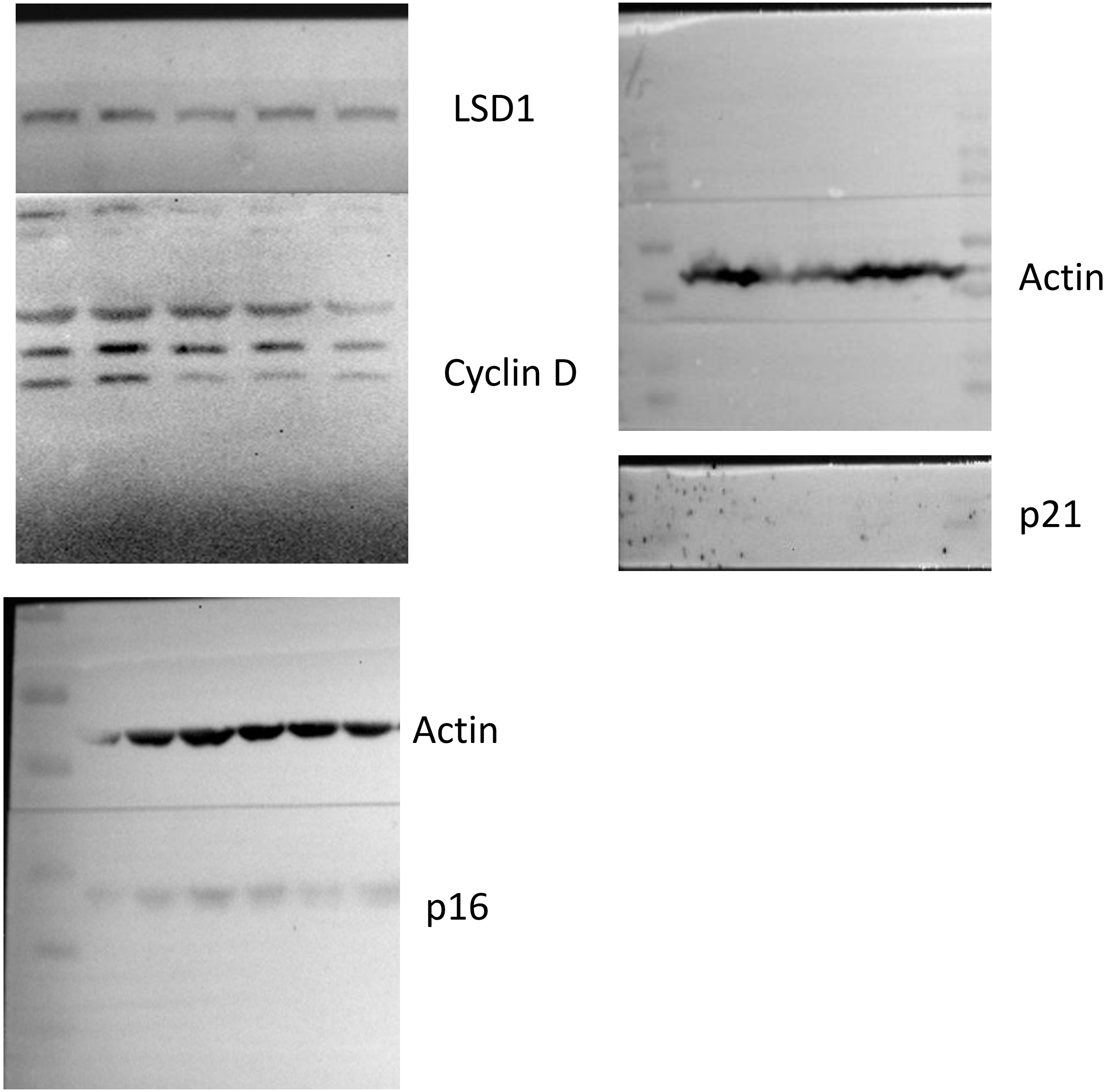

Figure 2A-2C

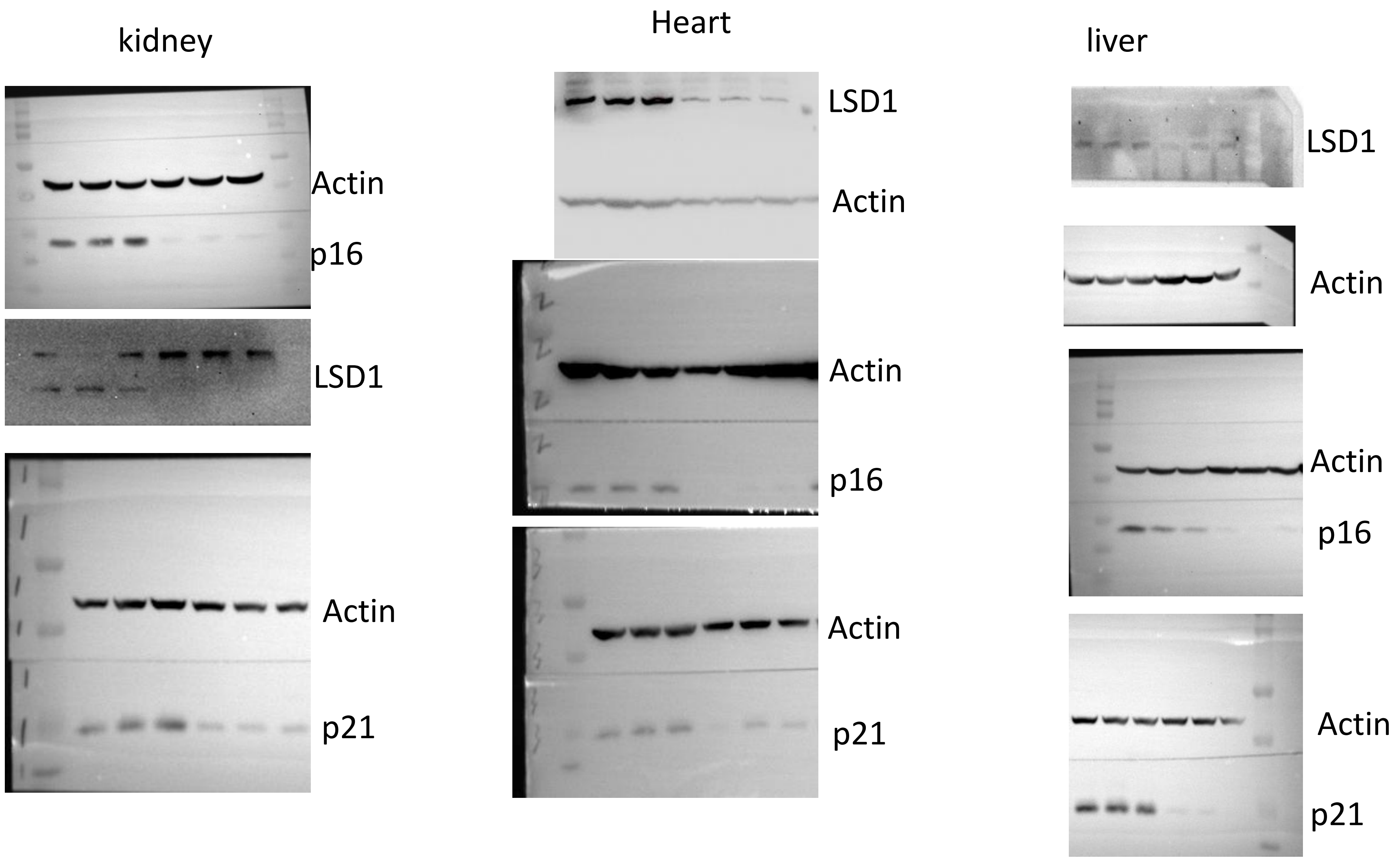
kidney
Actin
p16
LSD1
Actin
p21
Heart
LSD1
Actin
Actin
p16
Actin
p21
liver
LSD1
Actin
Actin
p16
Actin
p21

Figure 2D

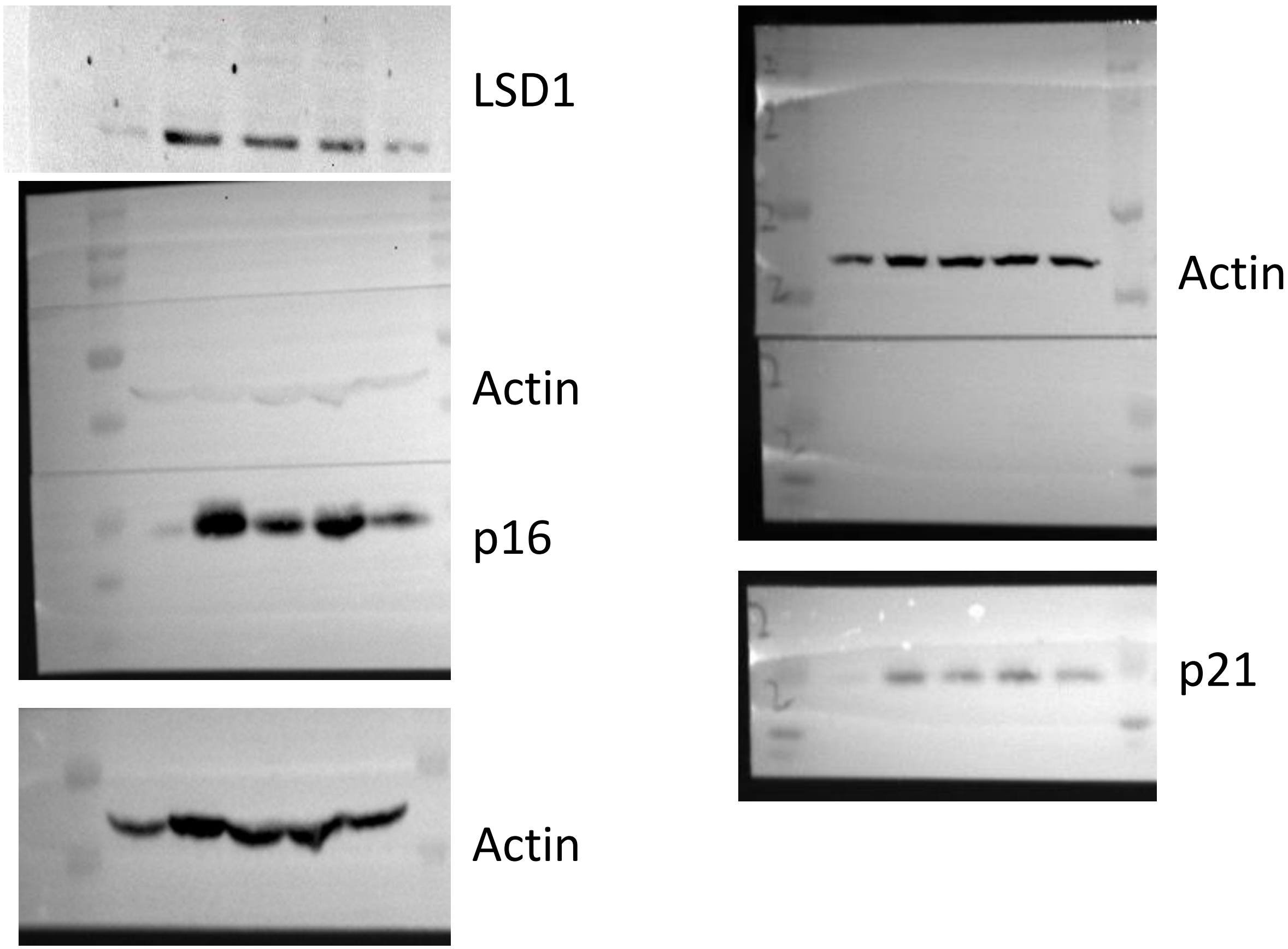

Figure 2G

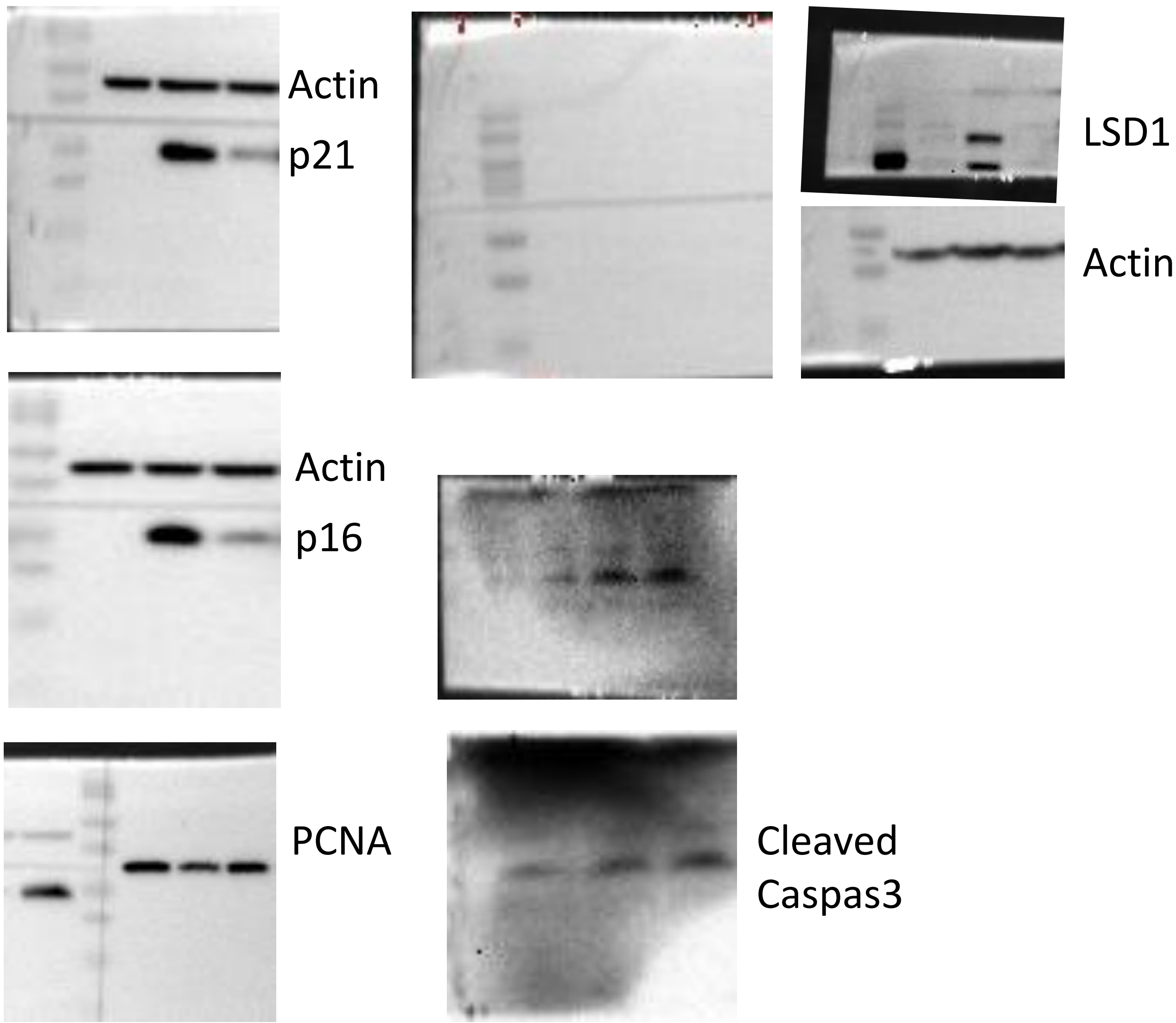

Figure 3C (kidney)

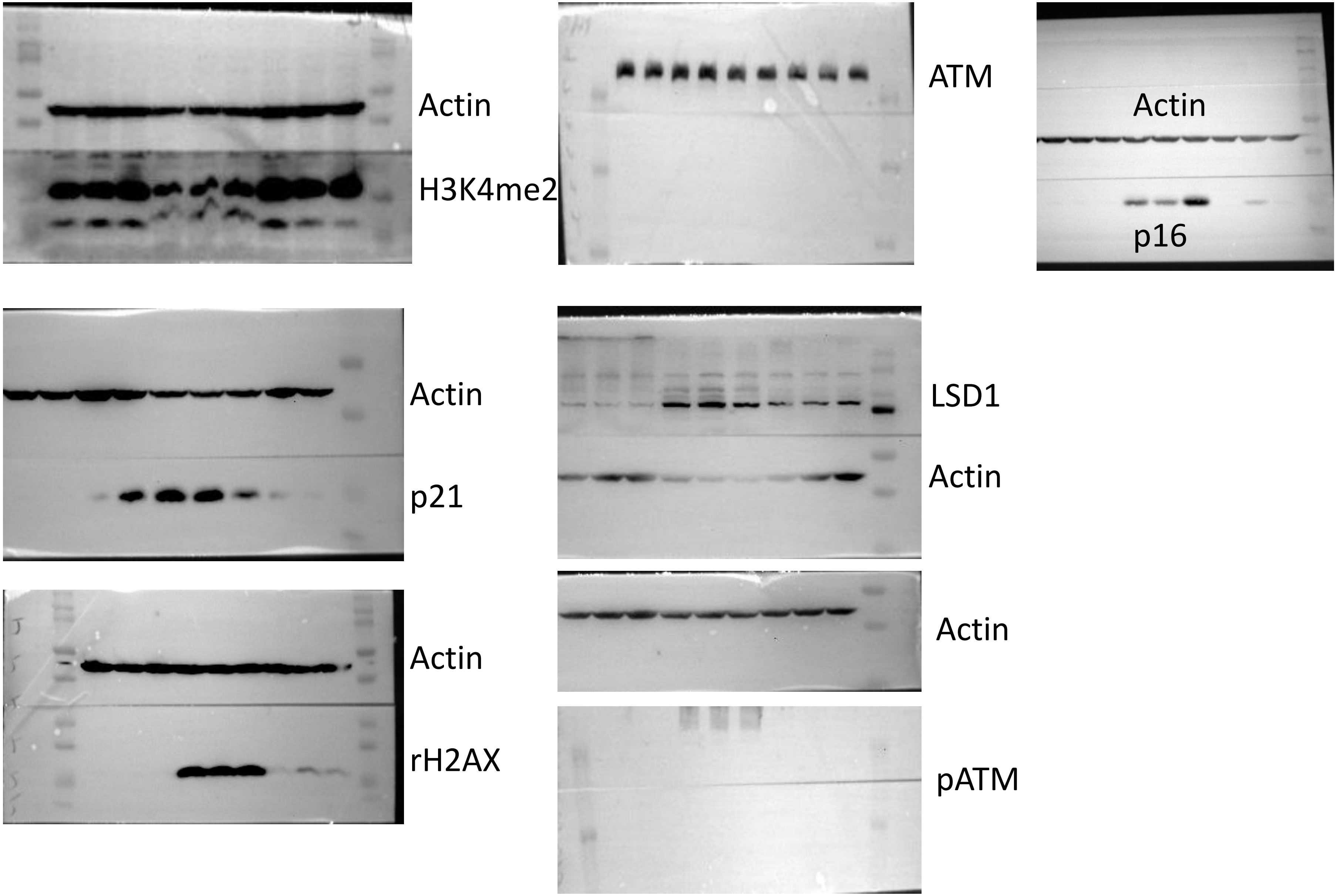

Figure 3C (Heart)

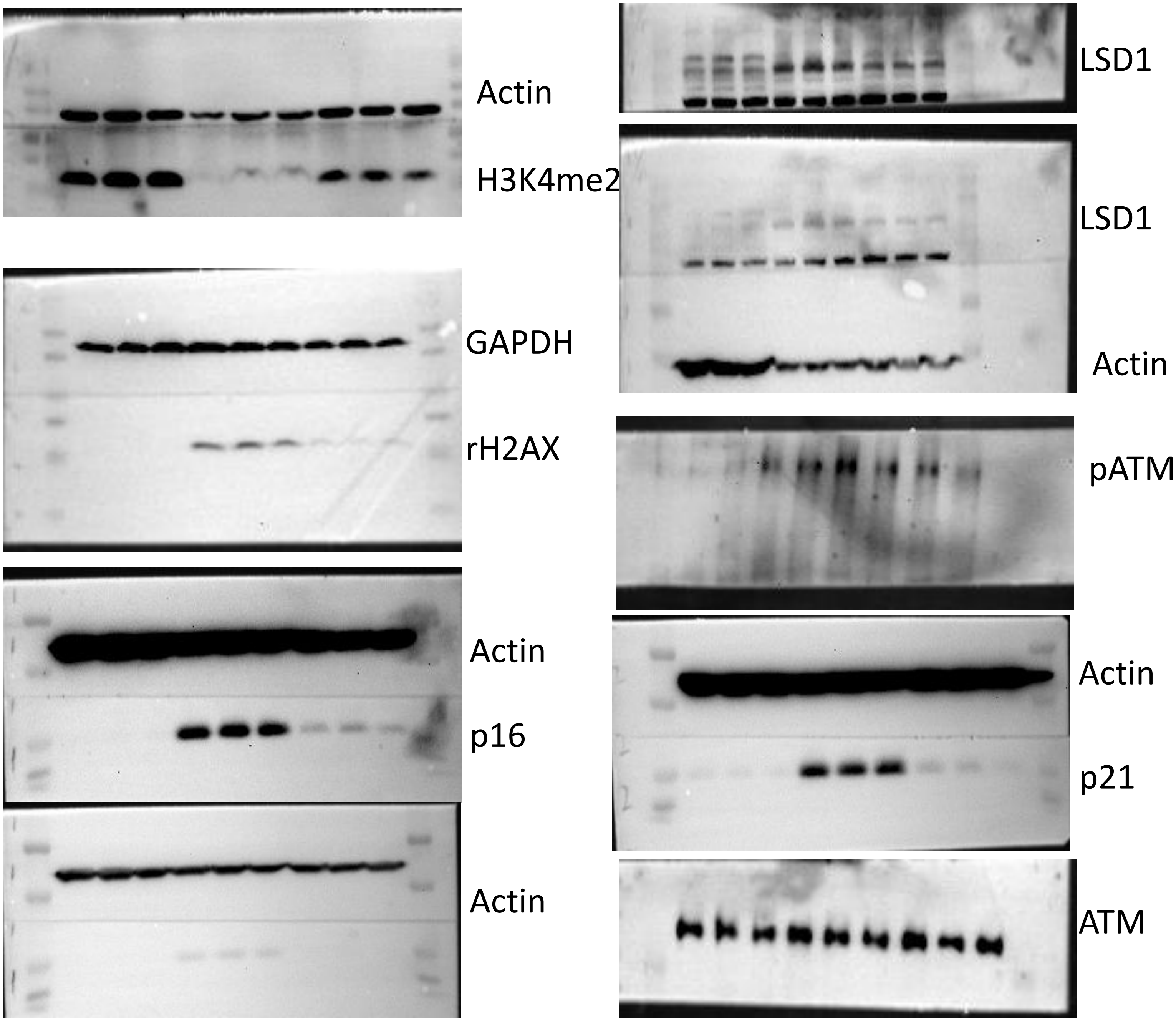

Figure 3C (liver)

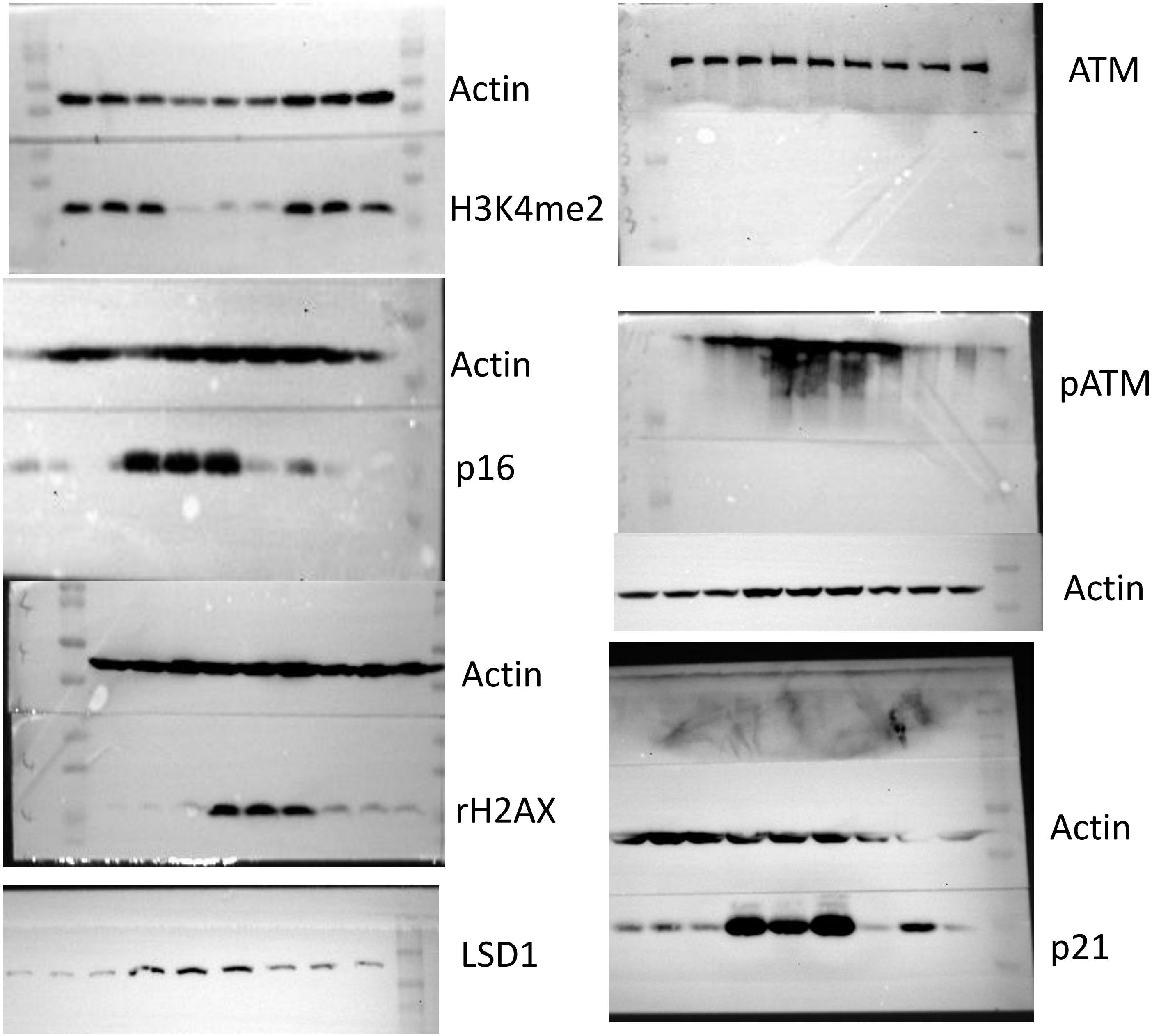

Figure 4A

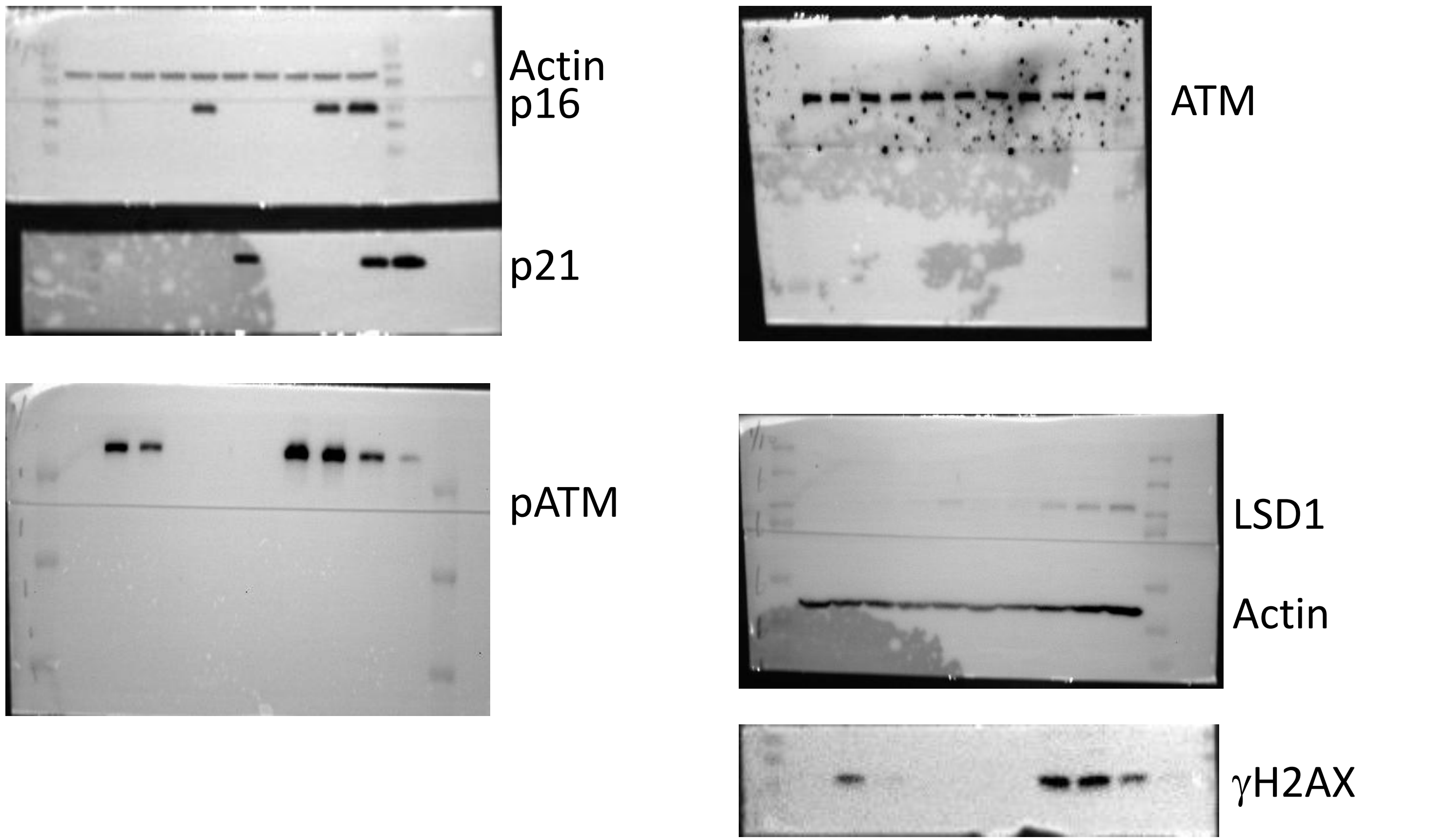

Figure 4B

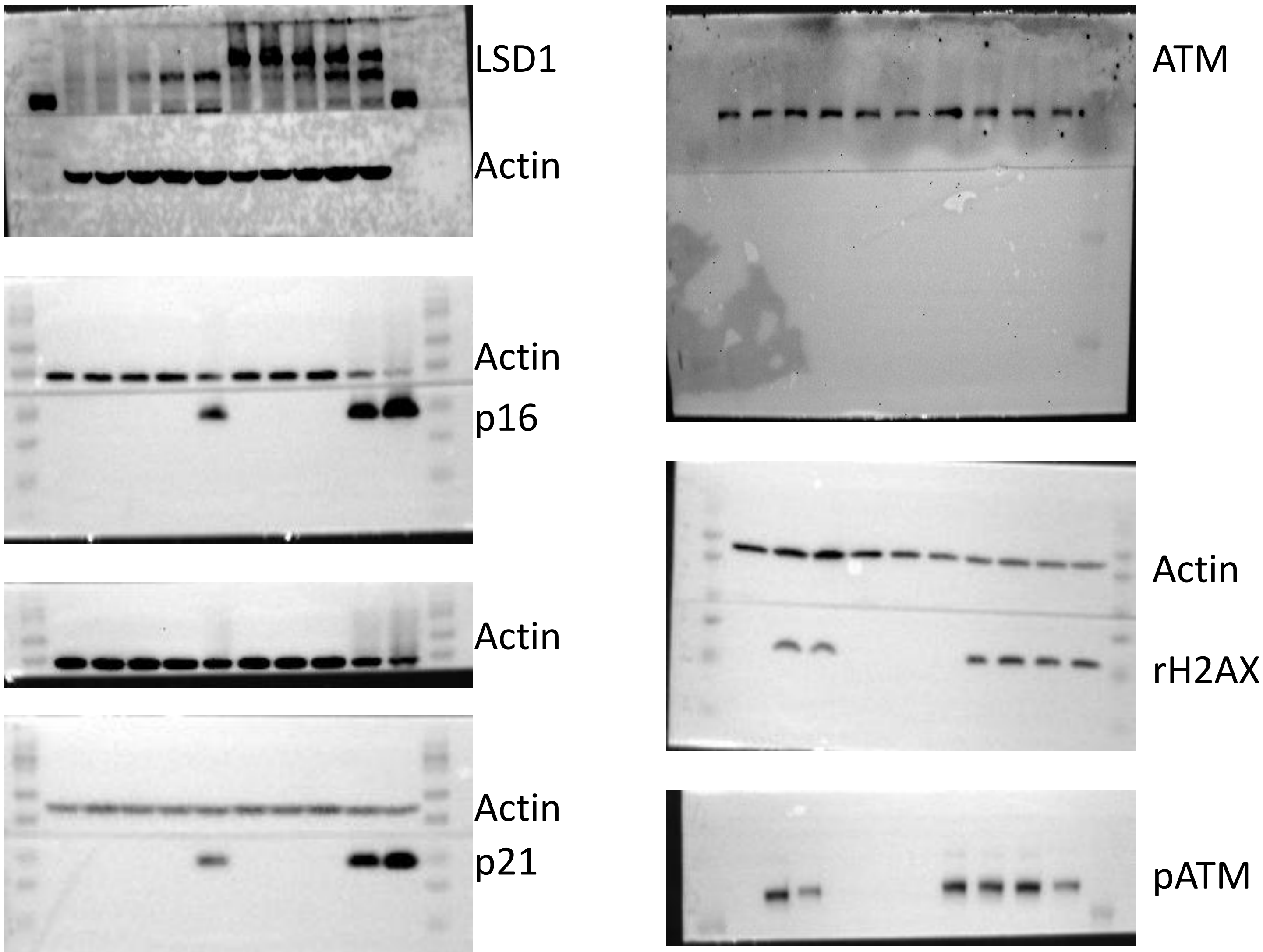

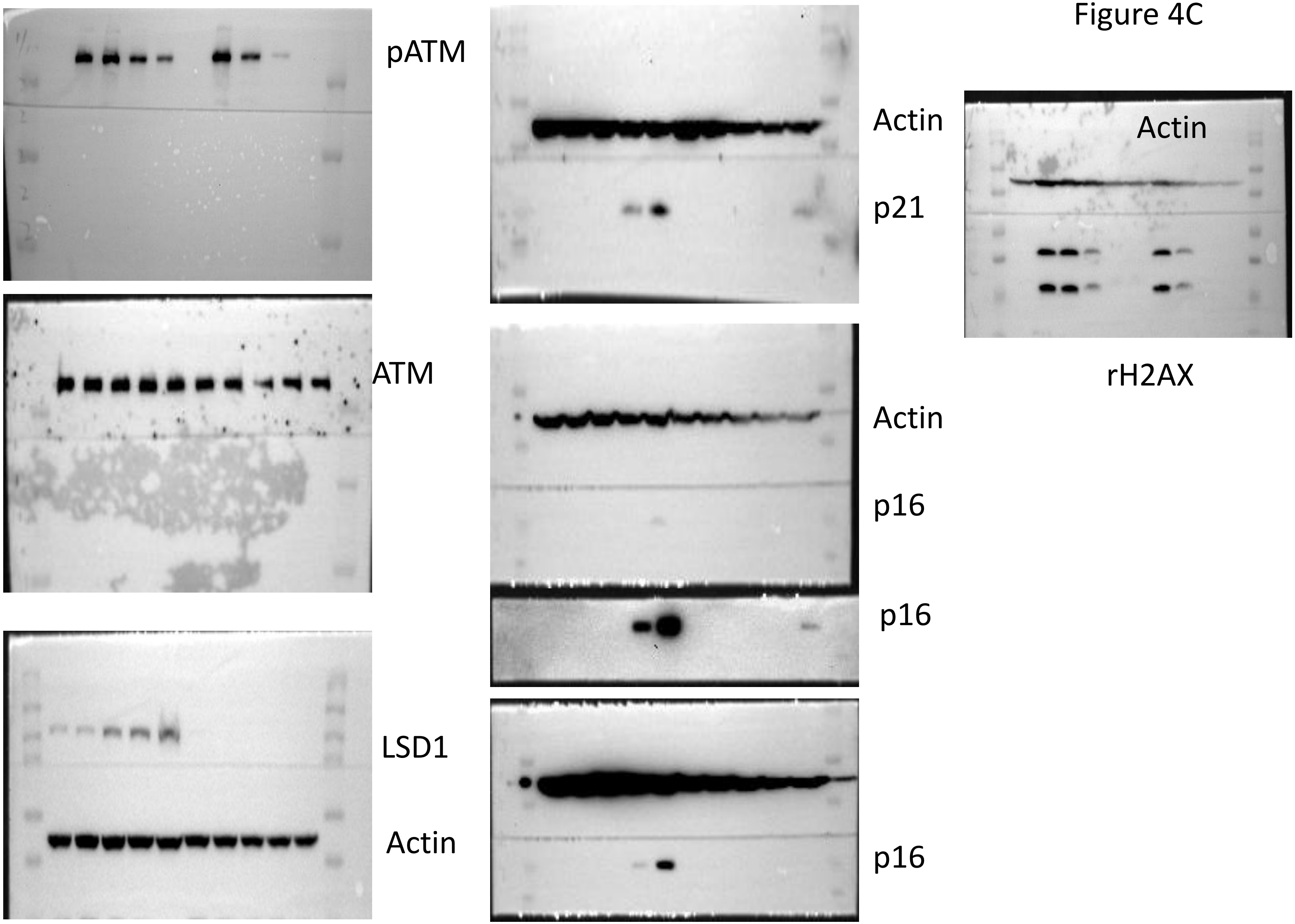

Figure 4C

Figure 5A,5B,5D

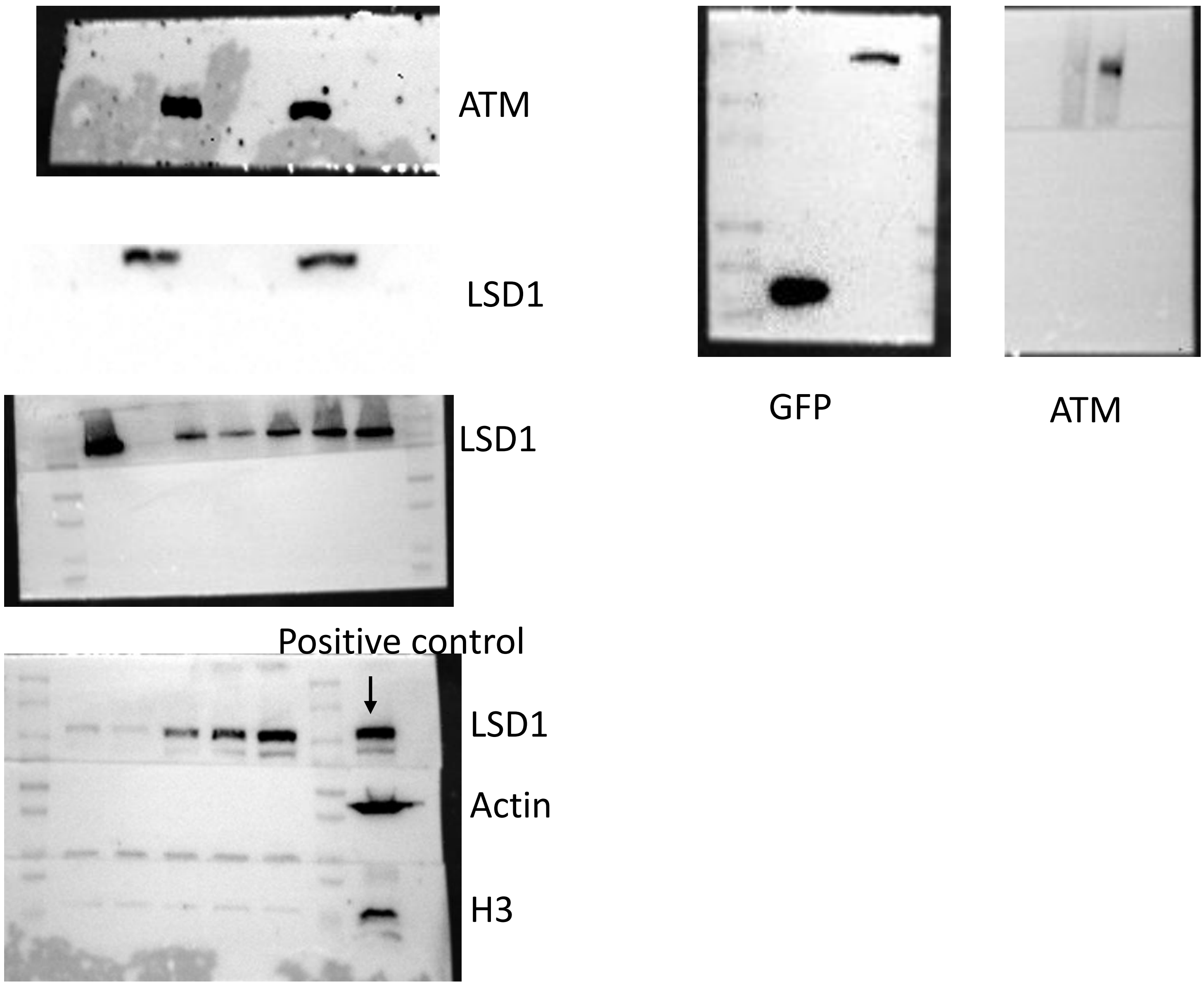

Figure 5C and 5E

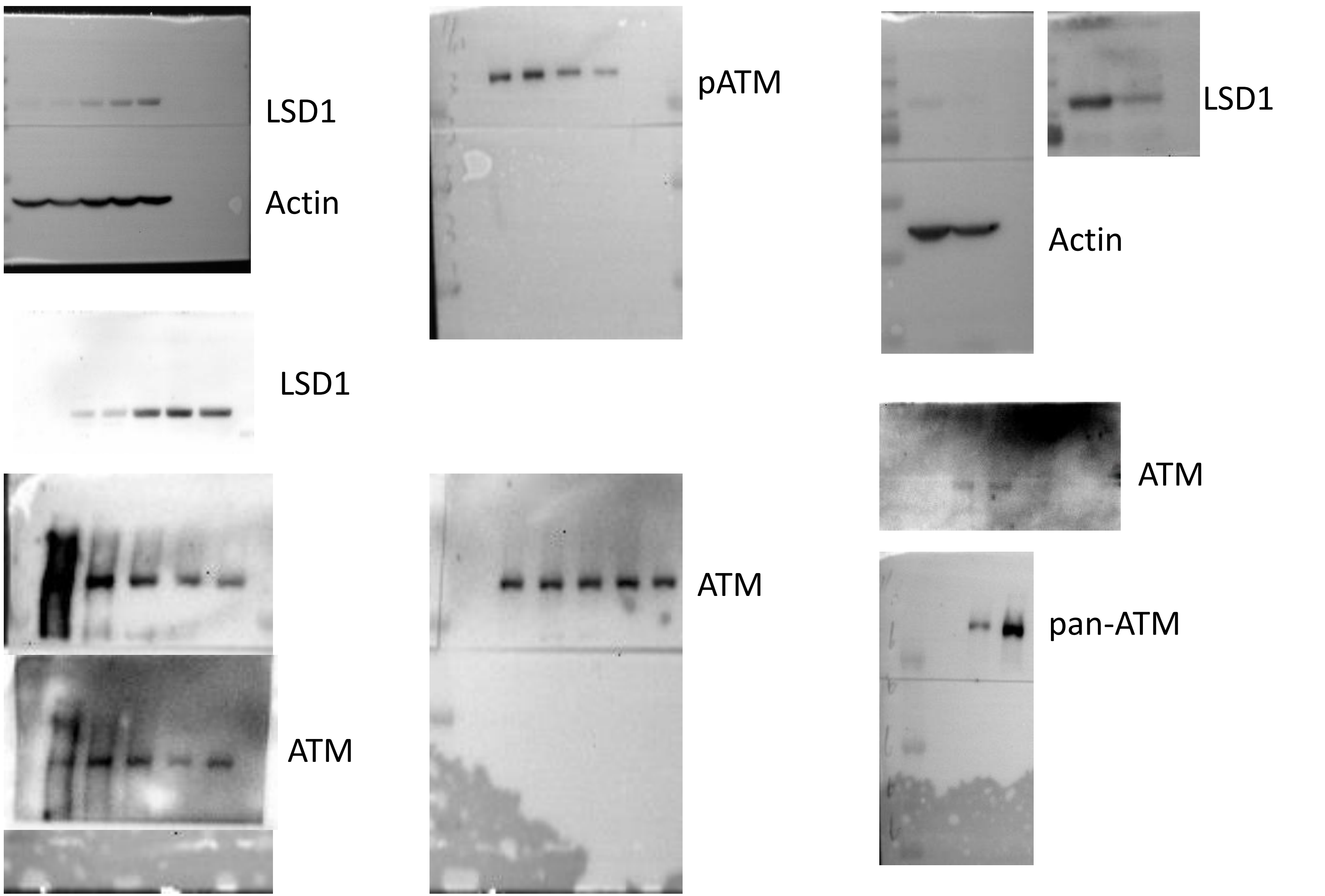

Figure 5F

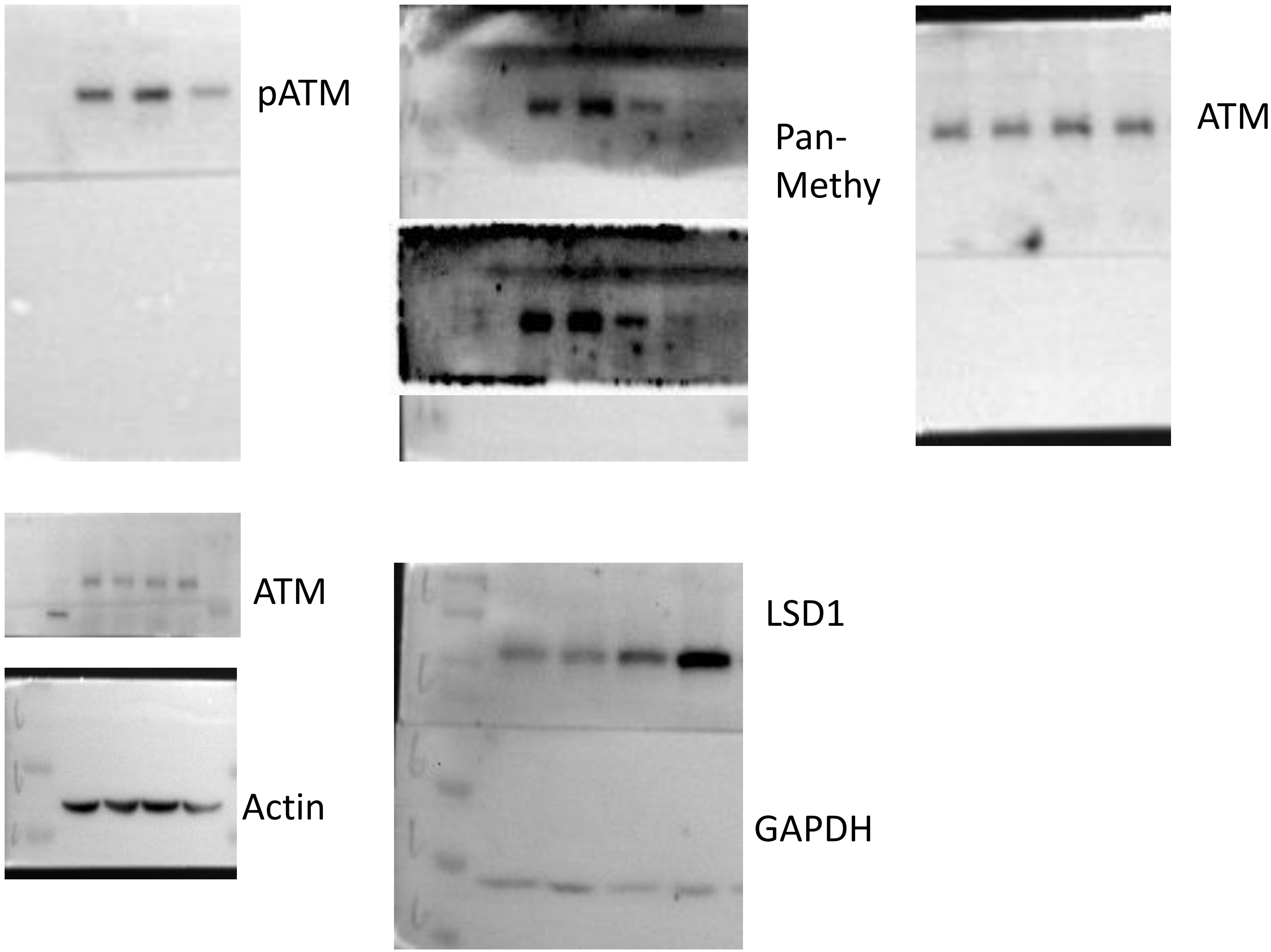

Figure 5G

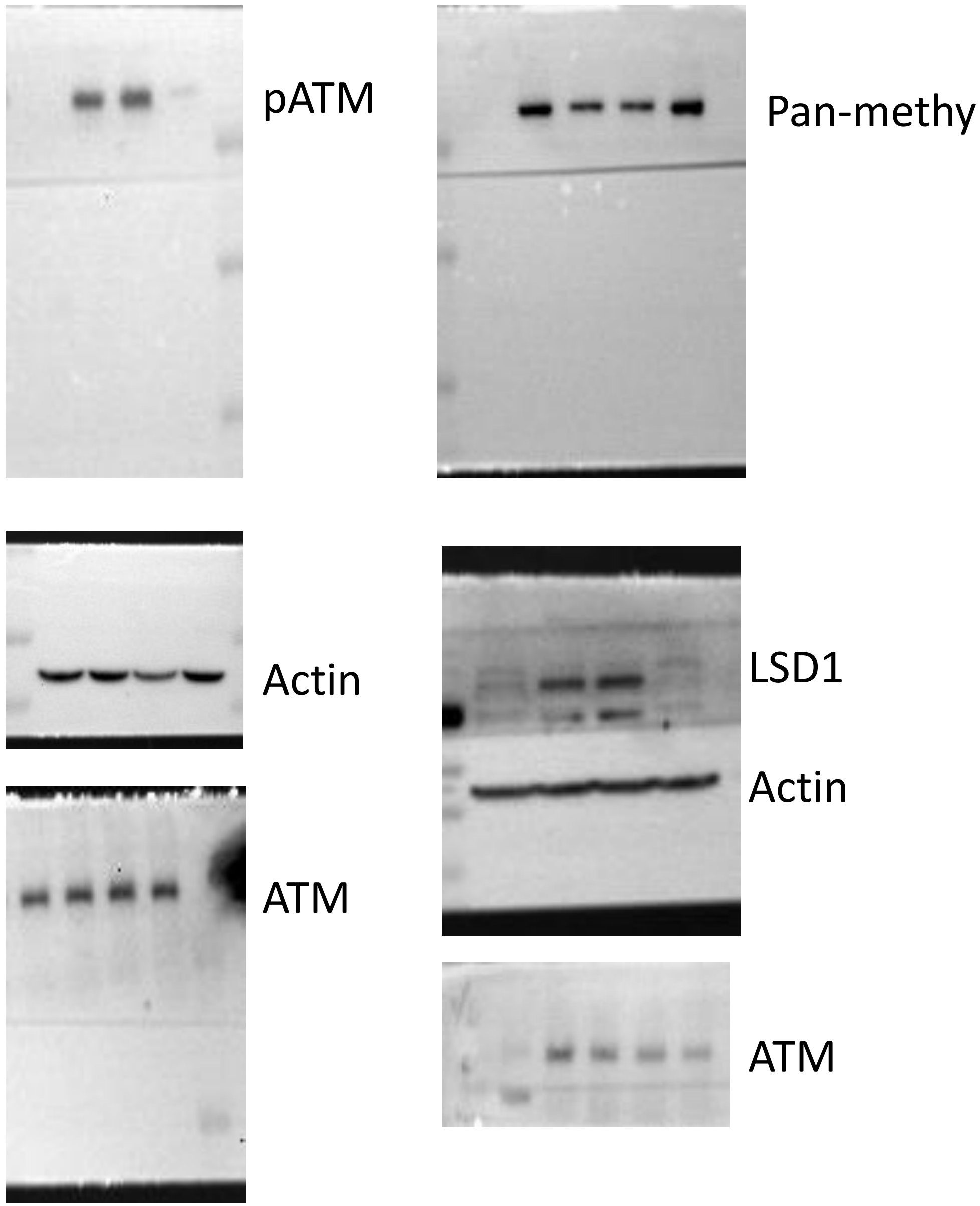

Figure 6A

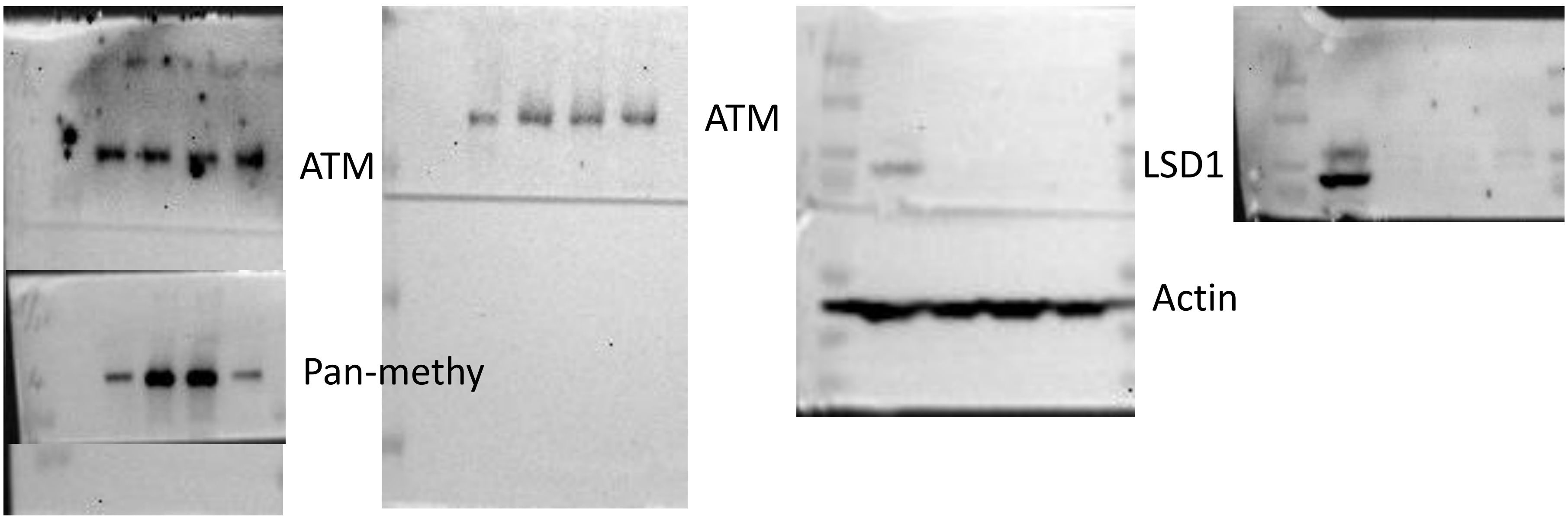

Figure 6B and 6C

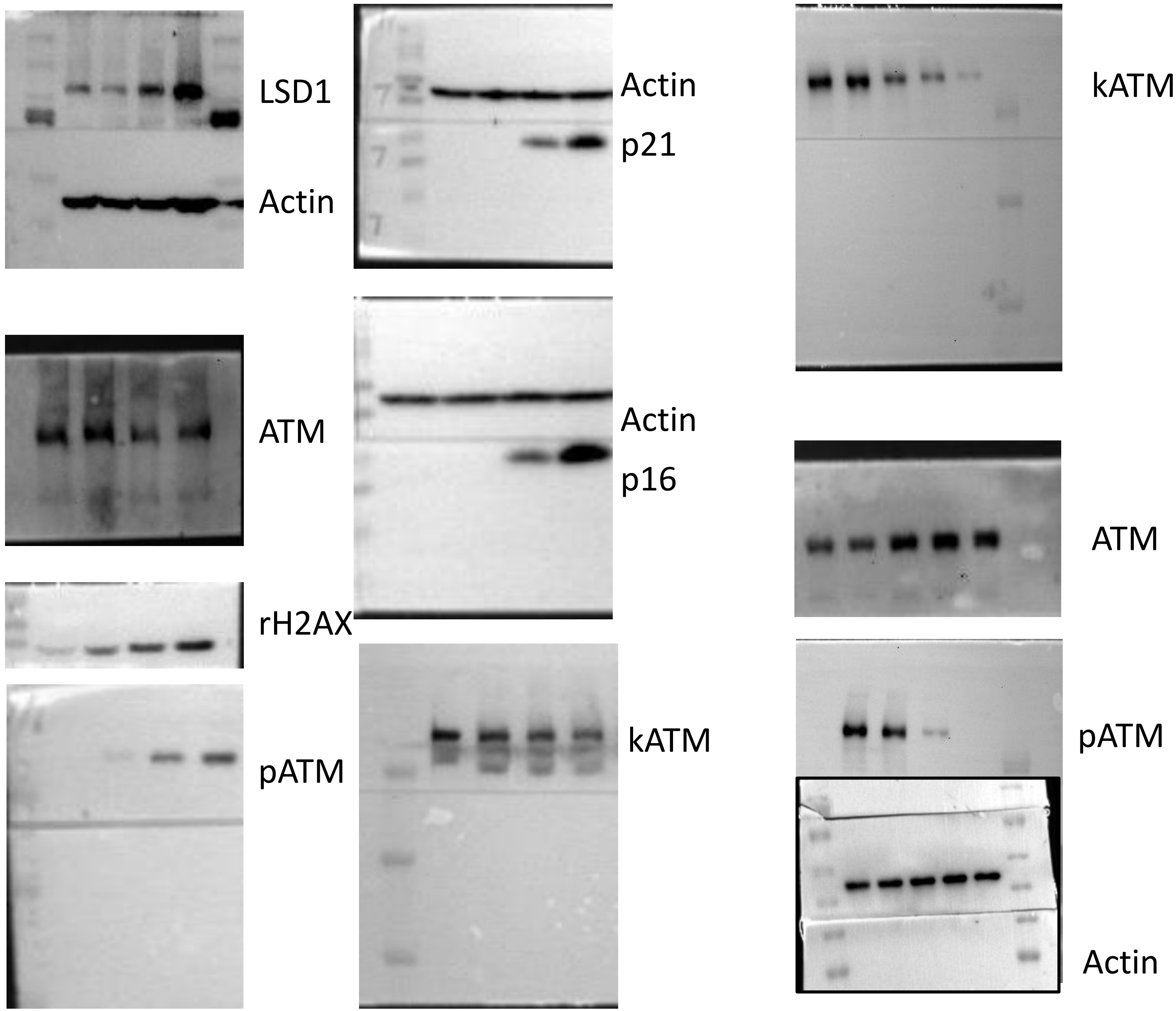

Figure 6D and 6F

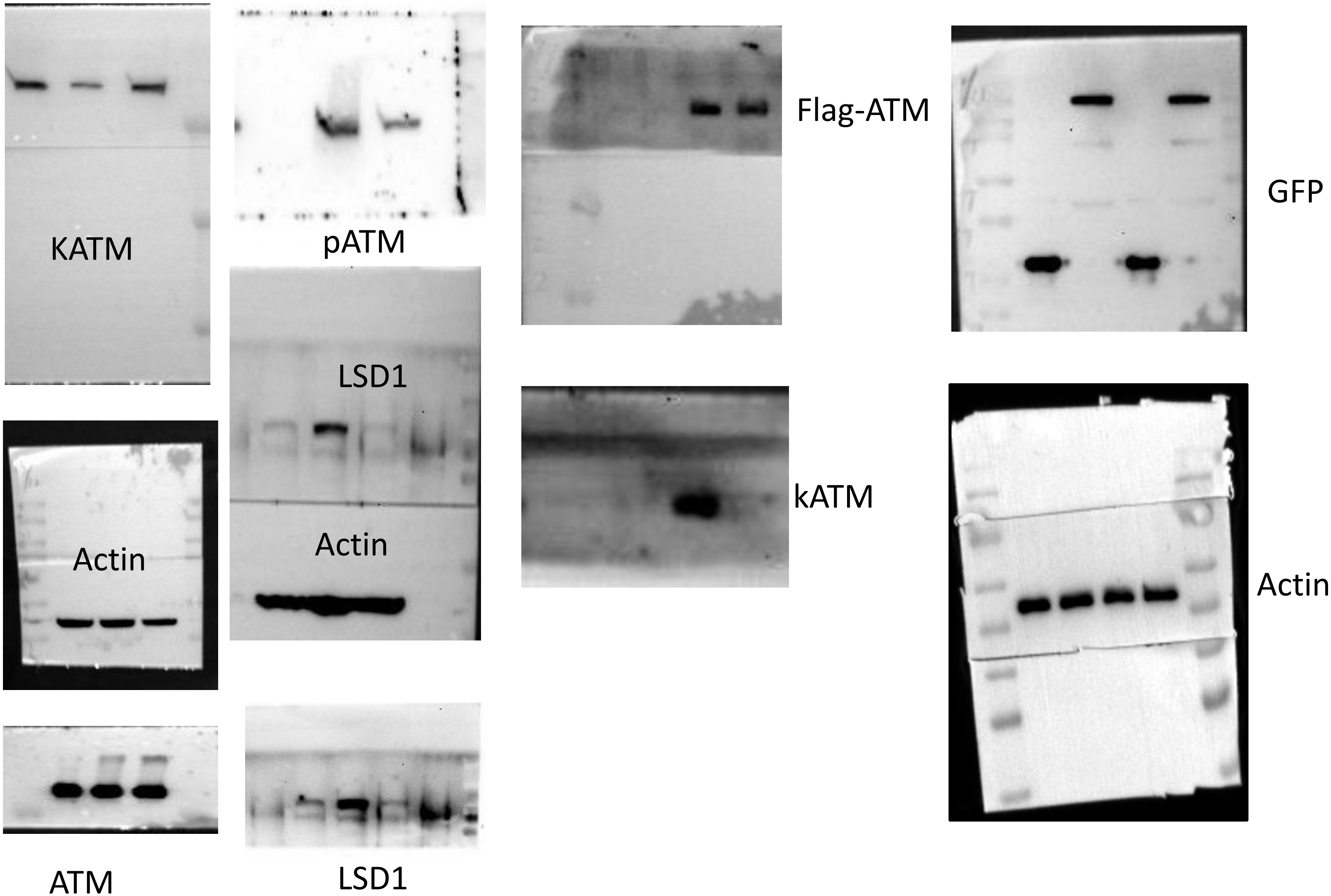

Figure 6G

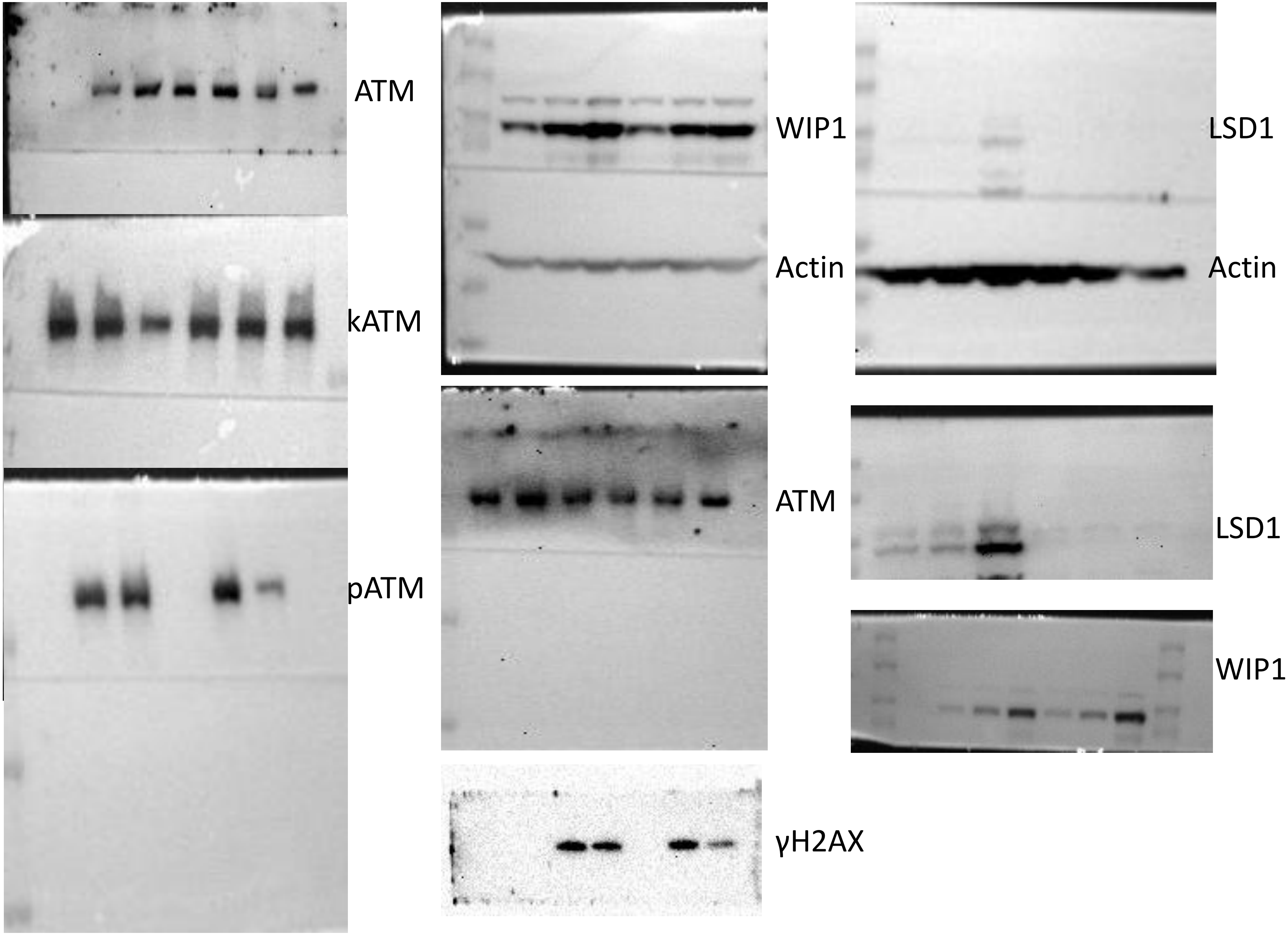

Figure 6H

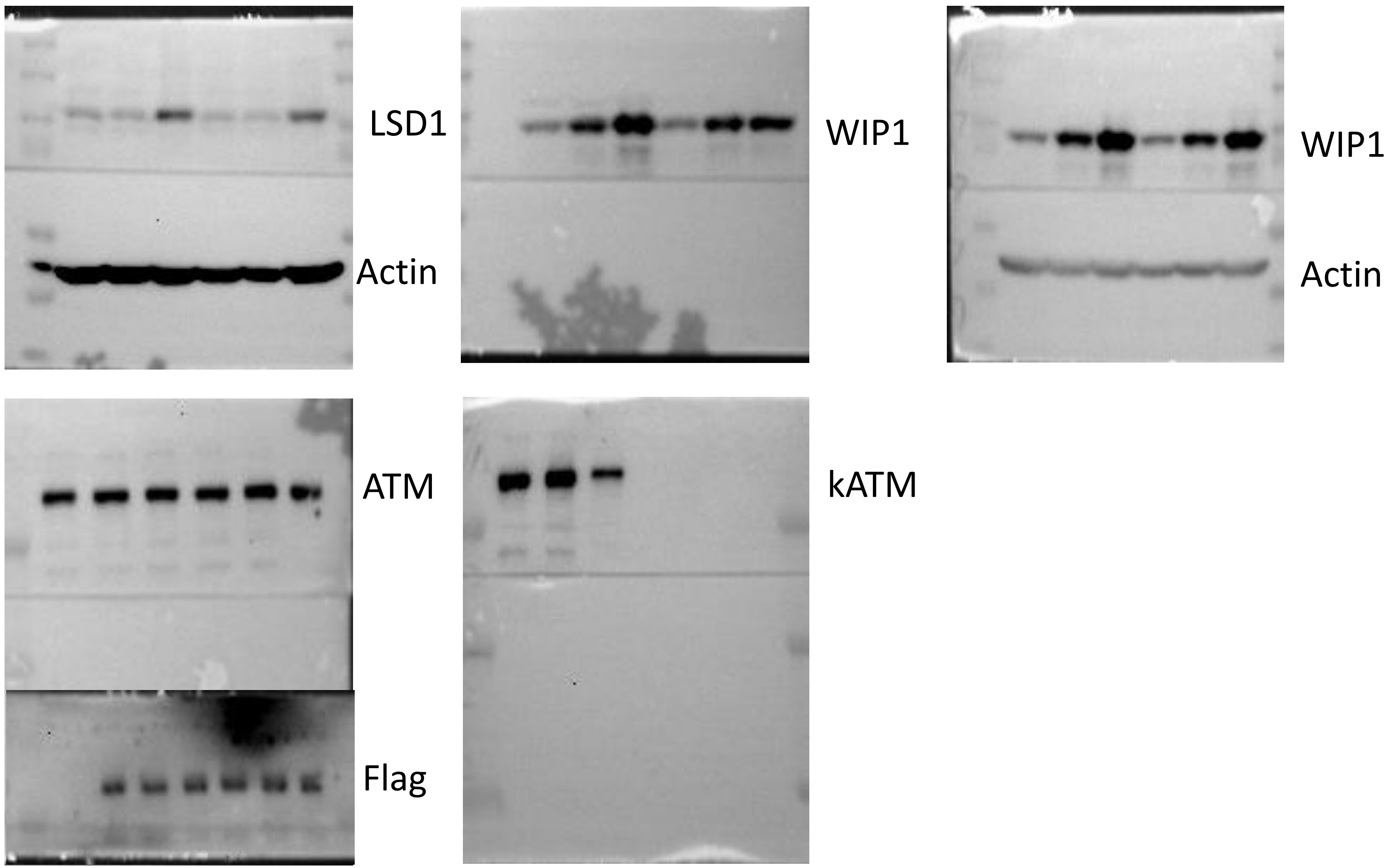

Figure 7A -7C,7E

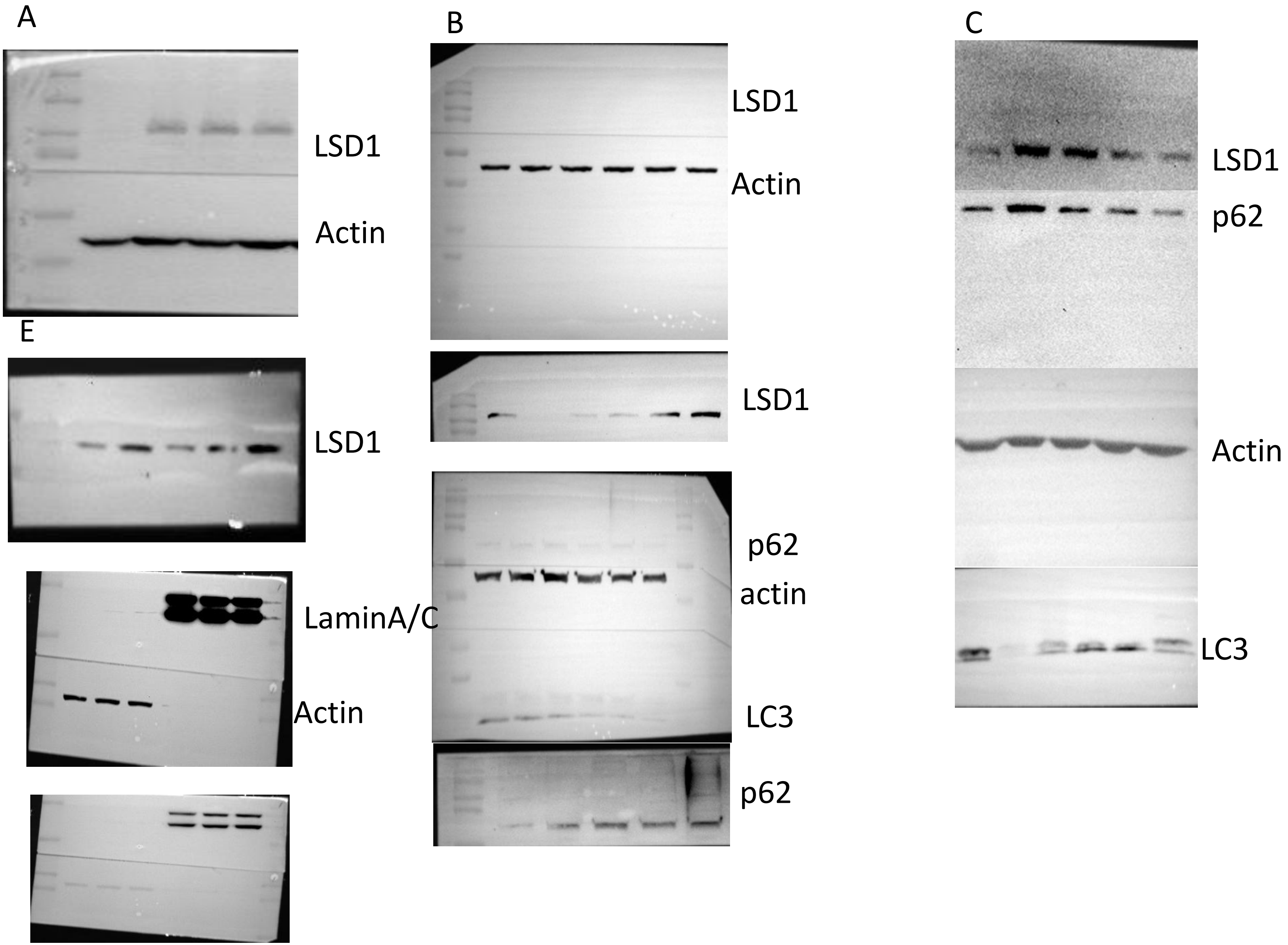

Figure 7G

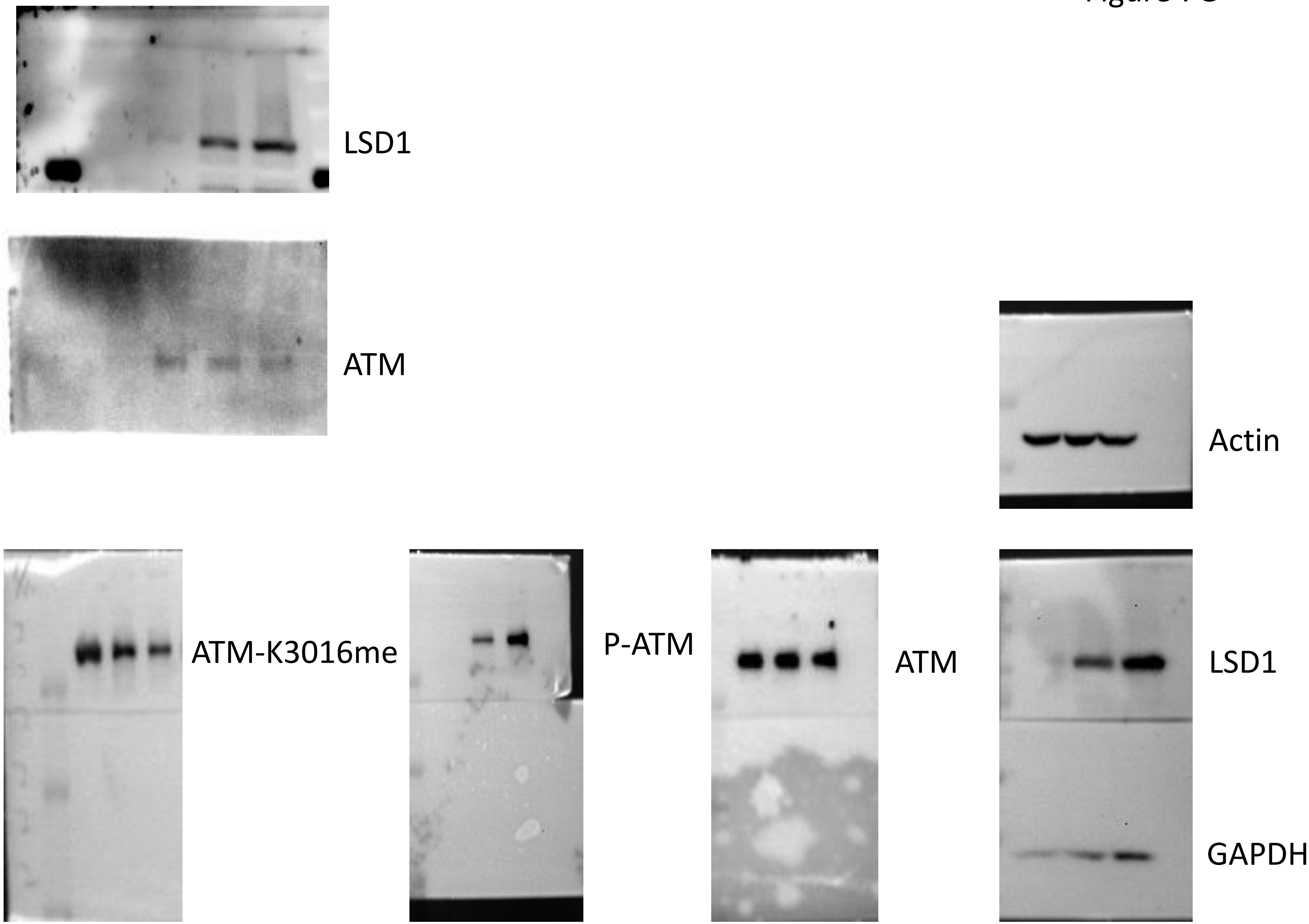

Figure 7H

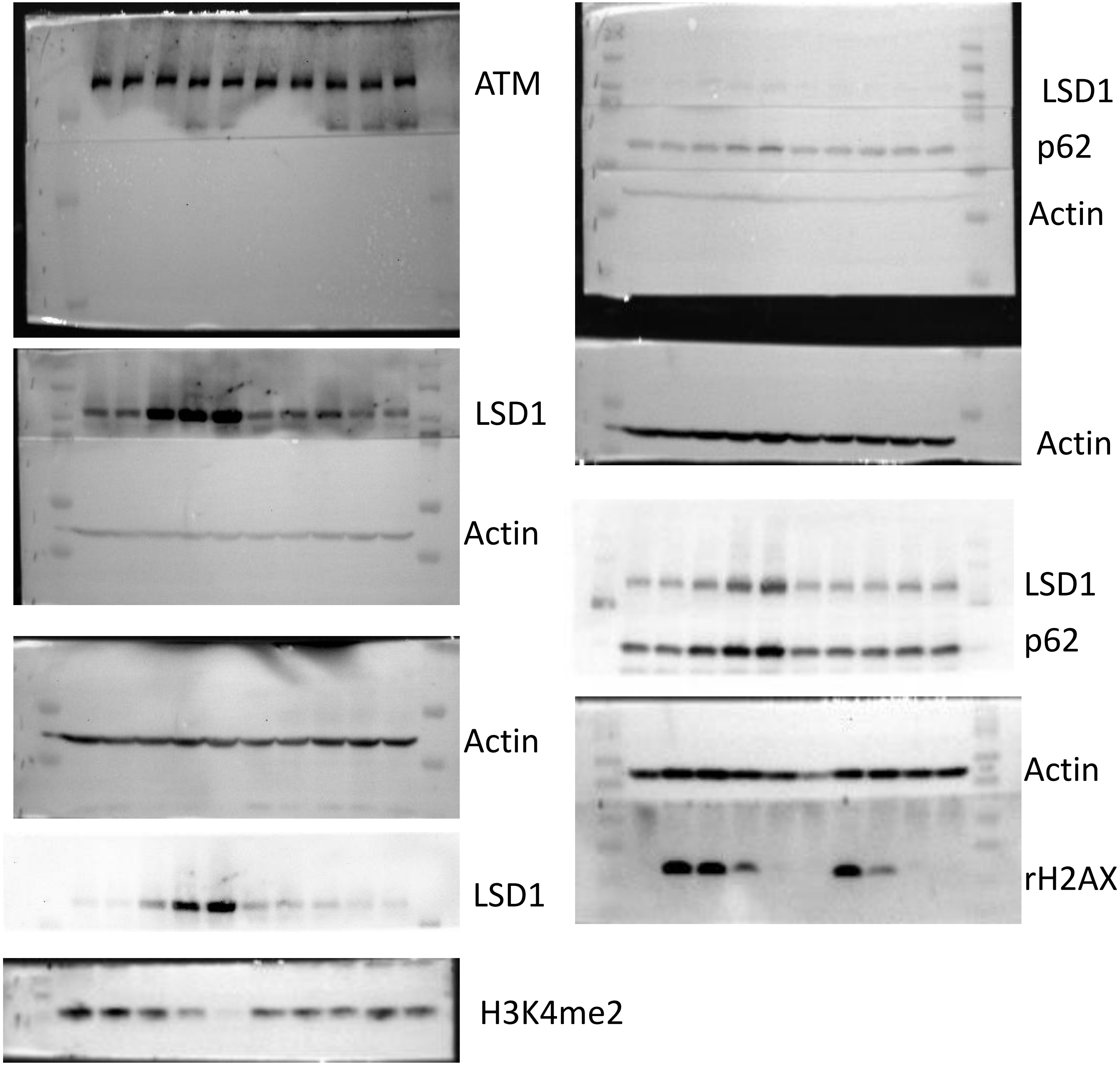
ATM
LSD1
Actin
Actin
LSD1
H3K4me2
LSD1
p62
Actin
Actin
LSD1
p62
Actin
rH2AX

Figure 7H

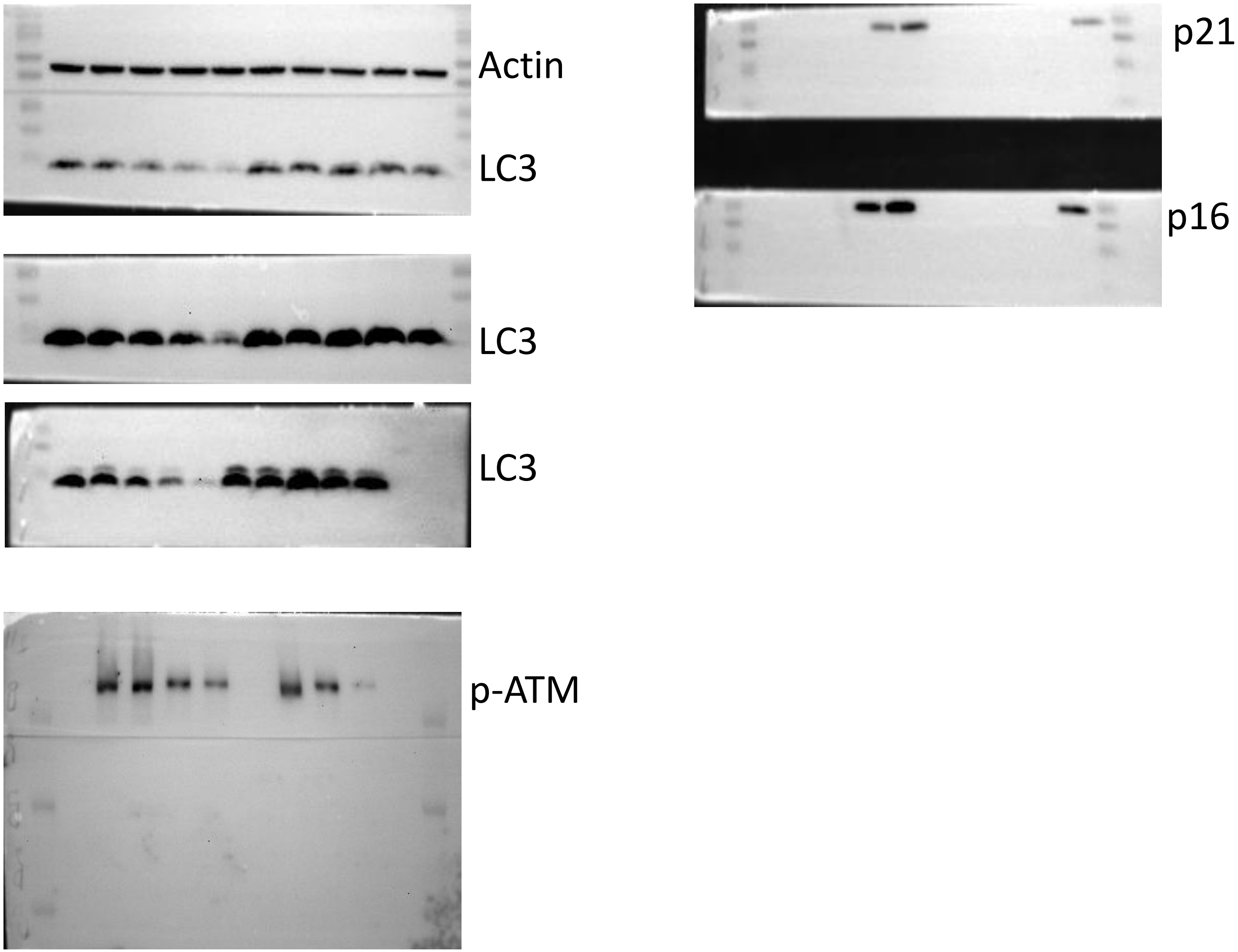

Figure 8A-8C

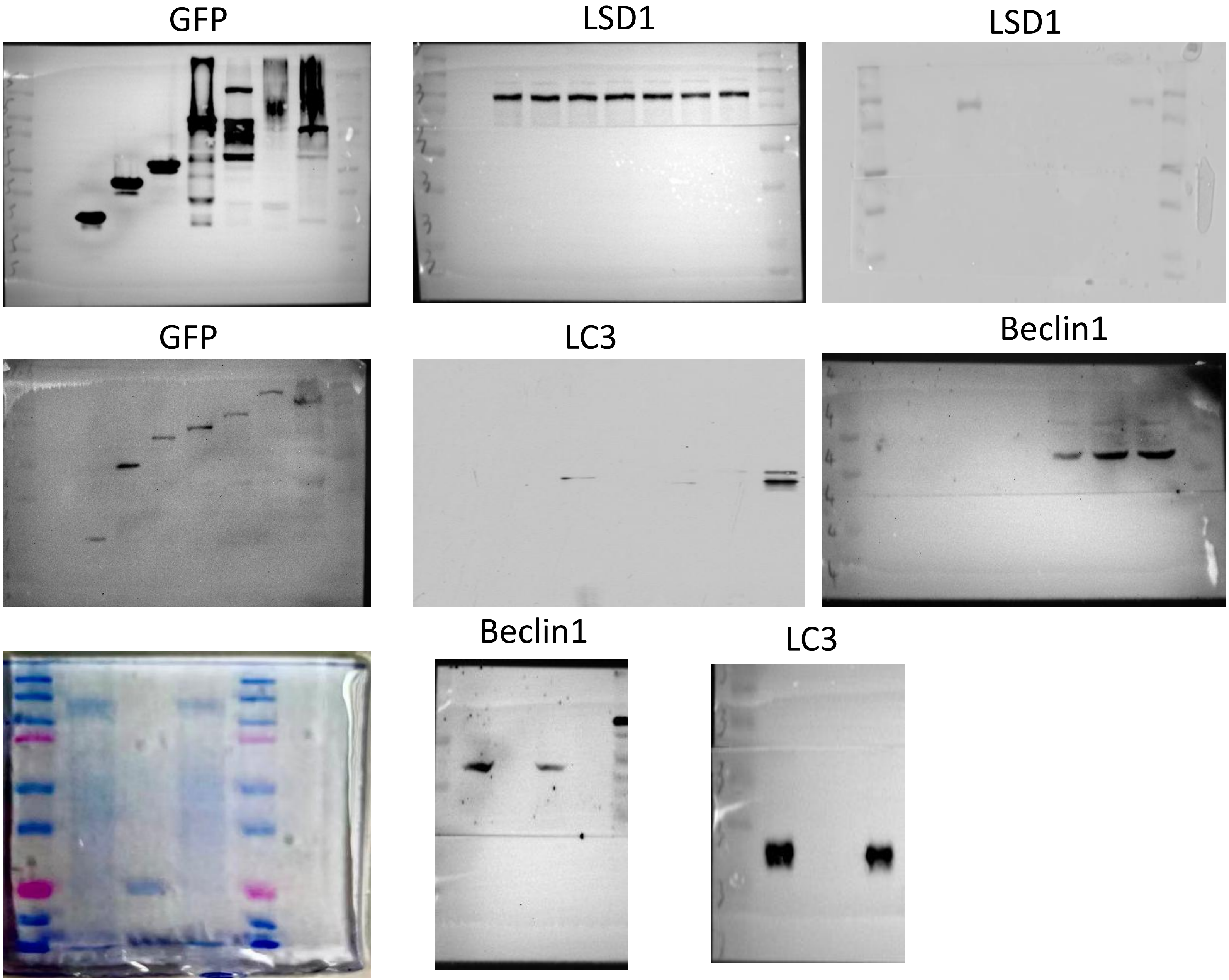

Figure 8D-8F

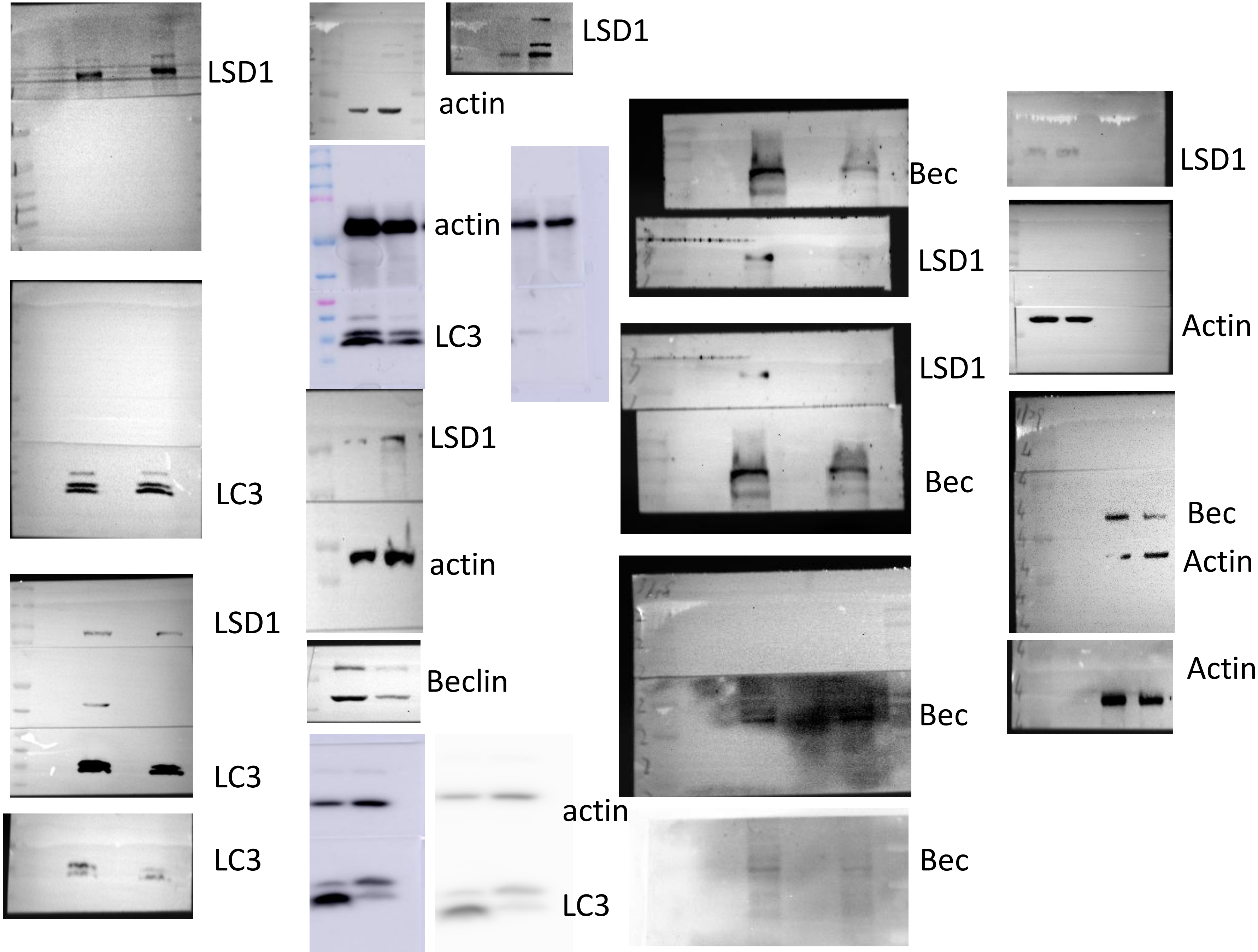
LSD1
actin
LSD1
actin
LC3
LSD1
actin
Beclin
actin
LC3
LC3
LSD1
LC3
LC3
Bec
LSD1
LSD1
Bec
Bec
Bec
LSD1
Actin
Bec
Actin
Actin

Figure 8G-8H

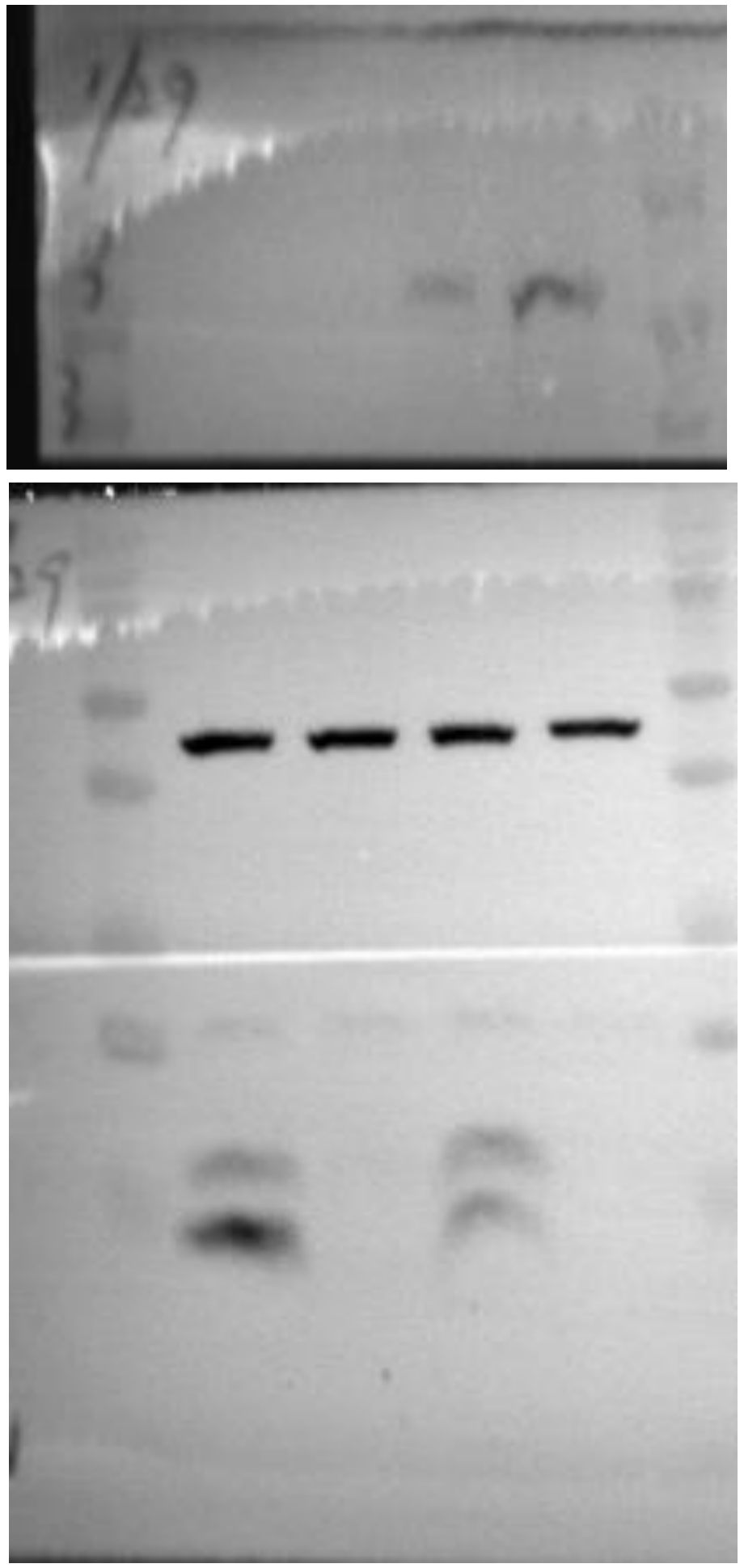

LSD1

Actin

LC3

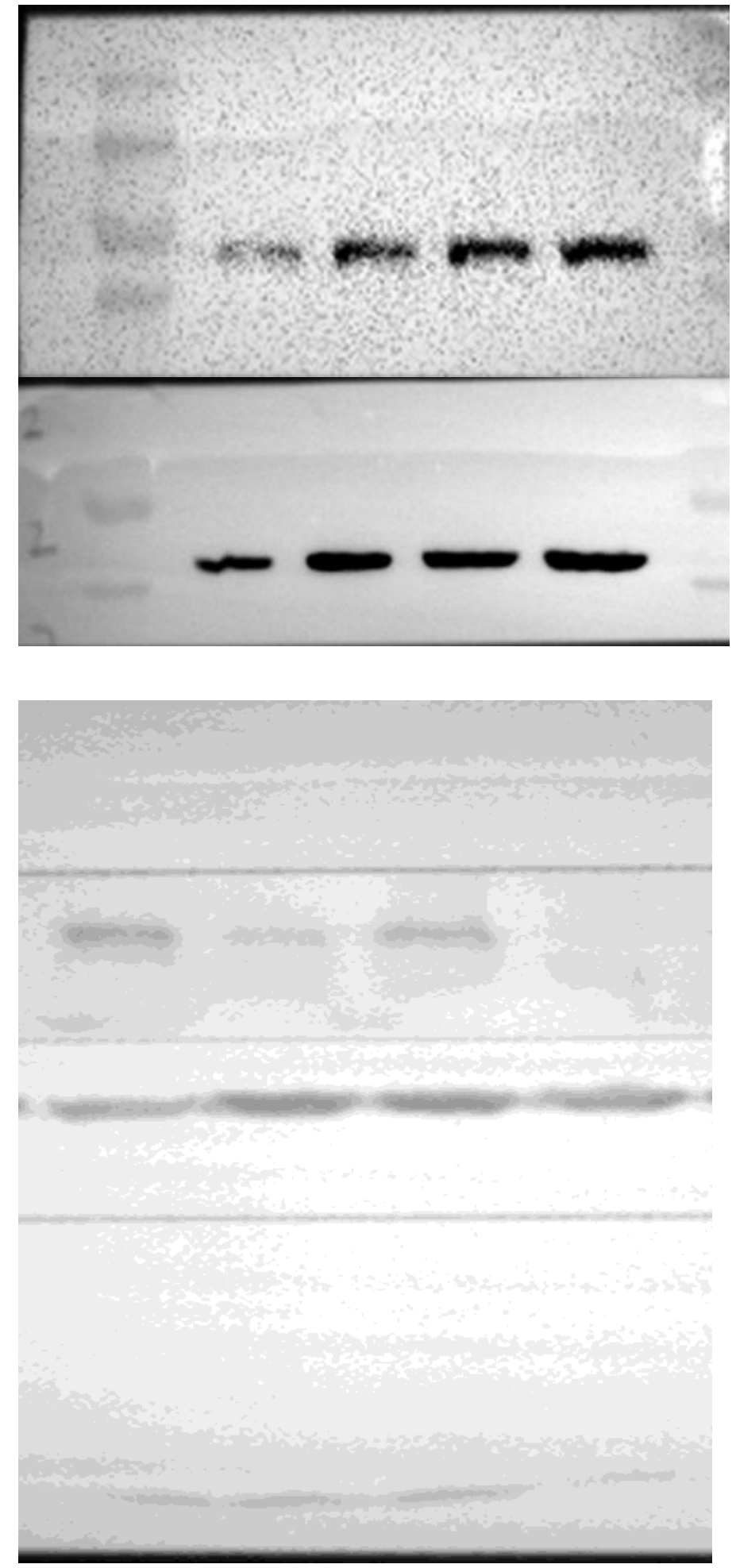

LSD1

Actin

Beclin1

Actin

Sup Figure 1A

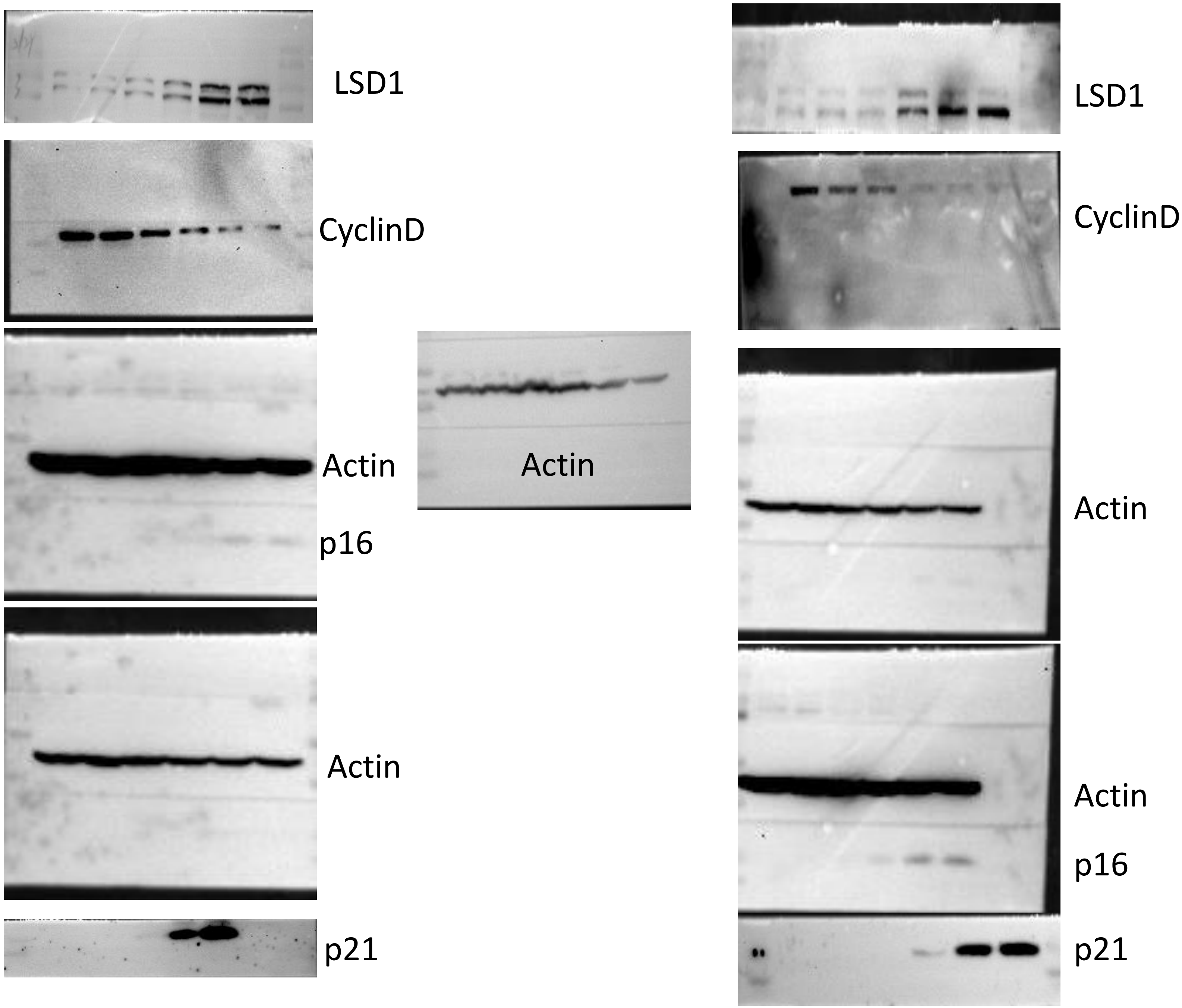

Sup Figure 1B

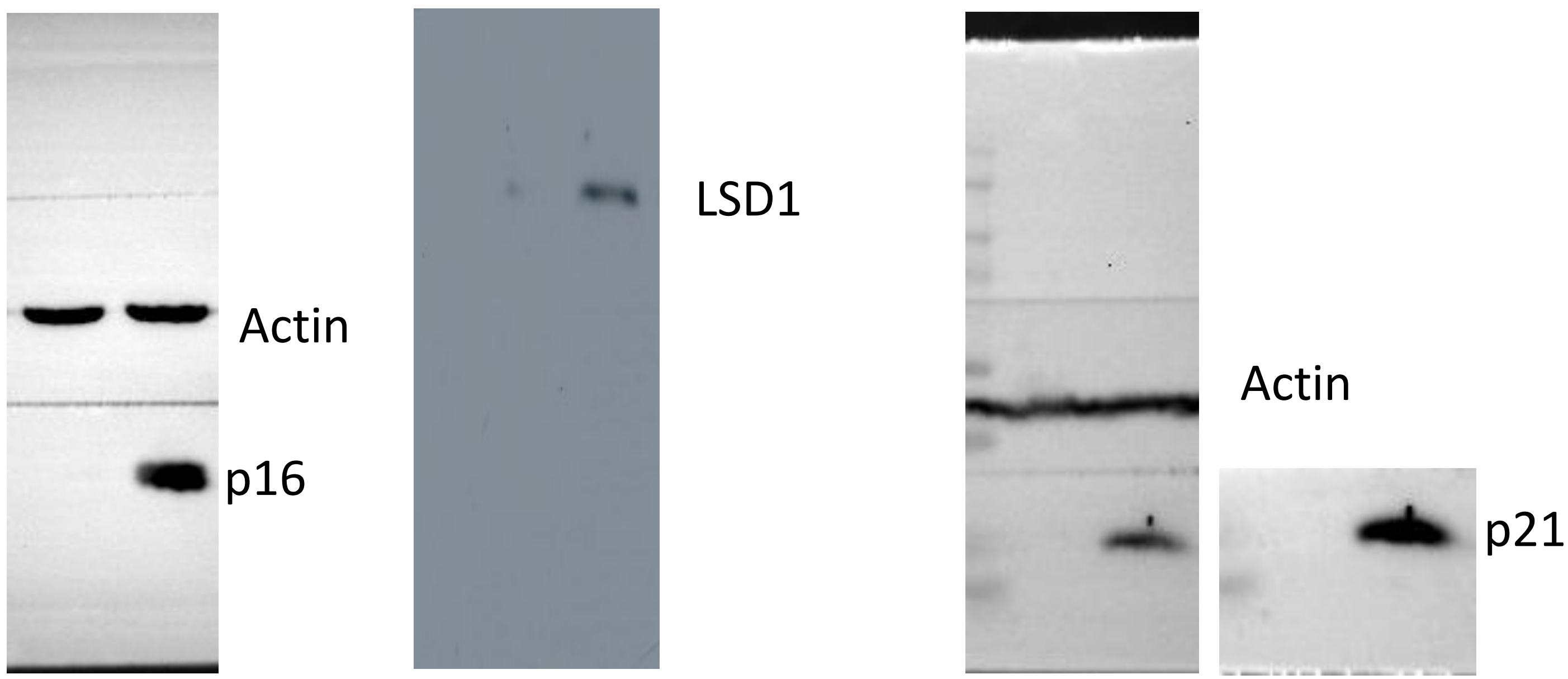

Sup Figure 3B

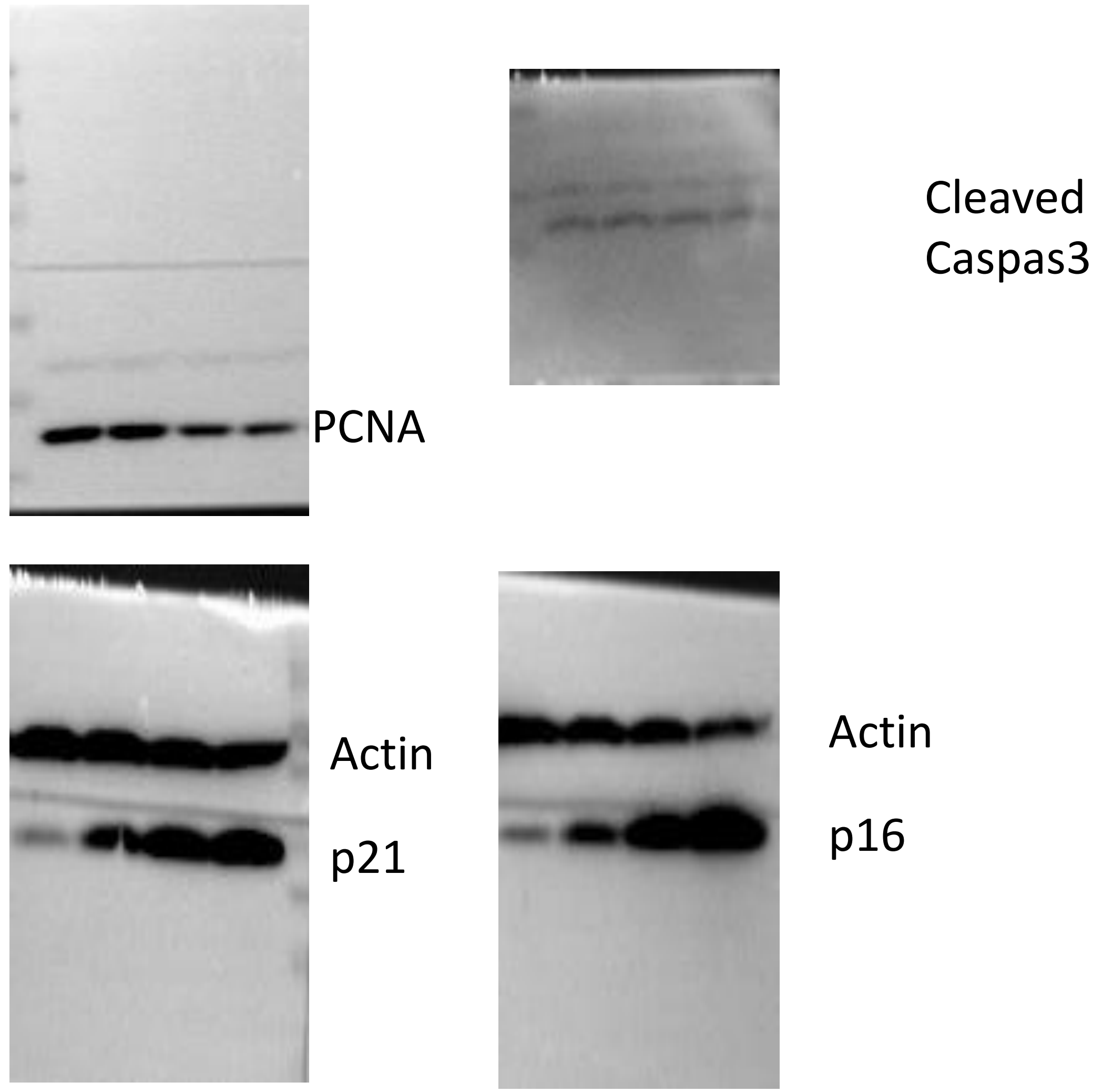

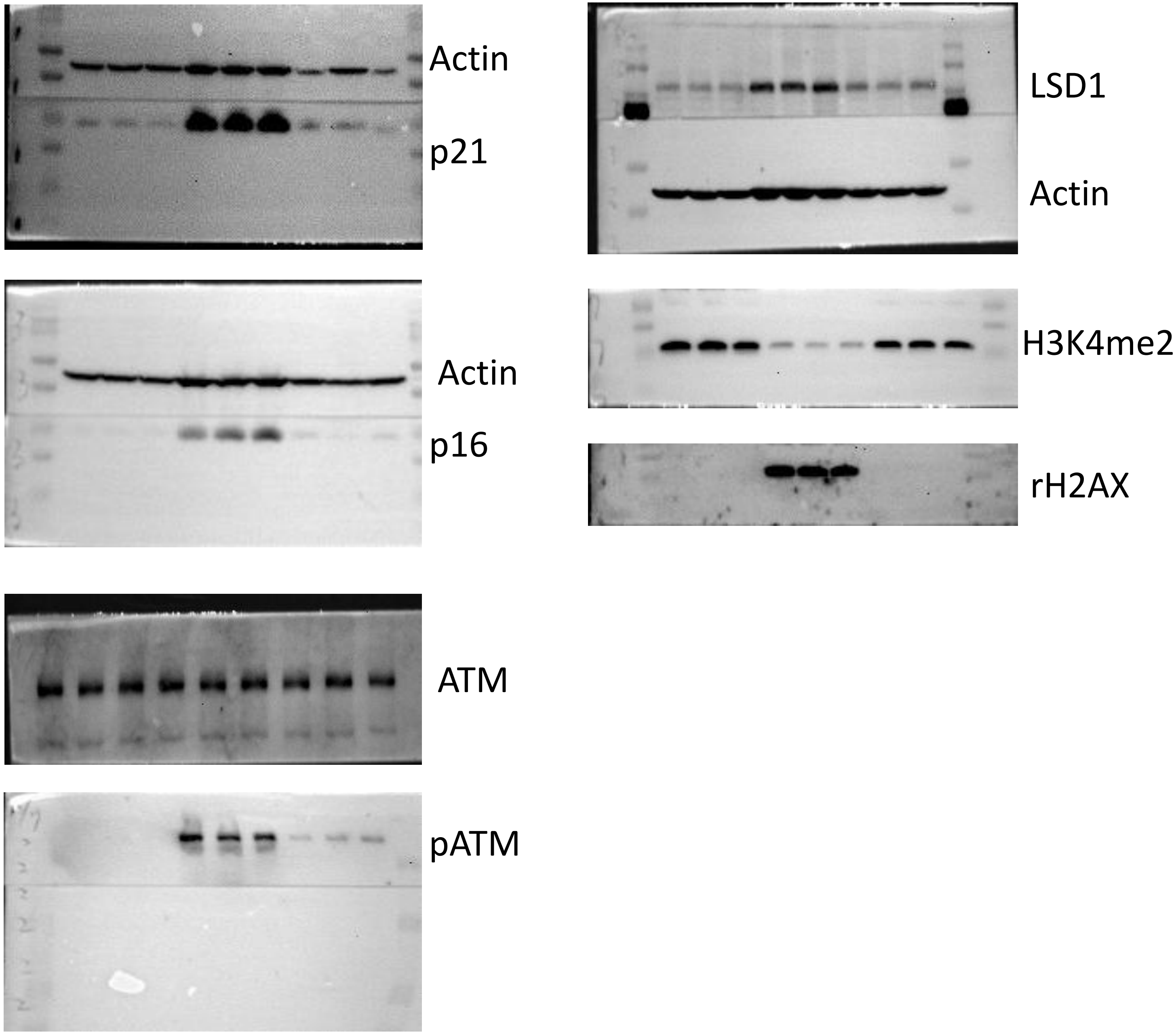

Supplementary Figure 4F (kidney)

Supplementary Figure 4F (heart)

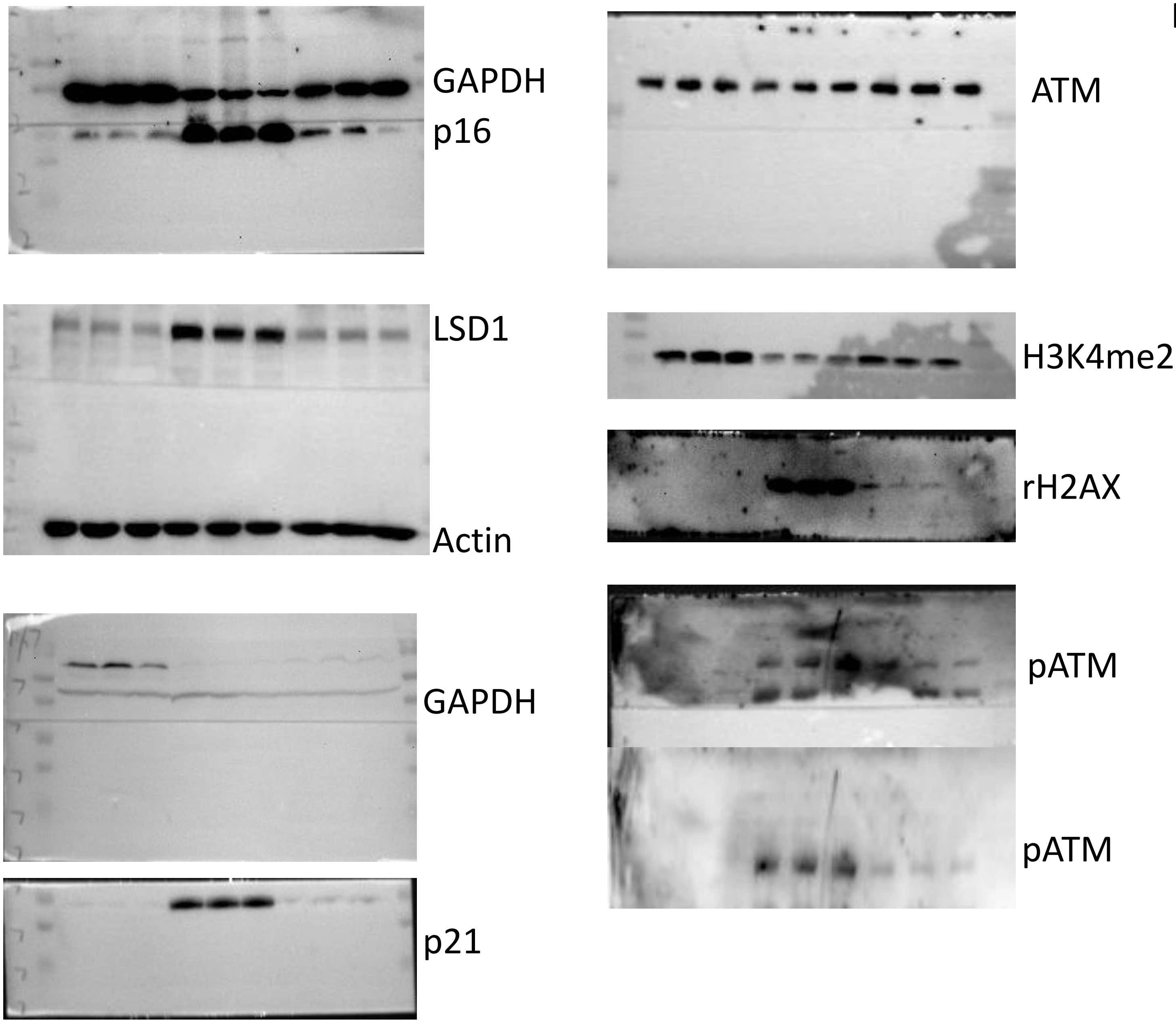

Supplementary Figure 4F (liver)

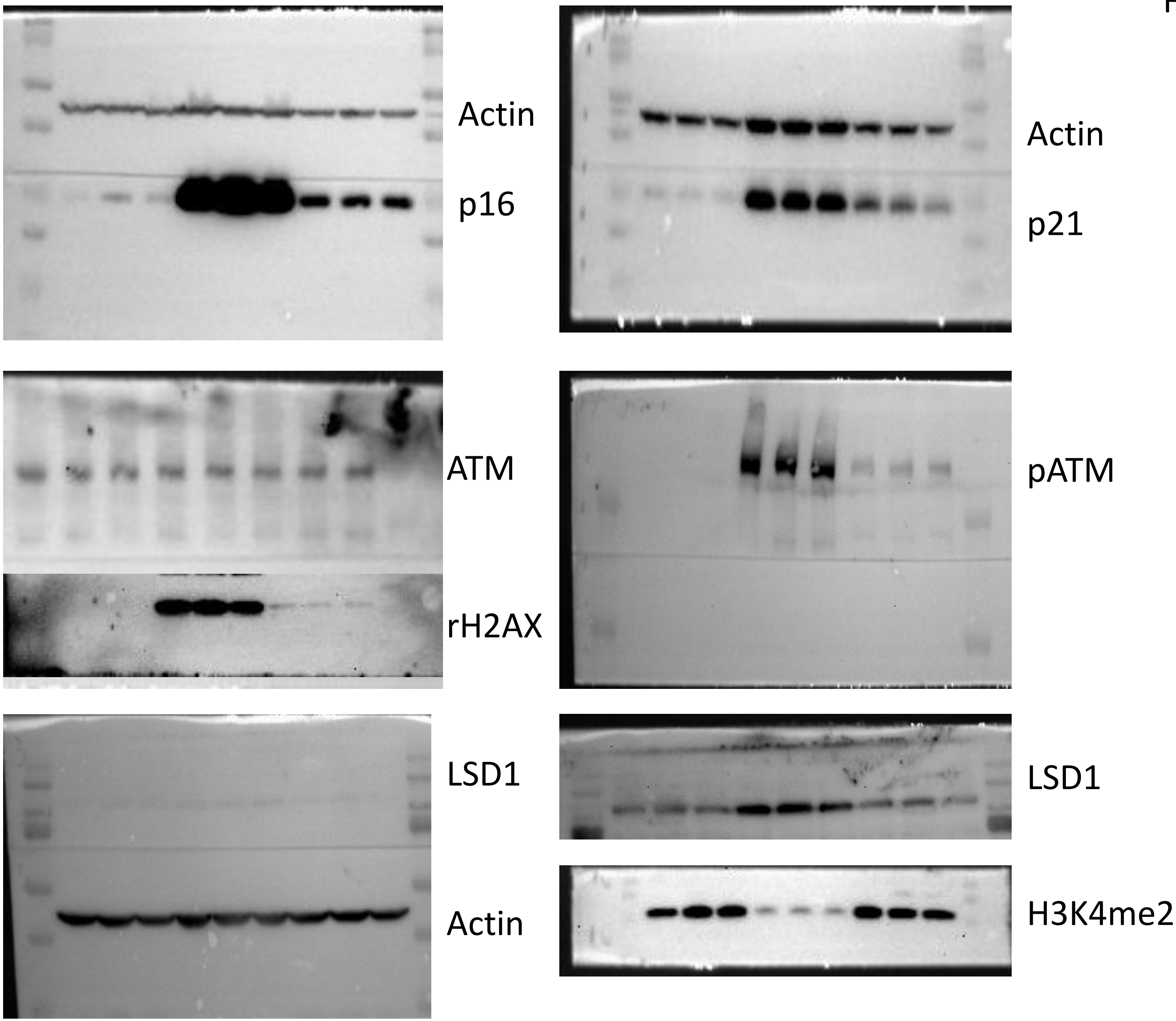

Sup Figure 5A

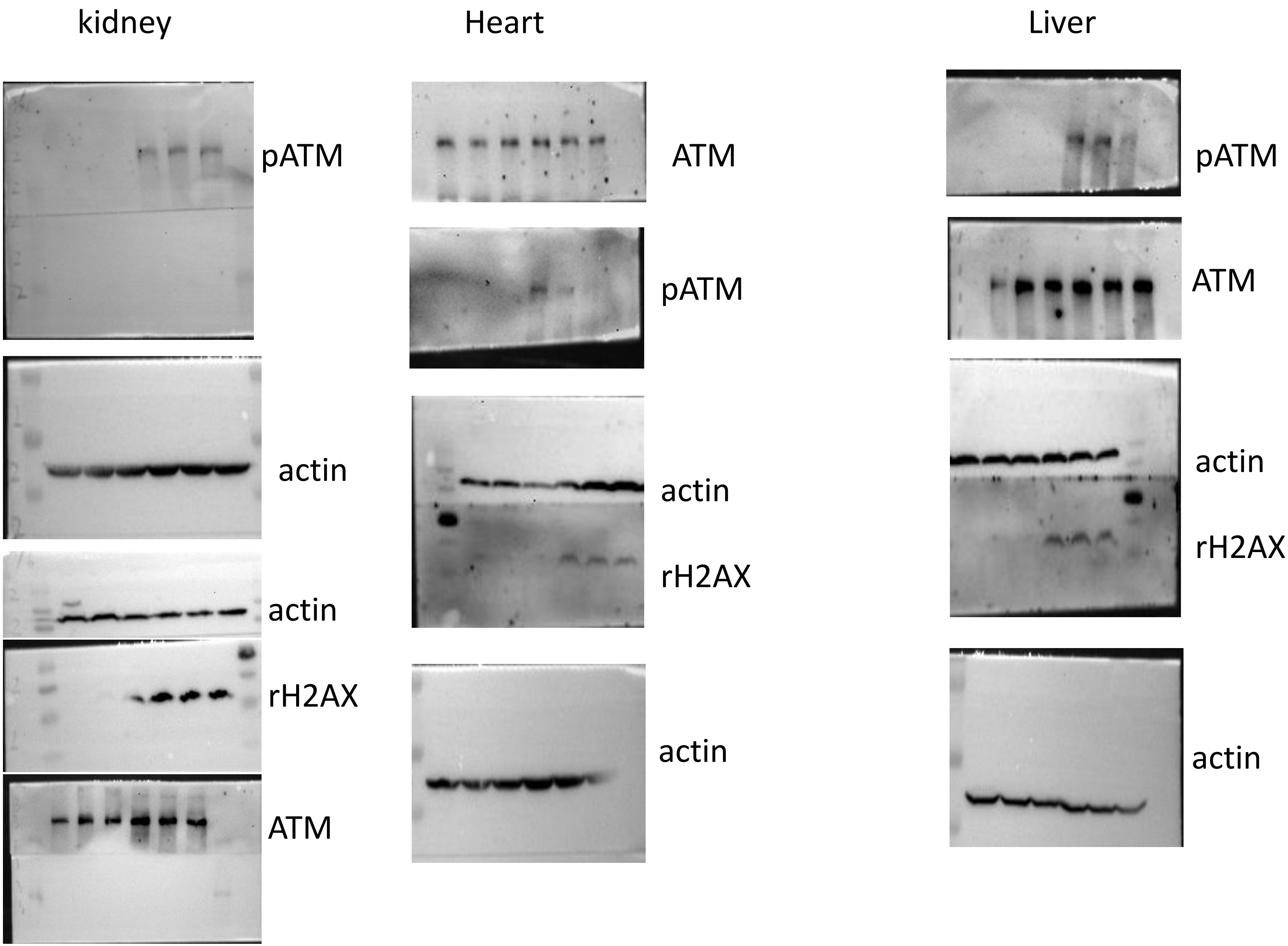

Sup Figure 5A

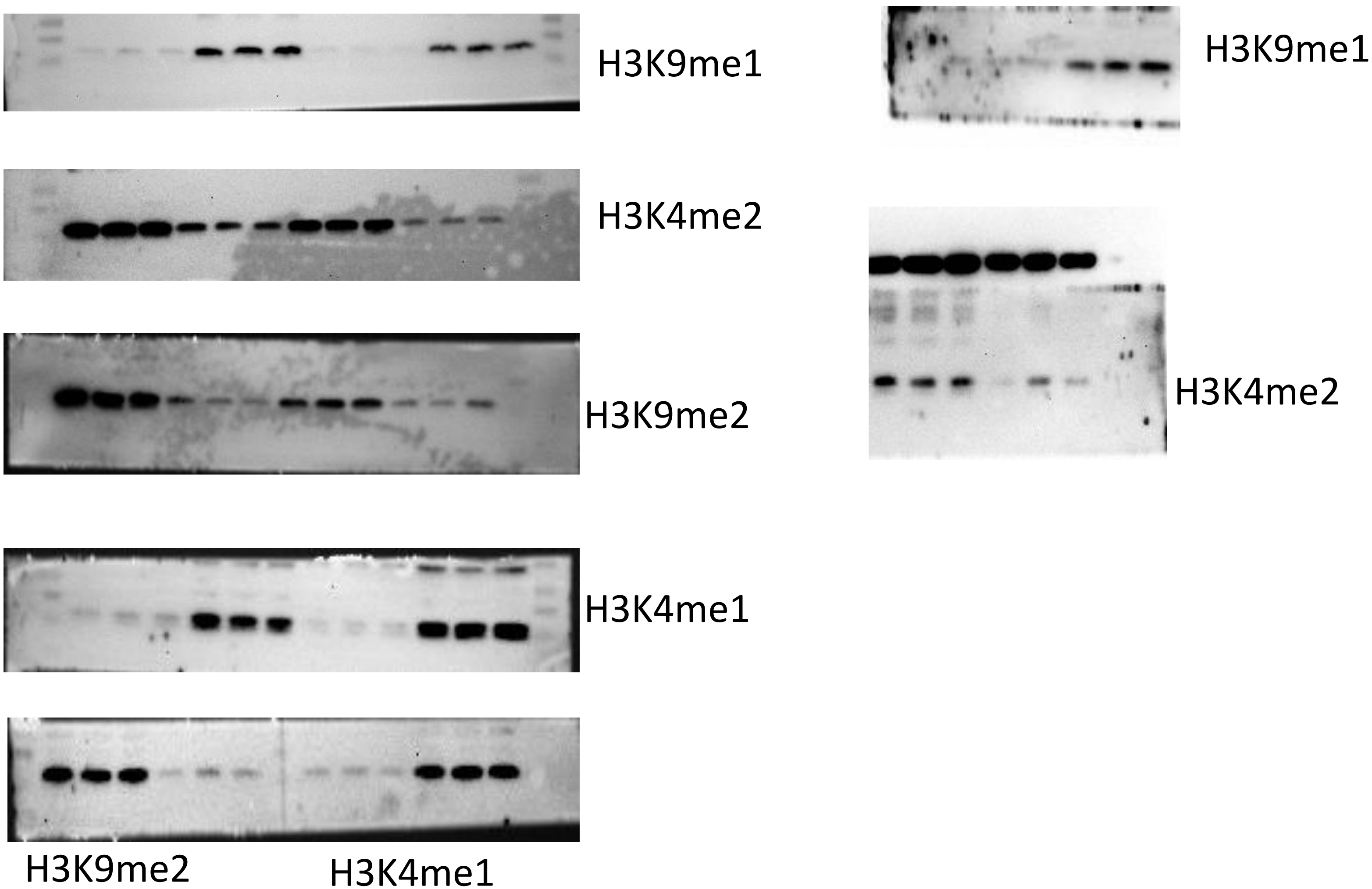

Sup Figure 5B

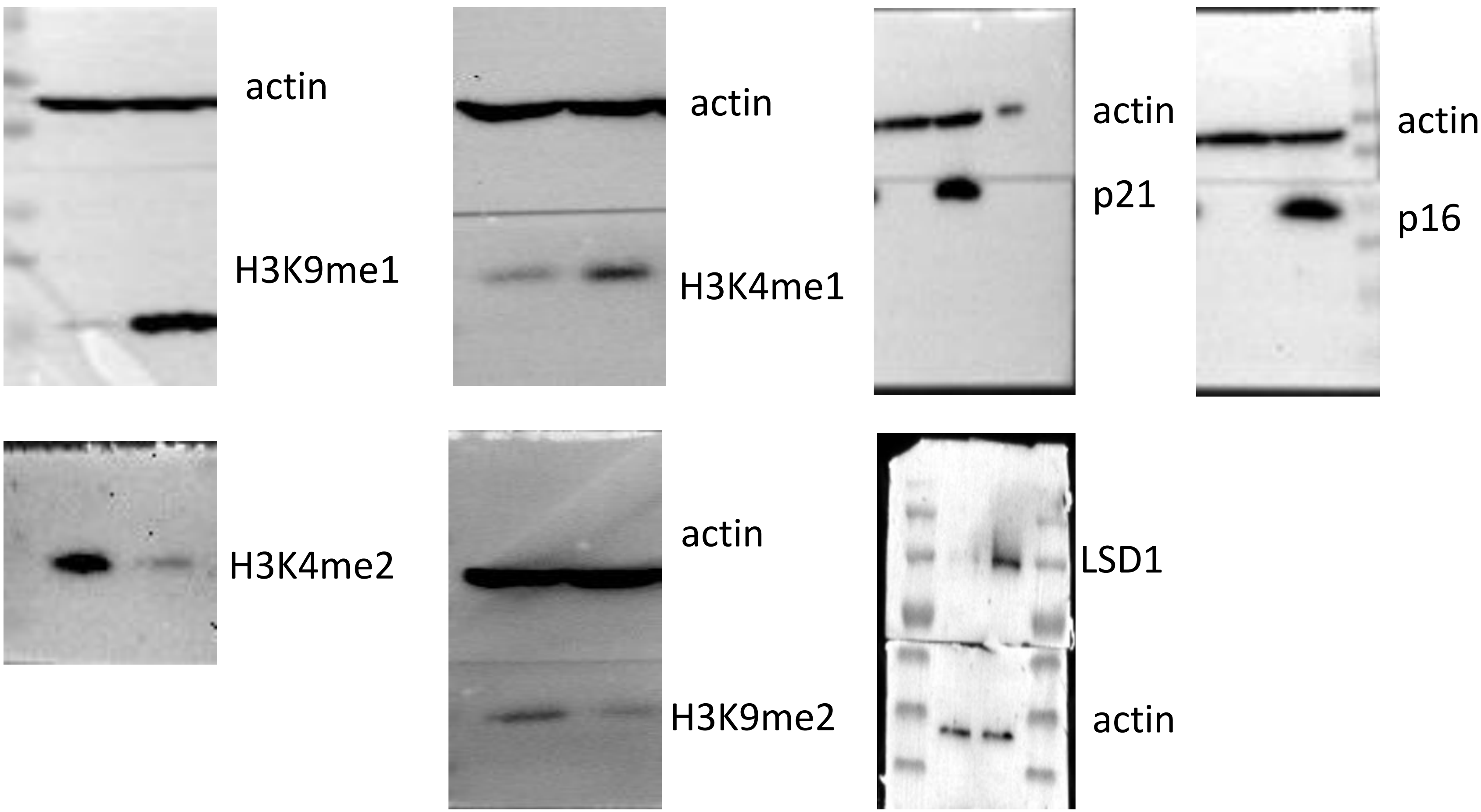

Sup Figure 6

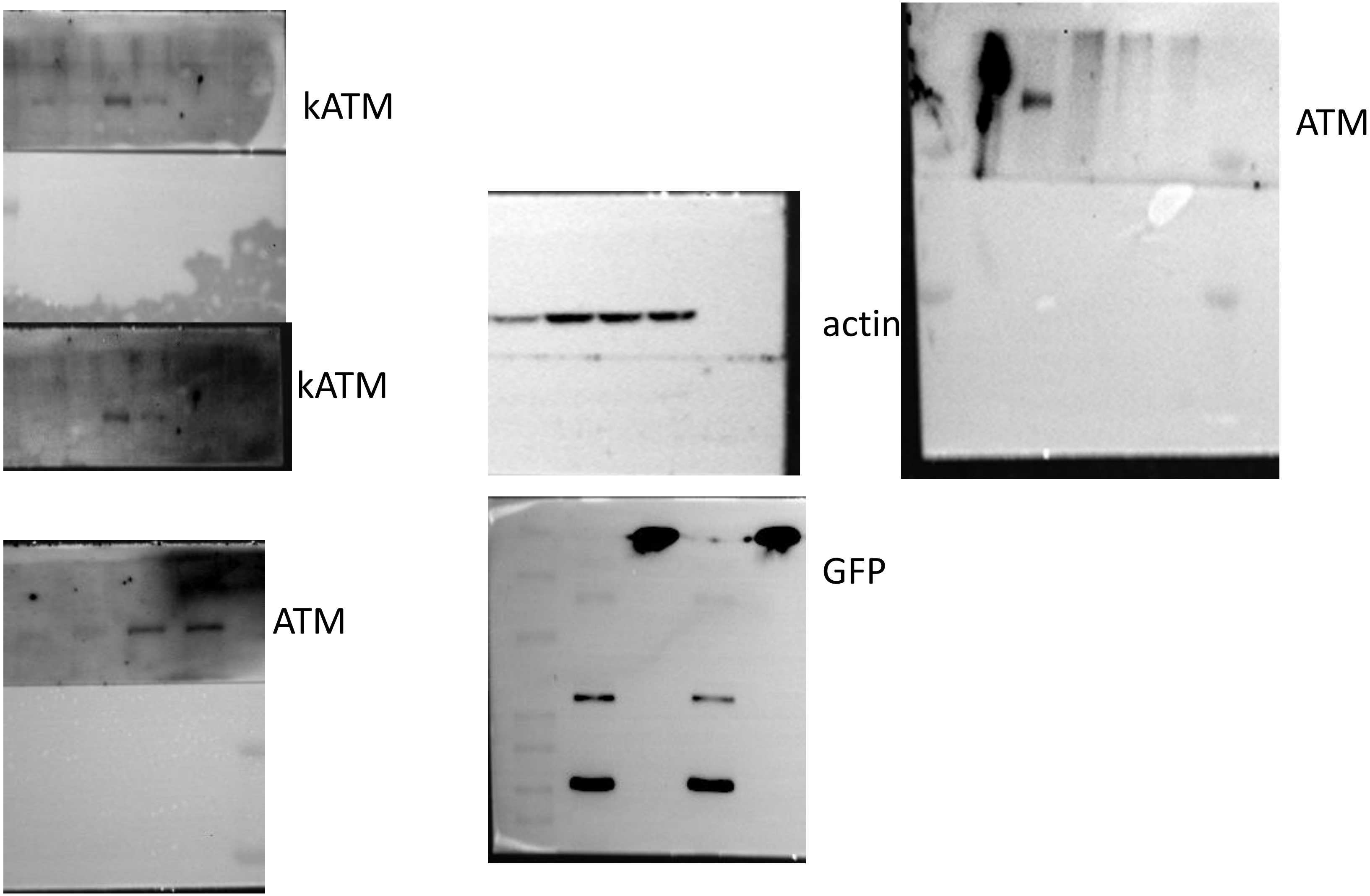

Sup Figure 7

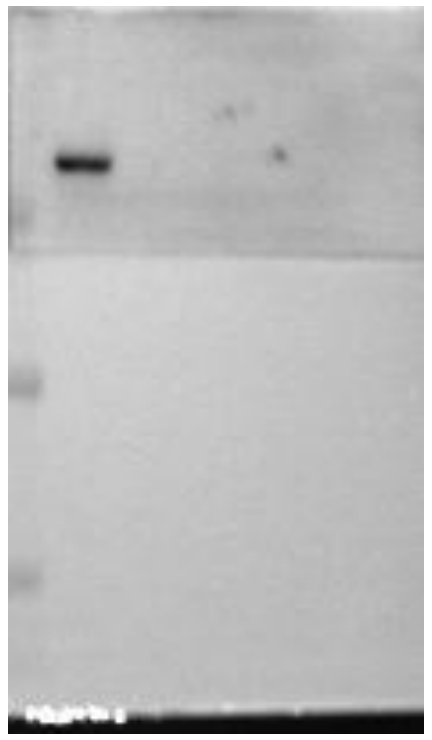

ATMk3016

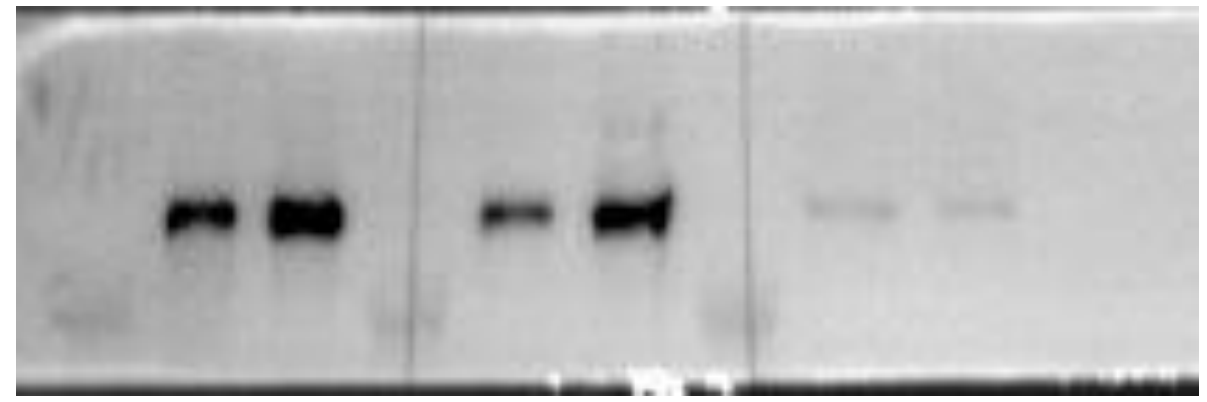

ATMK3016

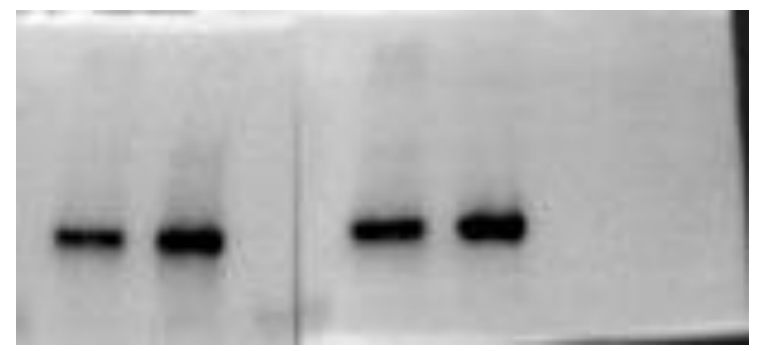

ATMK3016

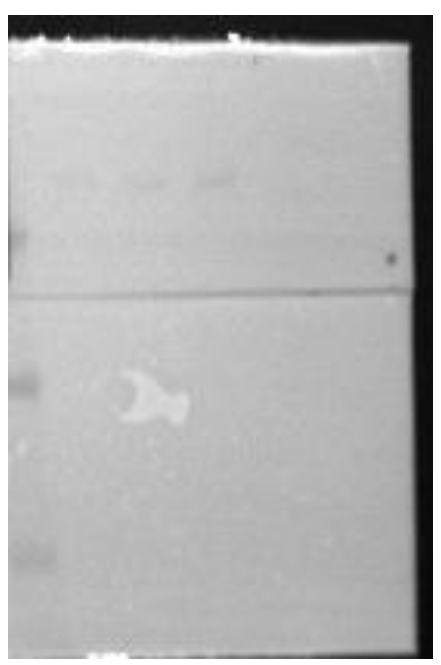

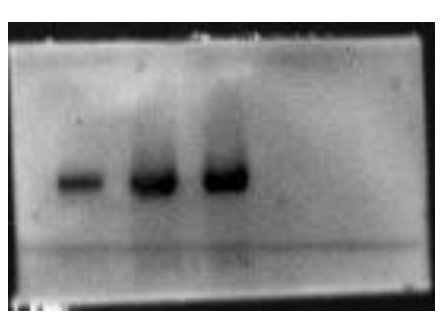

ATM

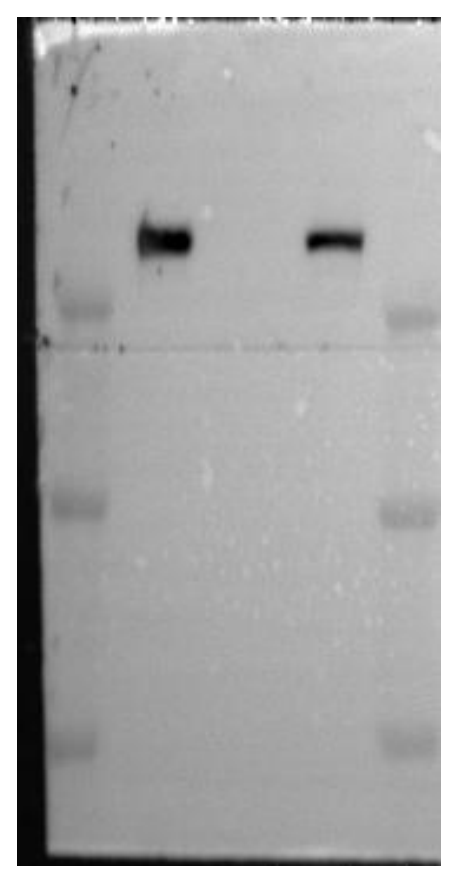

ATM

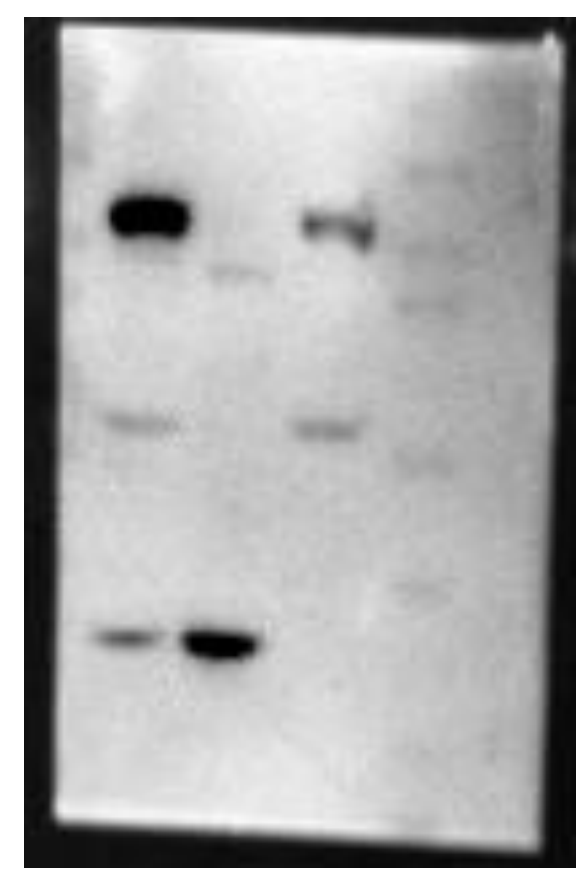

LSD1

Sup Figure 8A

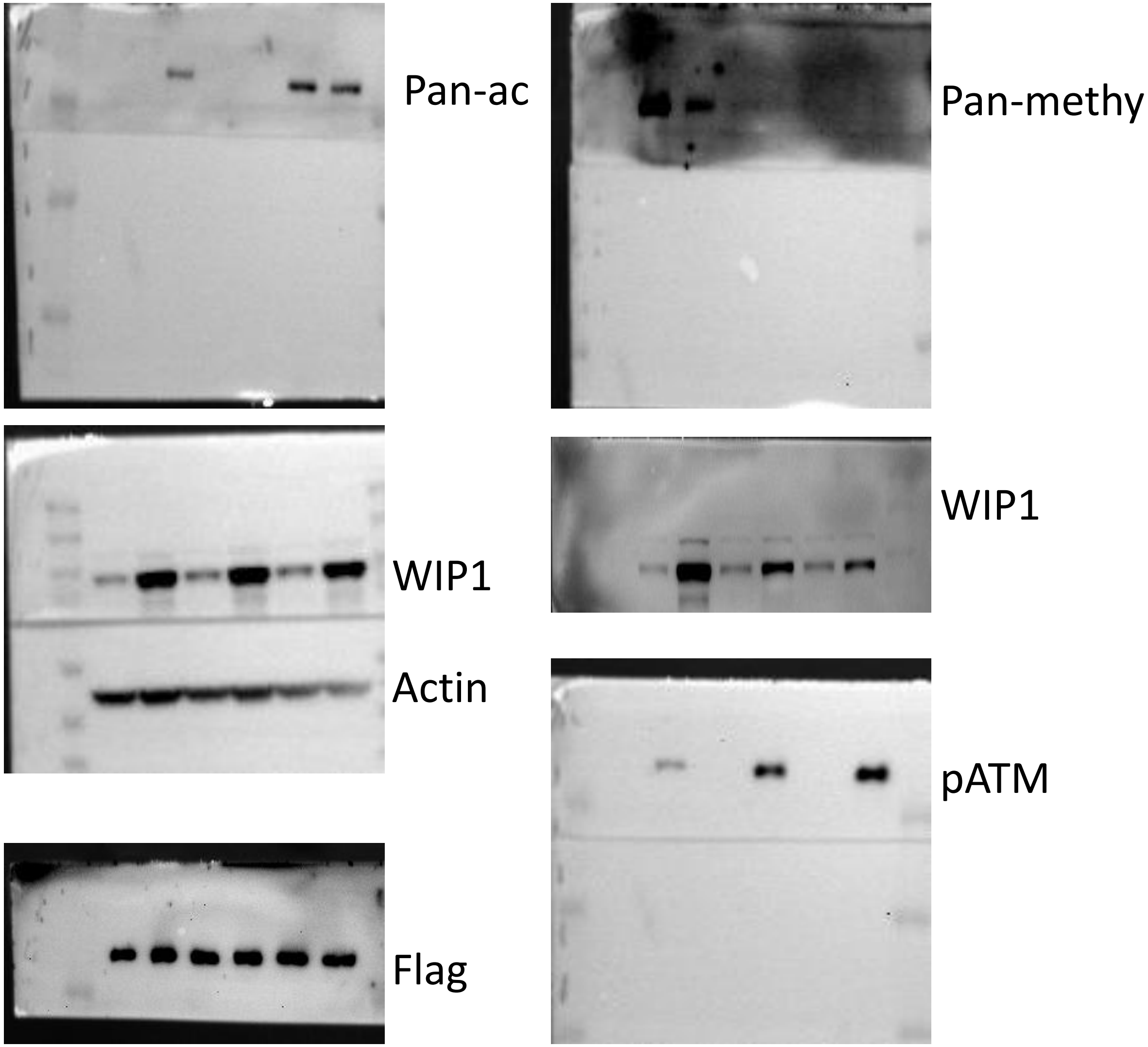

Sup Figure 8B

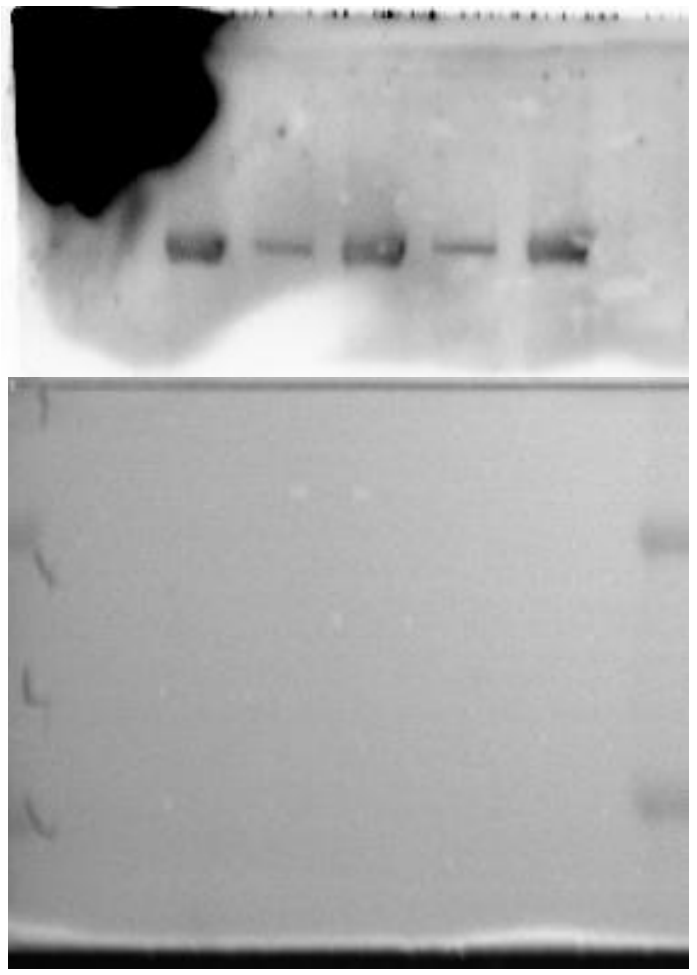

Pan-methy

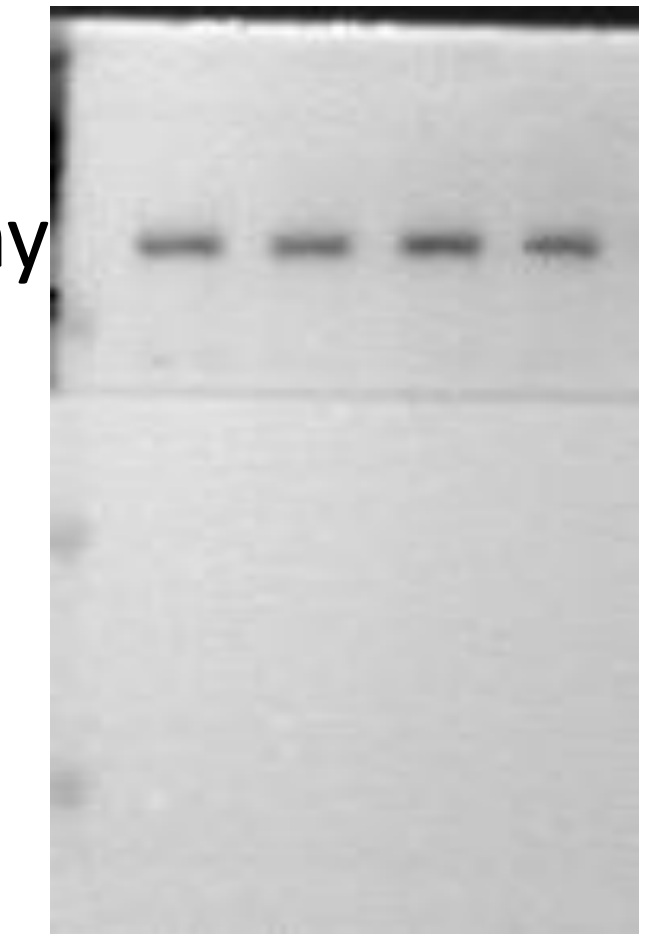

ATM

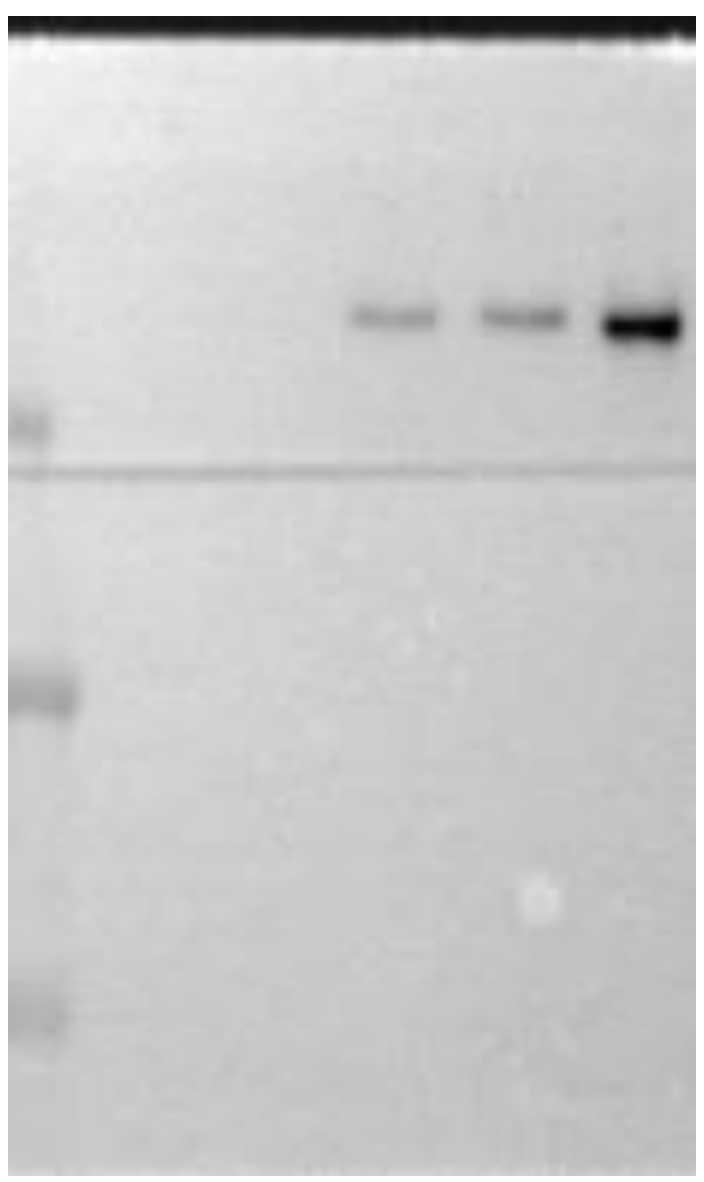

Pan-ac

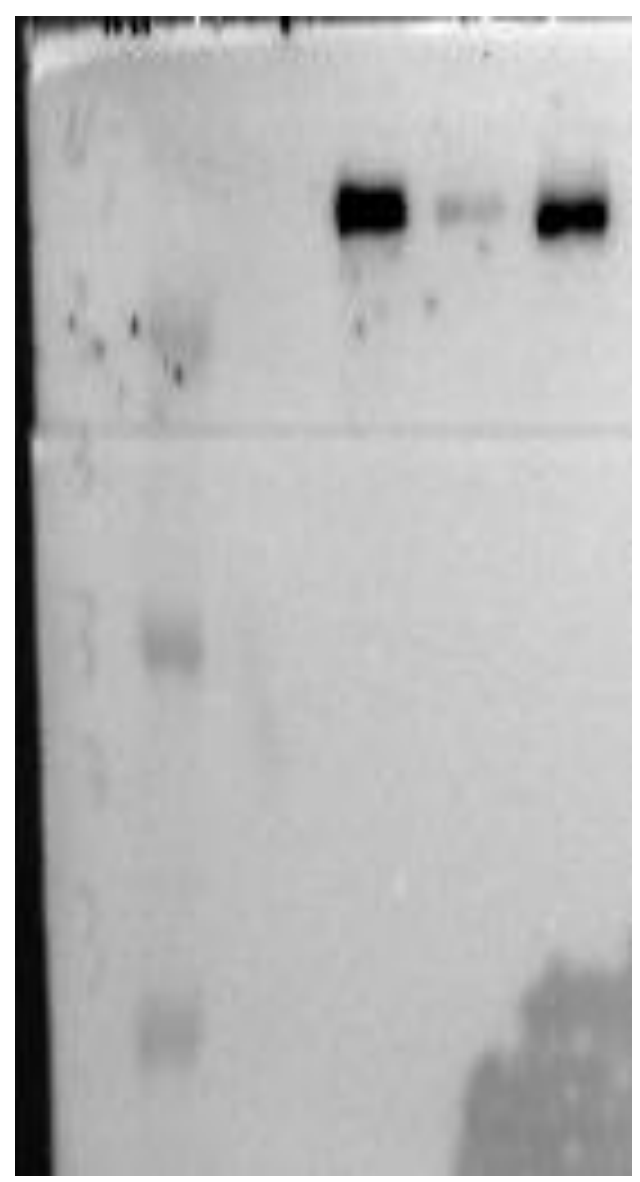

pATM

Sup Figure 9A

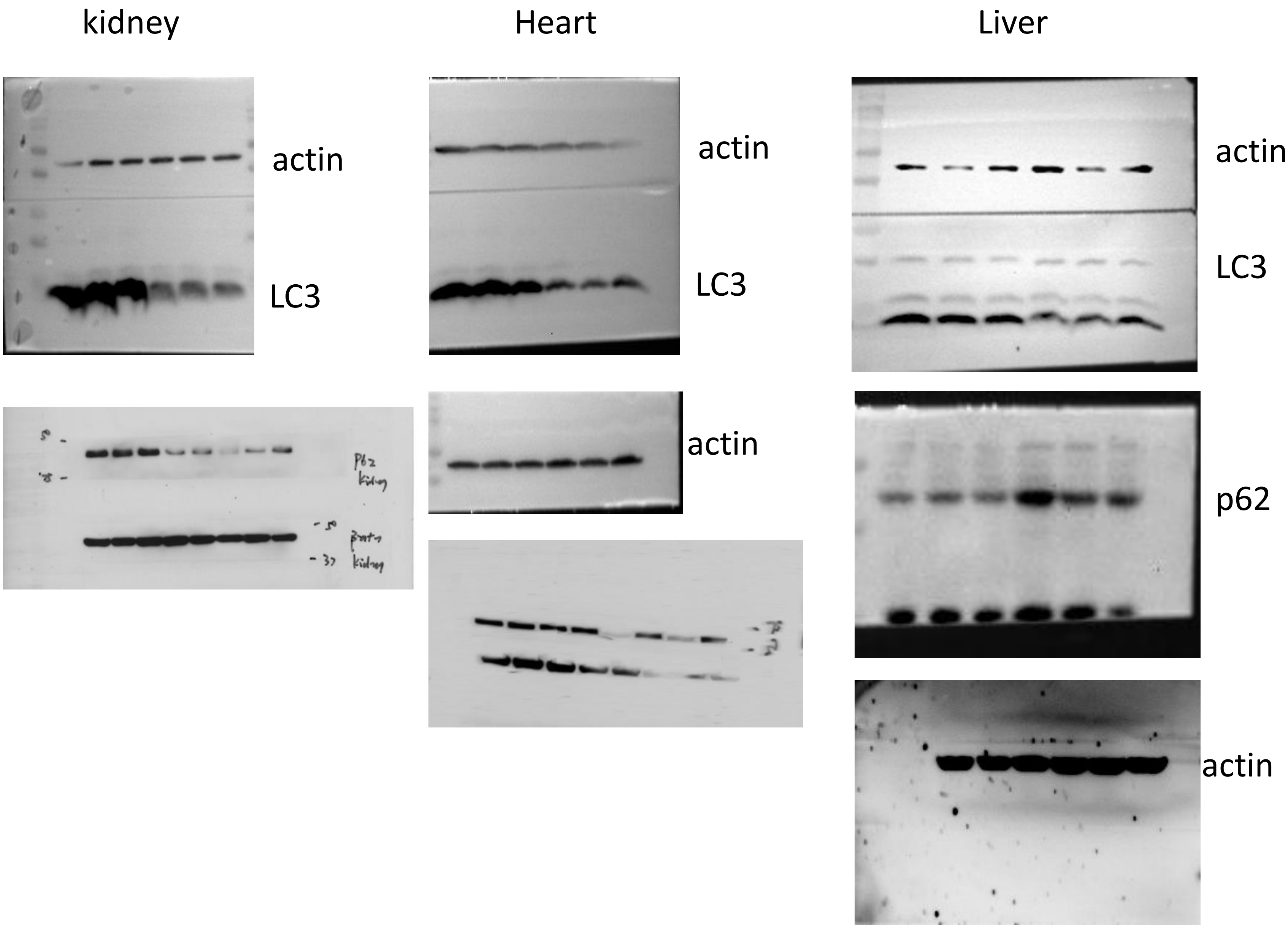

Sup Figure 9B

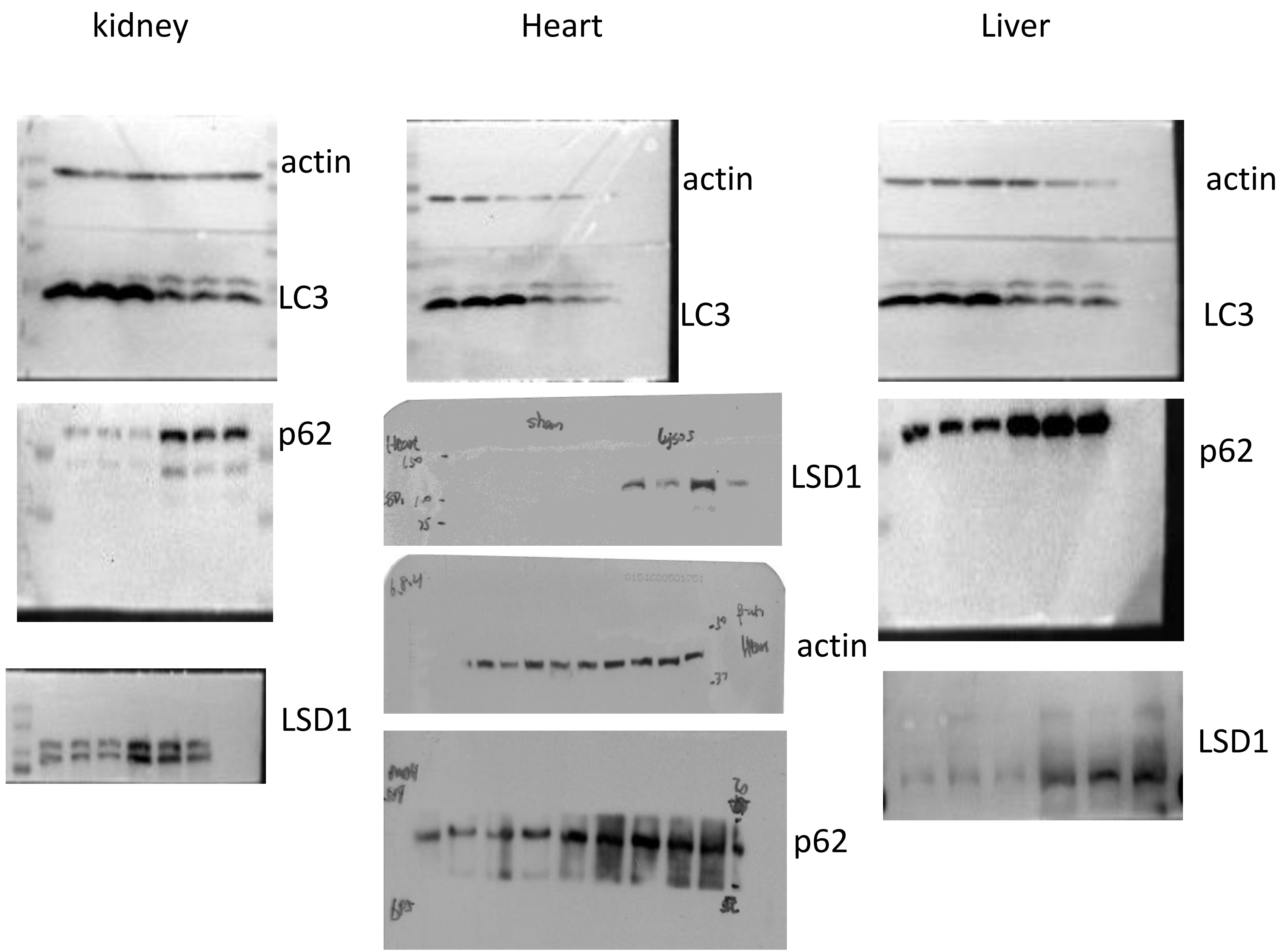

Sup Figure 9D

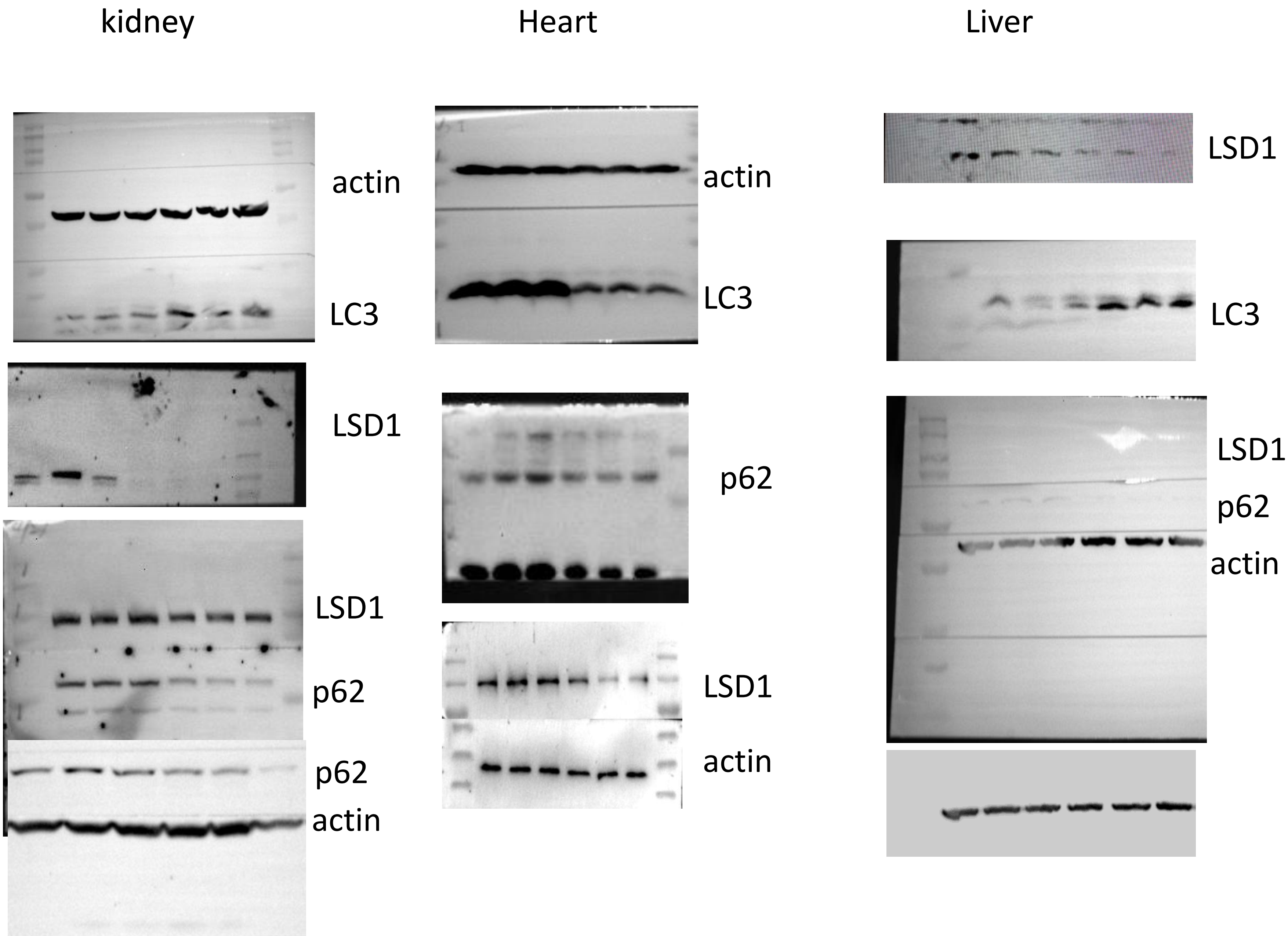

Supplement: Supplementary file 2 [file jci-136-199854-s218.pdf]
